# NOCI‑F Electronic Couplings in Assemblies of Indolonaphthyridine Molecules: From Dimers to the Full Stack

**DOI:** 10.1021/acs.jctc.5c01695

**Published:** 2026-01-20

**Authors:** I.-O. Stan, T. P. Straatsma, R. Broer, C. de Graaf, X. López

**Affiliations:** † 16777 Universitat Rovira i Virgili, Departament de Química Física i Inorgànica, Marcel·lí Domingo 1, Tarragona 43007, Spain; ‡ National Center for Computational Sciences, 6146 Oak Ridge National Laboratory, Oak Ridge, Tennessee 37831-6373, United States; § Department of Chemistry and Biochemistry, University of Alabama, Tuscaloosa, Alabama 35487-0336, United States; ∥ Zernike Institute for Advanced Materials, University of Groningen, AG Groningen 9747, The Netherlands; ⊥ ICREA, Pg. Lluís Companys 23, Barcelona 08010, Spain

## Abstract

Key electronic processes related to molecular excitonic states of finite stacks of indolonaphthyridine molecules are analyzed via the non-orthogonal configuration interaction with fragments (NOCI-F) method. Indolonaphthyridine is an organic chromophore that can undergo several electronic photoexcitation-related intermolecular processes, such as exciton and electron transfer. The structures studied here are noncrystalline arrangements built as either ordered stacks of indolonaphthyridine or stacks extracted from molecular dynamics simulations including thermal disorder. Taking dimers or trimers from either model, we performed CASSCF and NOCI-F calculations to quantify the intermolecular electronic couplings governing singlet fission, excited singlet and triplet diffusion, and hole and electron diffusion processes. Also, comparing the results for the different models, we studied the effect of structural disorder and distortion on these couplings. Finally, we present a newly developed, advanced postanalysis tool. It takes the NOCI-F data as input to carry out a multifragment full Hamiltonian procedure that involves the complete stack, providing physical information not available from the dimer/trimer models, hence giving access to additional insight into the material’s properties.

## Graphical abstract

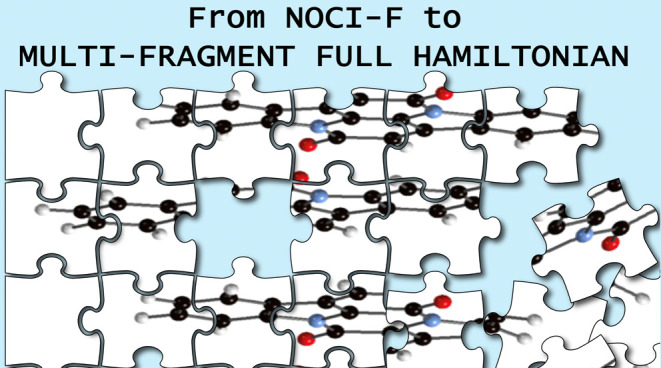

## 1 Introduction

Present-day concerns are pushing for strategies that look for more available, affordable and efficient materials to emerging or existing energy-related technologies. In this regard, the capture of solar energy^1–3^ with conventional inorganic materials is steadily increasing in terms of efficiency. However, these materials might be eventually partially replaced by alternative organic ones due to increasing commodity prices and/or exhaustion in the mid term. This evolution would be, in part, favored by the possibility of making massively available organic chromophores with superior light harvesting properties for sustainable energy production in solar cells.

Much experimental and theoretical research effort has put the focus on proposing, understanding and applying organic chromophores and their electronic properties for energy storage or clean energy production. Technological applications for solar light harvesting based on tailored materials, from environmentally friendly^4^ to natural^5,6^ or unconventional,^7^ occupy a prominent position in nowadays research. A particular phenomenon of application is multiple exciton generation (MEG),^8–12^ a process that begins with an incoming photon hitting a molecule or nanocrystal in its electronic ground state, generating an exciton (*S*_0_ → *S*_1_), i.e., a bound electron–hole pair, which evolves nonradiatively into multiple electron–hole pairs. Among the various mechanisms responsible for MEG, singlet fission (SF)^13^ occurs typically through intermolecular energy transfer.^14^ Conceptually, it occurs in two consecutive steps. First, an excited singlet state (*S*_1_) localized at one site or molecule transfers approximately half of its excess energy to a neighboring unit. By a radiationless transition to a lower lying local triplet state (*T*_1_) a similar triplet state on a neighbor is created. The spin moments of the two triplet states are coupled to an overall singlet state, making it a spin-allowed process. To complete SF, the two entangled triplet states (*T*_1_ *T*_1_) separates into two independent triplets

1 $$S_{1}S_{0}\to T_{1}T_{1}\to T_{1}\cdot \cdot \cdot T_{1}$$

Knowledge based on isolated molecules has led to the proposal of candidate compounds for SF, with several aspects being necessary to be considered for the enhanced design of SF solar cells.^15–17^ This process requires chemical stability, high absorptivity, long triplet lifetimes, and specific triplet as well as charge carrier transport properties.^18,19^ In addition, the energy difference between the *S*_0_ molecular ground state and the lowest triplet state must be approximately half the energy difference between *S*_0_ and the lowest excited singlet state.^20,21^ From this rationale, several candidates for SF have been identified.^22–26^ Understanding of the factors that govern this energy difference point to a balance between a certain amount of biradical character, responsible for a low-lying triplet state, and chemical stability, favored by a large HOMO–LUMO gap.^27–30^ These characteristics are typically met in planar organic molecules with large delocalized aromatic systems, such as indolonaphthyridine^25^ (INDO, see Figure 1, also known as cibalackrot when hydrogens in the central rings are replaced by phenyl groups).

**Figure 1:**
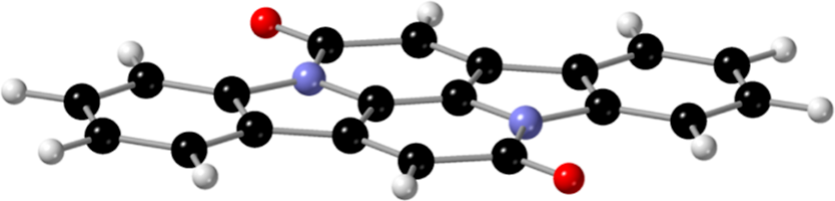
Indolonaphthyridine molecule (INDO). Black, white, blue and red spheres represent carbon, hydrogen, nitrogen and oxygen, respectively.

SF is one among multiple electronic processes that can take place in electronically excited organic chromophores. Other interesting phenomena include diffusion of the original excited singlet state (*S*_1_) to neighboring units, diffusion of a triplet state generated upon SF,^31^ generation of a charge-separated state from an excited singlet and, as a consequence of the latter, diffusion of a hole and/or an electron through the material. Figure 2 depicts the mentioned processes schematically for a model with three neighboring chromophores.

**Figure 2:**
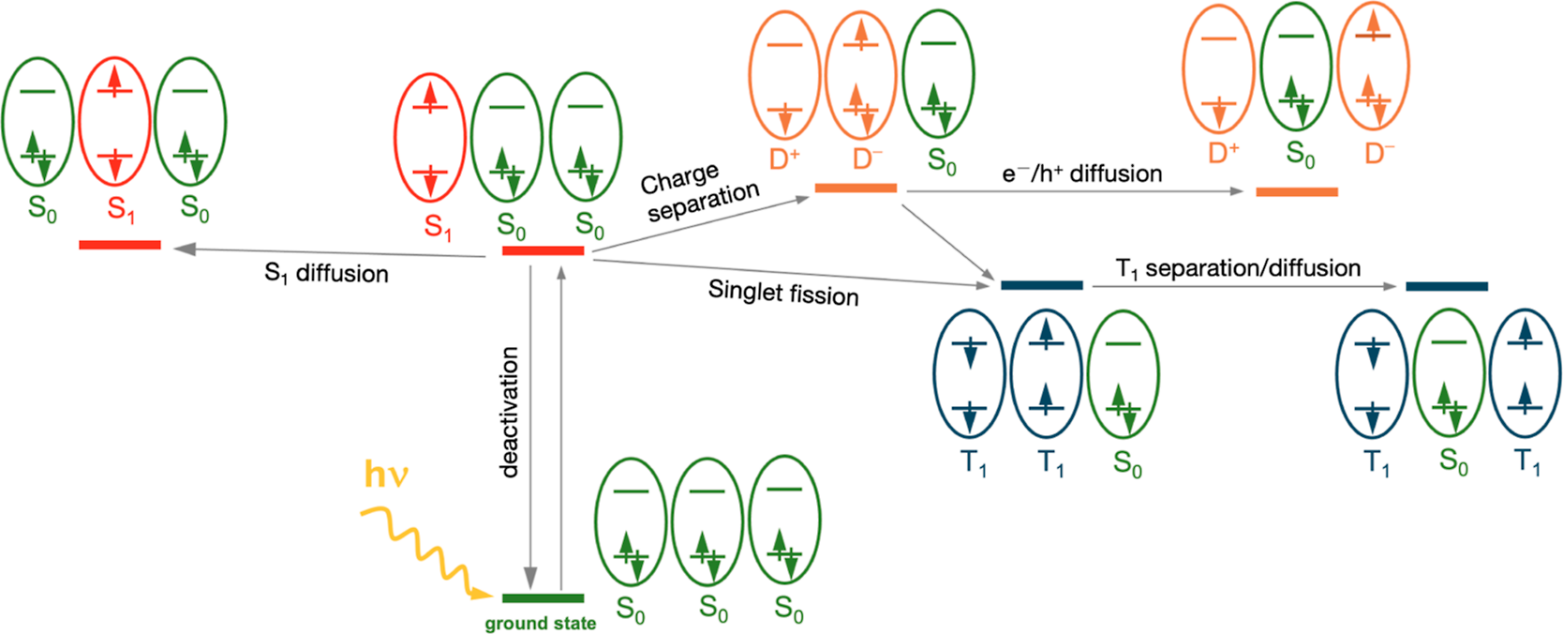
Scheme of part of the fate of a localized excited singlet state in a trimer, *S*_1_ *S*_0_ *S*_0_, where each oval represents a molecule (fragment) with a simplification of its local electronic configuration. e^–^ and h^+^ stand for electron and hole, respectively. Cationic (*D* ^+^) and anionic states (*D* ^–^) of fragments are considered. In all situations, the states of the three fragments couple to a total singlet.

Several works have reported SF and other electronic phenomena for molecular crystals of pure or functionalized acenes, (tetracene,^32^ pentacene^33–37^ ), perylene bisimide (PBI),^14,38–42^ indolonaphthyridine,^25^ Pechmann dyes^43^ and others.^44–50^ Working with crystalline materials offers several advantages, such as reproducibility, characterization, stability and control, which make them potentially attractive for large-scale applications. However, the rigid arrangement of molecular fragments governed by the crystalline packing has a considerable influence on the energy and electron transport properties.^51–54^ In this regard, alternatives to the broadly popular highly ordered materials exist,^55^ such as the innovative solution recently proposed by Olivier and co-workers. They reported a smart procedure to generate a noncrystalline ordered material with notable stability and interesting bulk-like electronic properties.^39,41^ The underlying idea of this approach is to generate stacks of organic units in liquid solution, resorting to π–π interactions, and locking in their positions in a reactive stapling process. Following this principle, new tailored materials with enhanced properties can arise, for example, as light-harvesting or charge-conducting materials.

The computational work herein presented focuses on getting insight in the potential of these structural arrangements by studying different models of INDO stacks. This choice, instead of perylene bisimide (PBI)—a variant of which is used as starting fragment in Paulino’s work^41^—is justified as it allows for slightly more affordable calculations due to the smaller total number of electrons in the INDO molecule, without compromising the goals of the work. The computational simulations reported here serve as starting point to future research in this topic.

As described in detail in Section 2.4, the non-orthogonal configuration interaction with fragments (NOCI-F) method^56–60^ as implemented in the GronOR code^57,59^ has been applied to study the coupling between several electronic states involving dimers or trimers of the INDO molecule. NOCI-F is a versatile computational strategy to obtain accurate information related to the couplings among the electronic states involved in intermolecular energy and electron transfer processes.

The article is organized as follows: Section 2 describes the models and computational procedures applied, including a proposal to efficiently solve the problem of getting information on a system that goes beyond the possibilities of a standard NOCI-F calculation. Section 3 contains the NOCI-F results for ordered and disordered INDO models, with comparisons to evaluate the impact of factors such as the model size and the nature of the molecules constituting it.

## 2 Computational Information

### 2.1 DFT and CASSCF/PT2 Monomer Calculations

The ground-state singlet (*S*_0_) equilibrium geometry of indolonaphthyridine was computed in gas-phase conditions with the density functional theory (DFT) implementation of Gaussian 16 software,^61^ with the B3LYP functional^62,63^ and a triple-ζ + polarization 6-311G** Pople basis set. The obtained optimized structure for the ground state shows a planar *C*_2*h*_ symmetry, as displayed in Figure 1. The geometry of *S*_0_ was used to calculate all the relevant electronic states of regular (undistorted) INDO.

Taking the optimized DFT geometry of *S*_0_, state-specific complete active space self consistent Field (CASSCF) and second-order perturbation theory (CASPT2)^64–66^ calculations were performed with OpenMolcas including an imaginary level shift of 0.2 hartree and no IPEA shift.^67,68^ For these calculations, we used ANO-RCC basis sets^69^ with (4s,3p,2d) and (3s,2p) contractions for second row elements and H, respectively. All but core electrons (1s^2^ for C, N and O) were included in the perturbational treatment of the dynamic electron correlation. The active space considered contains 8 π orbitals and 8 electrons for INDO, i.e. CAS­(8,8), to describe the two lowest singlets, *S*_0_ and *S*_1_, and the lowest-lying triplet, *T*_1_, states, while the cationic *D* ^+^ state has one electron less in the active space, for a CAS­(7,8) and the anionic *D* ^–^ has one electron more, for a CAS­(9,8). This choice of active spaces is supported by previous tests confirming that enlarging them does not change the main results.^37,70^

### 2.2 Molecular Dynamics Simulations

The simulations of the liquid phase are based on atomistic molecular dynamics calculations with explicit solvent using the GROMACS 2019.3 code^71,72^ under fully periodic boundary conditions and the AMBER99 force field. We generated INDO stacks from different simulated liquid solutions containing dimethylformamide (DMF) as solvent. The simulation box is cubic with dimensions of 6 × 6 × 6 nm^3^, and contains 10 INDO molecules and 1442 DMF molecules for a [INDO] = 77 mM. The AMBER99 force field accounts for the potential energy of the system as the sum of bonded interactions (bond stretching, angle bending, and dihedral torsions) and nonbonded interactions are described by pairwise additive 1-6-12 Lennard-Jones and electrostatic potentials, considering interactions between atoms separated by more than three bonds. Atomic charges were calculated employing the ChelpG method based on reproducing the molecular electrostatic potential.^61^ A 10 Å cutoff was used for both van der Waals and short-range Coulombic interactions; the latter were further treated using the particle–particle mesh Ewald (PME) method to incorporate long-range electrostatic interactions. Bond constraints involving hydrogen atoms were applied using the LINCS algorithm. The production runs simulated 50 ns at *NVT* canonical ensemble conditions at *T* = 300 K with a time step of 1 fs, maintaining the temperature through the velocity-rescaling thermostat coupling. Before production, all systems followed a multistep protocol: an initial energy minimization, followed by 1 ns under *NVT* conditions and 1 ns under *NPT* conditions to allow box size adjustment. The absence of drifts in the energy-time and temperature–time plots ensures the required stability during the 50 ns of the simulation (see Figure S1 in the Supporting Information).

### 2.3 Structural Models

This work analyzes model structures of different complexity, from dimers of optimized INDO fragments to thermally disordered large stacks.

#### 2.3.1 Ordered Structures

Taking the DFT-optimized INDO geometry, the following models of parallel fragments were generated, all of them characterized by intermolecular distances Δ*z* = 3.5 Å.

- Dimers. In the following cases, the mutual rotation angle about the axis perpendicular to the molecular planes was varied between 0 and 90° (Figure 3).
- on-top. No intermolecular slippage, Δ*x* = Δ*y* = 0.0.
- slip-I. Intermolecular slippage, Δ*x* = 2.6 Å, Δ*y* = 1.0 Å.
- slip-II. Intermolecular slippage, Δ*x* = 1.0 Å, Δ*y* = 2.6 Å.
- Stack 1. Staircase ordered stack of 10 INDO units. The arrangement follows the slip-II pattern with no intermolecular rotation. See Figure S4 in the Supporting Information.
- Stack 2. Zig-zag ordered stack of 10 INDO units. The arrangement follows the slip-II pattern with a 20° rotation between first neighbors. See Figure S5 in the Supporting Information.

**Figure 3:**
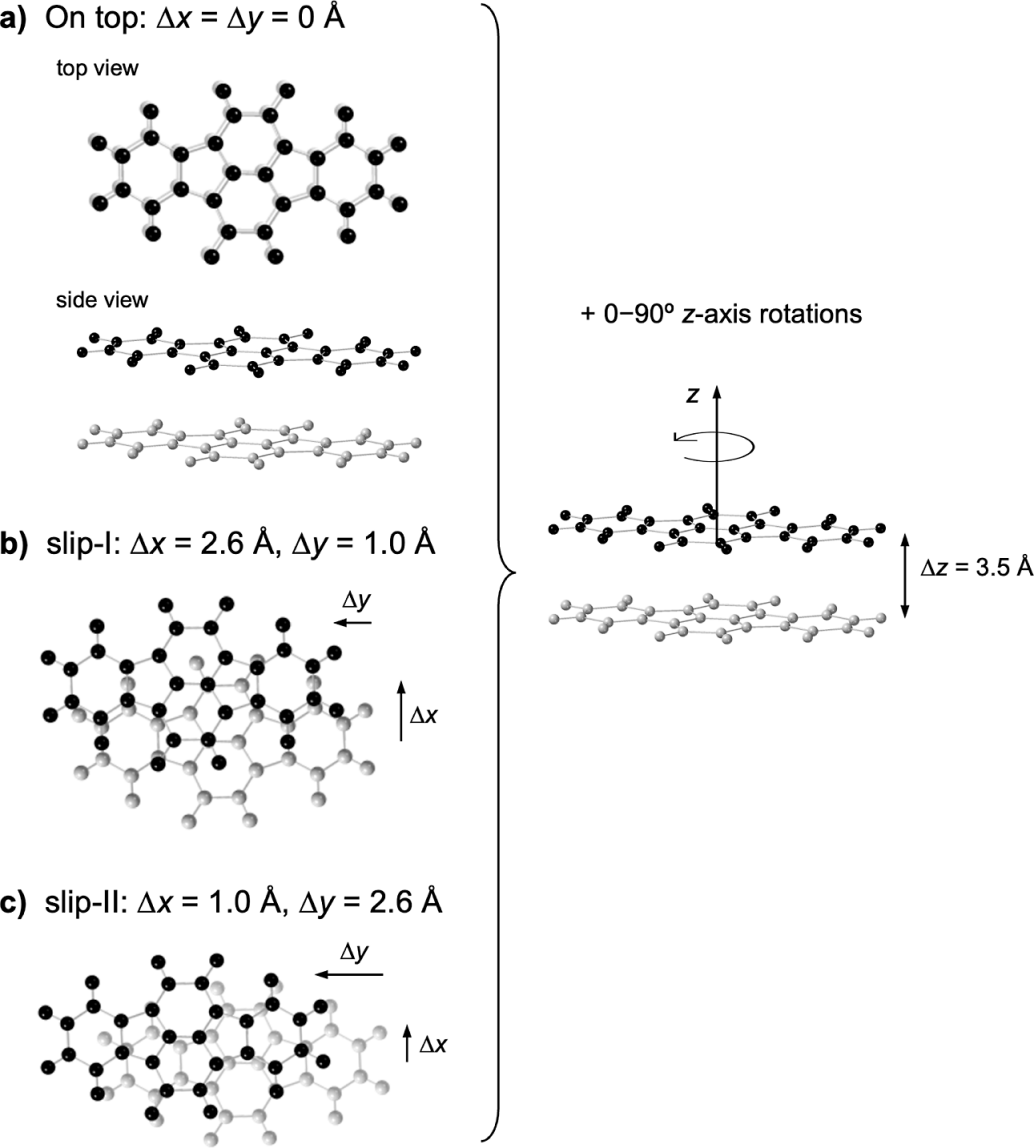
Representation of the three models of planar INDO dimers with parallel fragments at 3.5 Å distance. Case (a) corresponds to parallel on-top fragments, (b,c) to fragments’ center of masses mutually displaced as indicated by Δ*x* and Δ*y*. Each model includes rotations of one fragment about the *z*-axis. See text for further details.

#### 2.3.2 Disordered Structures

From molecular dynamics (MD) trajectories we obtained 8 to 10-fragment stacks, selected from the last 10 ns of the simulations, presenting intramolecular distortions and intermolecular disorder (see Section S8 in the Supporting Information). The following models for NOCI-F calculations were generated:

- Stack 3. A 9-unit stack generated from a MD simulation with a starting configuration of stacked INDO units with DMF solvent. Taken at simulation *t* = 49.5 ns (Table S31).
- Stack 4. A 10-unit stack generated from a MD simulation with a starting configuration of randomly distributed INDO units with DMF solvent. Taken at simulation *t* = 45.0 ns (Table S32).
- Stack 5. A 8-unit stack generated as the previous one. Taken at simulation *t* = 49.5 ns (see Figure 4 and Table S33).
- Stack 6. It is a modification of Stack 5 made replacing the distorted INDO units by their optimized (planar) counterparts (Table S34).

**Figure 4:**
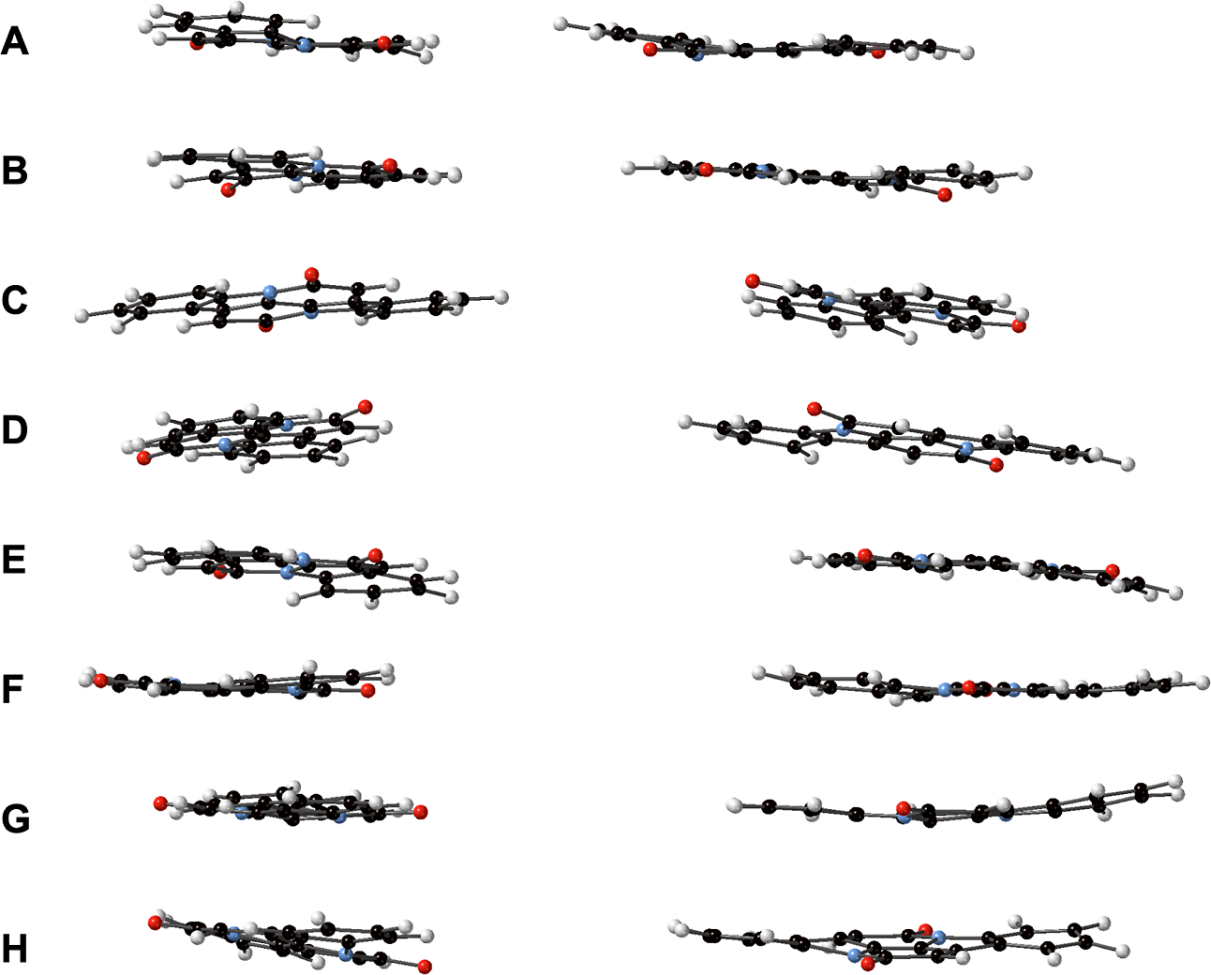
Two views of Stack 5, made of INDO units. Coordinates can be found in Table S33 in the Supporting Information.

By taking dimers and trimers from Stacks 5 and 6 we obtained the results presented in Section 3.3.

### 2.4 Non-Orthogonal Configuration Interaction with Fragments

As a continuation of our ab initio studies on SF in organic molecules,^37,42,73^ NOCI-F calculations are herein applied to INDO as SF candidate, focusing on electronic processes of interest related to light absorption involving dimers or trimers. As a central process, SF starts with the conversion of a localized excited singlet state into two neighboring triplet states (*S*_1_ *S*_0_ → *T*_1_ *T*_1_) and is followed by the separation of the singlet coupled double triplet in two independent triplet excitons (*T*_1_ *T*_1_ → *T*_1_···*T*_1_). Other interesting processes related to the localized singlet exciton are excited singlet diffusion (*S*_1_ *S*_0_ → *S*_0_ *S*_1_), charge separation (*S*_1_ *S*_0_ → *D* ^+^ *D* ^–^), triplet diffusion (*T*_1_ *S*_0_ → *S*_0_ *T*_1_), or hole (*D* ^+^ *S*_0_ → *S*_0_ *D* ^+^) and electron (*D* ^–^ *S*_0_ → *S*_0_ *D* ^–^) diffusion processes.

The NOCI-F procedure starts with the calculation of CASSCF (or other multiconfigurational) wave functions for a set of electronic states on the molecules (fragments) that are relevant in the employed physical model (see Section 2.1 above). Remarkably, we obtain electronic states that are each expressed in their own optimal orbital set. This is especially relevant for ionic states since orbital relaxation with respect to the ground-state orbitals is notable.

With a structural dimer (or trimer) model, these fragment wave functions are used to construct the many-electron basis functions (MEBFs) for dimer (or trimer) electronic states. MEBFs are spin-adapted antisymmetrized products of fragment wave functions. For a dimer, the relevant ensemble states (coupled to a total singlet spin) are the ground state, *S*_0_ *S*_0_, and the excited states *S*_1_ *S*_0_, *S*_0_ *S*_1_, *T*_1_ *T*_1_, *D* ^+^ *D* ^–^ and *D* ^–^ *D* ^+^. For trimers, we considered the following 13 relevant ensemble electronic singlet states: the ground state (*S*_0_ *S*_0_ *S*_0_), three local excited singlets (*S*_1_ *S*_0_ *S*_0_, *S*_0_ *S*_1_ *S*_0_, *S*_0_ *S*_0_ *S*_1_), two coupled triplets (*T*_1_ *T*_1_ *S*_0_, *S*_0_ *T*_1_ *T*_1_), the *T*_1_···*T*_1_ state (*T*_1_ *S*_0_ *T*_1_) and six charge transfer states (*D* ^+^ *D* ^–^ *S*_0_, *D* ^–^ *D* ^+^ *S*_0_, *S*_0_ *D* ^+^ *D* ^–^, *S*_0_ *D* ^–^ *D* ^+^, *D* ^+^ *S*_0_ *D* ^–^, *D* ^–^ *S*_0_ *D* ^+^), where the last two are the states hypothesized by Wang et al.^74^ to be responsible for enabling the formation of the *T*_1_···*T*_1_ directly from the *S*_1_ without passing through the *T*_1_ *T*_1_ state.

The Hamiltonian and overlap matrix elements among the MEBFs can be used to calculate the electronic coupling (γ_if_) between the initial (i) and final (f) states of any of the energy or electron transfer processes considered here (cf. eq 2)­

2 $$\gamma_{\text{if}}=\frac{H_{\text{if}}-\frac{1}{2}\left(H_{ii}+H_{ff}\right)S_{\text{if}}}{1-S_{\text{if}}^{2}}$$

This NOCI-F approach applied to electronic couplings involving ensembles of molecules, in addition to including intramolecular static and dynamic electron correlation effects^73^ and full orbital relaxation, has the advantage of expressing the final electronic wave functions of the whole ensemble as a combination of a few clearly identifiable MEBFs, facilitating the interpretation of the results. This combination of accuracy and simplicity is highly advantageous, although a higher computational complexity is the price to pay. Each matrix element of the NOCI-F Hamiltonian is the sum of the contributions of thousands of antisymmetrized Slater determinant products from both the *bra* and *ket* functions, and the calculation of each of these contributions is significantly more complex than in the case of orthogonal determinants. NOCI-F has been implemented in the massively parallel and GPU accelerated computer code GronOR.^57,59^ Combined with the application of a reduced common molecular orbital basis,^58^ the method can be used to study in great detail ensembles as large as trimers of molecules that are relevant to SF. More information on the NOCI-F approach and technical details of the computation of the matrix elements and the construction of the reduced common molecular orbital basis can be found in refs 56–60 .

For SF and exciton diffusion processes, we typically determine the electronic couplings between the initial and final states by applying eq 2 incorporating the effect of charge transfer configurations (charge transfer enhanced coupling, CT-enhanced). For this purpose, a new set of MEBFs is constructed through partial diagonalizations of the original NOCI-F matrix. For the SF case, these diagonalizations involve (i) the singlet excitons and the CT states and, (ii) the double triplet and the CT states. The eigenvectors of the partial diagonalizations with the largest weight on the singlet excitonic and double triplet states define the new set of CT dressed MEBFs for which the coupling is calculated by applying eq 2. Section S2 in the Supporting Information gives a compact description of the procedure for calculating CT enhanced couplings. A more extended explanation was recently published by some of us.^37^ We refer to these resulting couplings throughout this article.

To keep the interpretation of the NOCI wave functions of the stacks as straightforward as possible, we chose the phase of the fragment orbitals to be the same on all fragments in the models based on ordered planar units and on disordered planar units by performing translations and (small) rotations of the wave functions of fragment A to all the other molecules that form the stack. For the stacks made of fully distorted fragments, the relative phase of the molecular orbitals on the different fragments is less obvious as there is no one-to-one correspondence between the distorted fragments. In any case, the phase of the fragment orbitals (and the CASSCF wave functions) is irrelevant for the CT-enhanced electronic couplings as numerically illustrated in Section S2 in the Supporting Information.

To account for the dynamic electron correlation, the CASSCF state energies are corrected with the corresponding dynamic electron correlation at the CASPT2 level by applying a shift to the diagonal elements of the NOCI matrix.^73^

### 2.5 Multifragment Full Hamiltonian

Herein we present a novel approach that allows for an advanced analysis of the electronic features of the full stack of molecules. Rather than performing a computationally intractable calculation on the entire stack, the NOCI-F results obtained for the different dimers and/or trimers are used to construct the Hamiltonian and overlap matrices of the full stack. Solving the resulting general eigenvalue problem gives information about its electronic properties. For example, this approach allows to study the delocalization of the different many-electron states over the stack.

To describe the whole stack, we define a set of MEBFs as many-body basis of the multifragment full Hamiltonian (MFH) approach. Each MEBF corresponds to a well-defined configuration of excitations or charge distributions across the stack. In line with the above-described MEBFs for dimers and trimers, six different classes can be distinguished in the case of singlet spin coupling, albeit in the full stack there are many more MEBFs in each class. Table 1 summarizes the MEBFs that span the 55 × 55 Hamiltonian for a 9-molecule stack and total singlet spin. In addition, triplet, hole and electron diffusion processes are studied with a Hamiltonian spanned by the local triplet excited states combined with triplet-coupled CT states, and two Hamiltonians arising from the cationic and anionic states, respectively. Figure 5 illustrates the shorthand notation for the AB dimer.

**Table 1:** Definition of the Six Different Classes of MEBFs That Span the 55 × 55 Hamiltonian of a 9-Molecule Stack with Singlet Spin Coupling^a^

full label	short label	character	number of MEBFs
*S*_0_ *S*_0_ *S*_0_ *S*_0_ *S*_0_ *S*_0_ *S*_0_ *S*_0_ *S*_0_	GS	ground state	1
*S*_1_ *S*_0_ *S*_0_ *S*_0_ *S*_0_ *S*_0_ *S*_0_ *S*_0_ *S*_0_	A	local excited singlet	9
*T*_1_ *T*_1_ *S*_0_ *S*_0_ *S*_0_ *S*_0_ *S*_0_ *S*_0_ *S*_0_	AB	coupled double triplet	8
*T*_1_ *S*_0_ *T*_1_ *S*_0_ *S*_0_ *S*_0_ *S*_0_ *S*_0_ *S*_0_	AC	separated double triplet	7
*D* ^+^ *D* ^–^ *S*_0_ *S*_0_ *S*_0_ *S*_0_ *S*_0_ *S*_0_ *S*_0_	A+B–	nearest neighbor CT	16
*D* ^+^ *S*_0_ *D* ^–^ *S*_0_ *S*_0_ *S*_0_ *S*_0_ *S*_0_ *S*_0_	A+C–	next nearest neighbor CT	14

a One example is given for each class, a full overview can be found in Table S9 of the Supporting Information.

**Figure 5:**
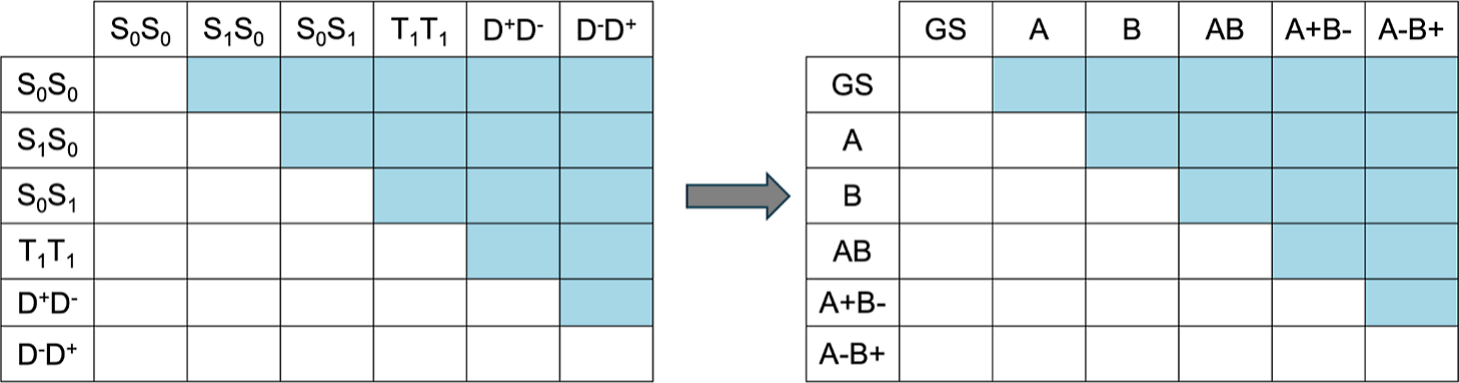
Shorthand notation used for the Hamiltonian and overlap matrices for dimer AB. The unique elements necessary to obtain all the couplings appear as empty cells.

Each element of the Hamiltonian and overlap matrices is constructed from precomputed data, that is, from the dimer and/or trimer NOCI-F calculations. However, one must address the fact that some matrix element types appear in more than one ensemble and, for stacks extracted from the MD trajectory, these matrix elements are not strictly the same. For example, the local excited singlet on molecule C of the stack is computed in the ABC, BCD and CDE trimers. The differently distorted members of these trimers lead to slightly different relative energies of the *S*_1_ state on molecule C in each of these trimers. Figure 6 represents the 13 × 13 Hamiltonian (or overlap) matrix of the BCD trimer, indicating which elements are also calculated in the ABC (green), CDE (red) and DEF (blue) trimers.

**Figure 6:**
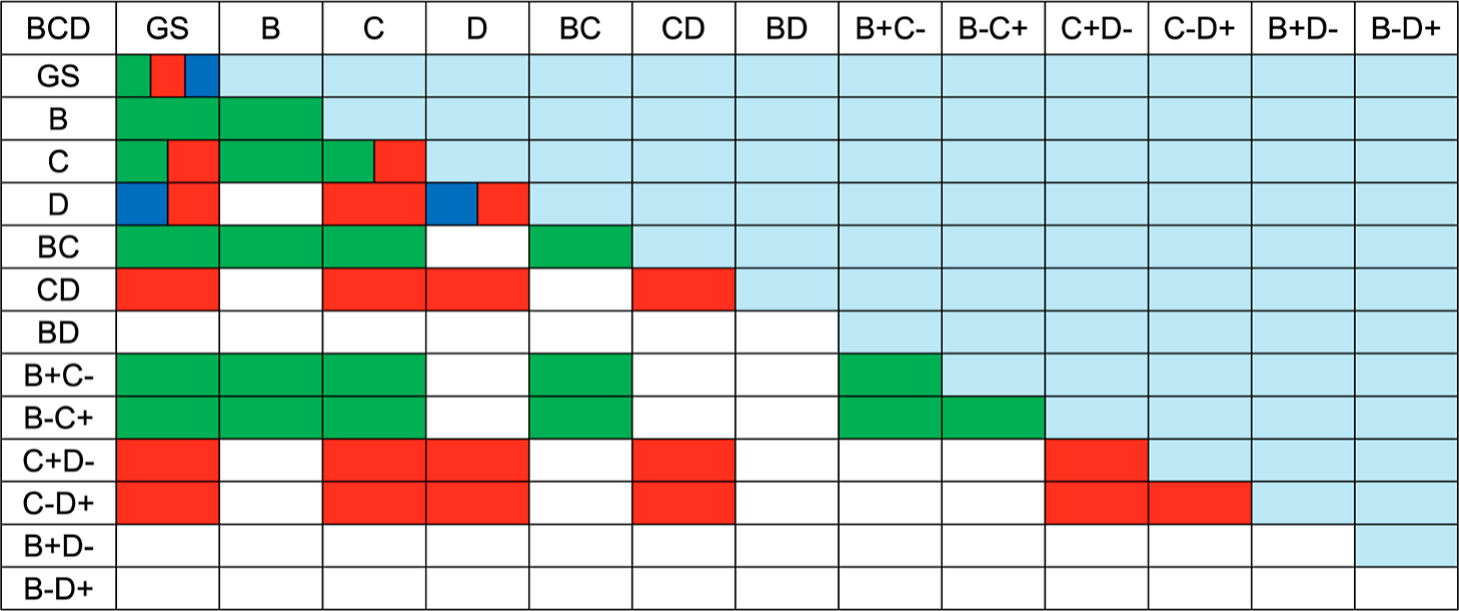
Matrix elements common to BCD and other trimers: ABC (green), CDE (red) and DEF (dark blue). White cells correspond to elements that only appear in calculations on the BCD trimer.

In order to tackle this, we employ an averaging procedure to ensure internal consistency (see Tables S11–S18 in Supporting Information). Thus, the matrix elements of the full stack are calculated using the following principles (see Section S7 in the Supporting Information):

- Diagonal (Energies): the average of the GS energies calculated for the different fragments defines the ⟨GS|Ĥ|GS⟩ element. The energies of the other MEBFs are calculated by averaging the relative energies of each occurrence and adding these values to the average energy of the GS MEBF.
- Off-Diagonal: the matrix elements between two different MEBFs are directly taken from the previous dimer or trimer calculations and if an element appears in multiple overlapping fragments (e.g., in dimers AB and BC), the final matrix element is assigned as the average of all such appearances.

This procedure is implemented in a Python script, included in the latest GronOR release, which is available at GitLab (gitlab.com/gronor). This enables the automated construction of the full-stack Hamiltonian and overlap matrices from a finite and computationally tractable set of fragment calculations.

Once the Hamiltonian and overlap matrices are assembled, the total wave function is obtained by solving the generalized eigenvalue equation **HC = SCE**, where **H** is the Hamiltonian matrix, **C** are the NOCI-F expansion coefficients, **S** is the overlap matrix, and **E** are the energies of the resulting states of the full stack.

## 3 Results and Discussion

After introducing the basic features of the INDO unit, Section 3.2 presents the results for ordered dimer models shown in Figure 3. In Section 3.3 we turn our attention to dimers and trimers with distorted arrangements generated by MD. Finally, the results obtained for larger disordered or ordered assemblies are discussed in Section 3.4, stressing their differences.

### 3.1 Electronic Characteristics of the Indolonaphthyridine Unit

Figure 7 shows the active orbitals of the *S*_0_ state for the INDO molecule in the optimized geometry. As recently shown by some of us,^37^ the size of the active space is not critical, provided it is *large enough*. For molecules with a π system of this size, CAS­(8,8) has been shown to include the most important static electron correlation effects and relative energies that are similar to those obtained from larger active spaces. The active orbitals of the other electronic states maintain the overall shape of the orbitals shown in Figure 7 for the ground state. With the computational setup described above, the relevant electronic states have CASPT2 energies (referred to the ground state, *S*_0_) of 1.83 eV for *S*_1_ and 0.81 eV for *T*_1_. The ionic states *D* ^–^ and *D* ^+^ are found at energies of 7.10 eV and −1.90 eV, respectively. If no dynamic electron correlation is considered, these relative energies are 4.16 eV, 2.25 eV, 7.27 eV and −0.53 eV, respectively, highlighting the importance of taking into account the short-range electron–electron correlation for a correct description of electronic phenomena.

**Figure 7:**
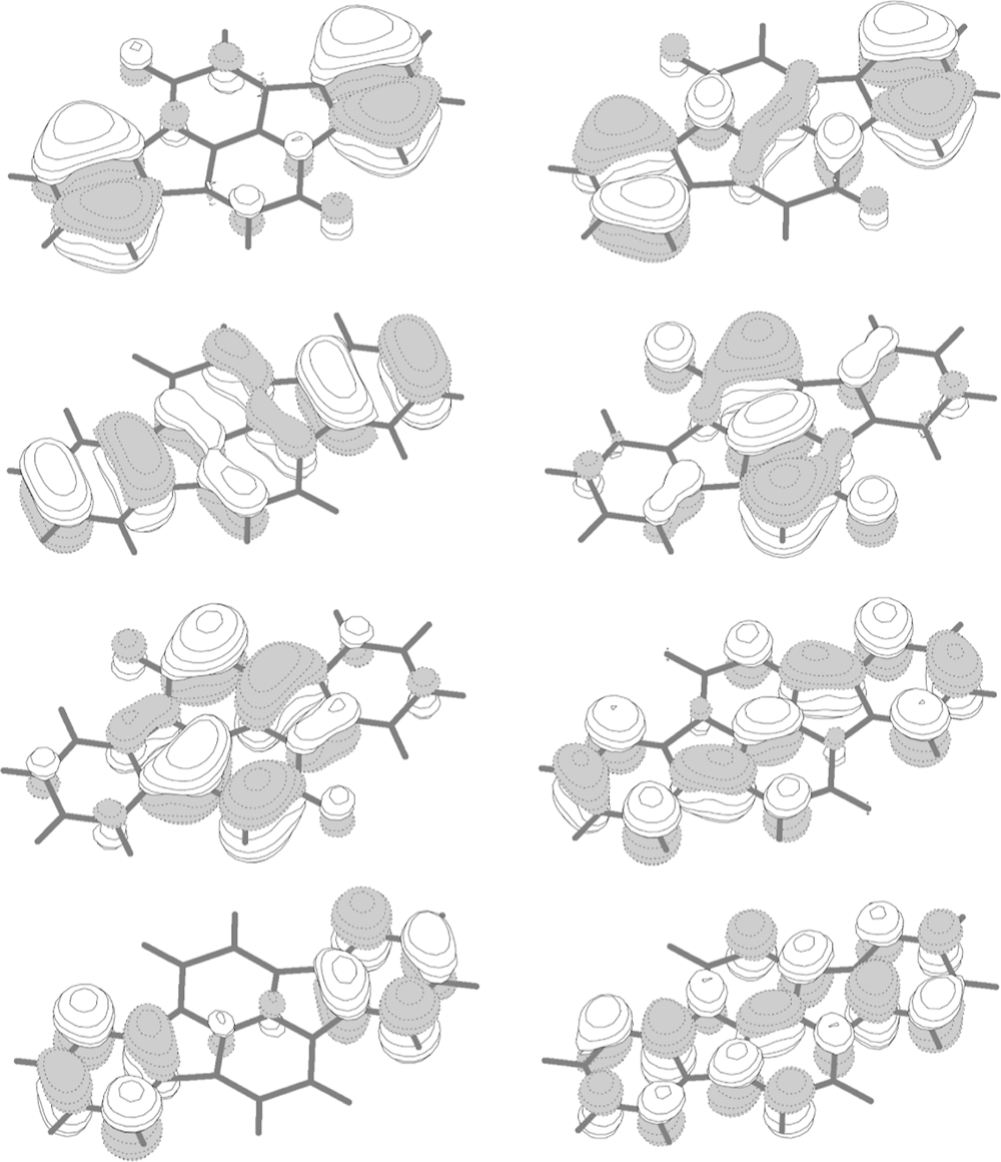
Molecular orbitals included in the CAS­(8,8) for the *S*_0_ state of indolonaphthyridine.

### 3.2 Ordered INDO Dimer Models

This model allows us to have full control on the arrangement and geometries of the system and its fragments. This is an idealized scenario in which maximum order governs the outcome of the NOCI-F calculations.

#### 3.2.1 Electronic Couplings

##### 3.2.1.1 Singlet Fission

The main results are summarized in Figure 8 for slip-I and slip-II models. All the results refer to CT-enhanced couplings, and dynamic electron correlation corrected energies for the states involved. It stands out that on-top dimers show negligible SF couplings for any rotation angle, showing that this type of structural arrangement completely disfavors SF, as shown for a similar dimer model by Singh et al.^75^ On the other hand, slip models feature sizable SF couplings, with a peak of 13 meV for model slip-I at a rotation of about 15°. Similarly, model slip-II shows a maximum close to 20°. We observe that the SF coupling strongly varies as the rotation angle increases, with minima in the range 40–45° and maxima at 10–20°. The average SF couplings for the series of rotation angles in models slip-I and slip-II are 5.7 and 8.3 meV, respectively.

**Figure 8:**
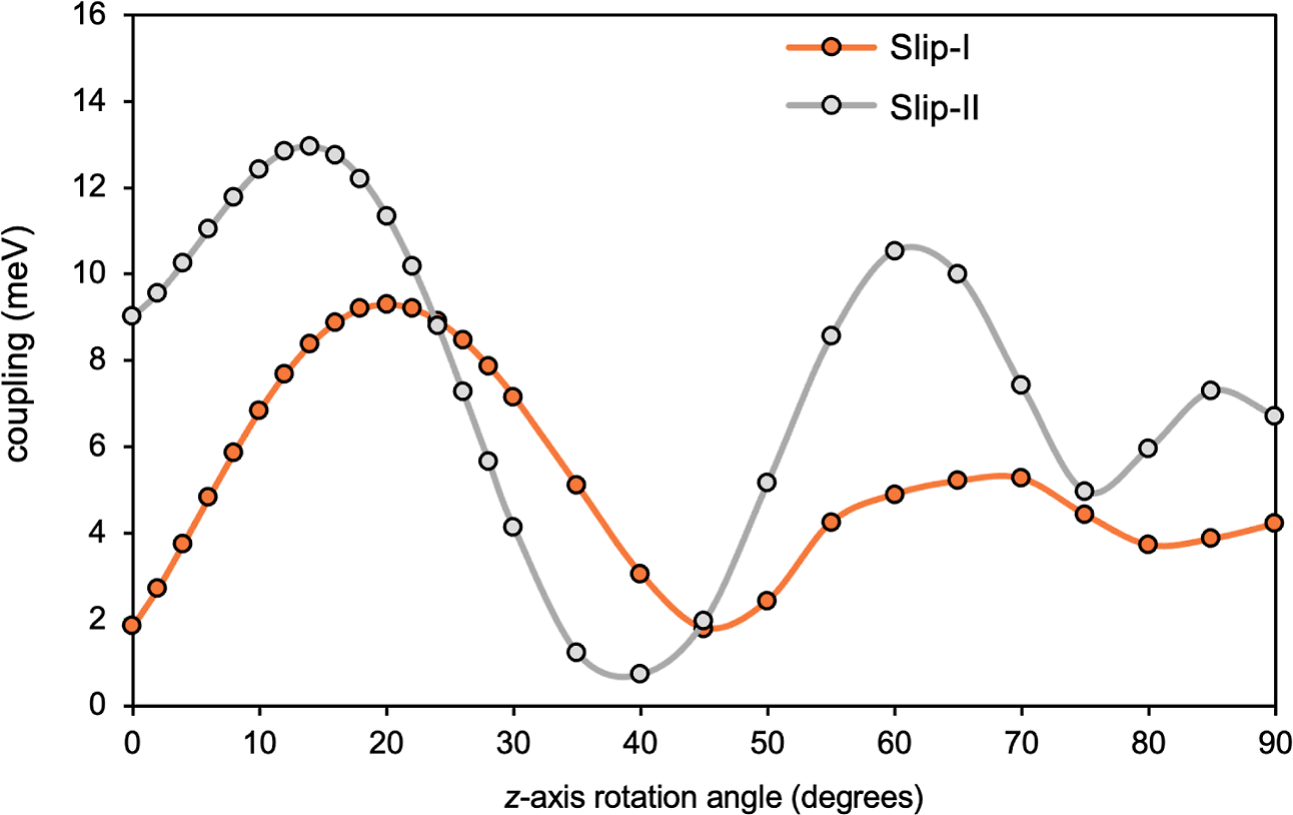
SF couplings (in meV) vs interplanar *z*-axis rotation angles (in degrees), computed for slip-I (orange dots) and slip-II (gray dots) dimer models.

The difference between direct and CT-enhanced couplings is worth being commented. To illustrate this, Figure S2 compares the coupling values obtained in either case for the slip-II model at rotation angles 10°, 20°, 30° and 70°. The corresponding direct couplings are 3.5 meV, 3.6, 1.8, and 1.7 meV, approximately 50 to 75% smaller than the CT-enhanced couplings. This comparison between both approaches reveals the importance of considering charge transfer states in SF.

##### 3.2.1.2 Excited Singlet Diffusion

Diffusion of an excited singlet (*S*_1_ *S*_0_ → *S*_0_ *S*_1_) in ordered dimers results in large electronic couplings in general, with a notable difference between on-top and slip models (Figure 9, top). The computed values for on-top configurations between 0 and 35° lie within the 450–190 meV range, smoothly decreasing to zero for perpendicular fragments. The maximum coupling is obtained for on-top parallel fragments for which a delocalized excited singlet is also a valid description. In comparison, the models of slip fragments present smaller singlet diffusion electronic couplings. The maximum values are close to 100 meV for parallel and small interfragment angles (0–40°). This coupling reaches 0 meV for 75° rotation.

**Figure 9:**
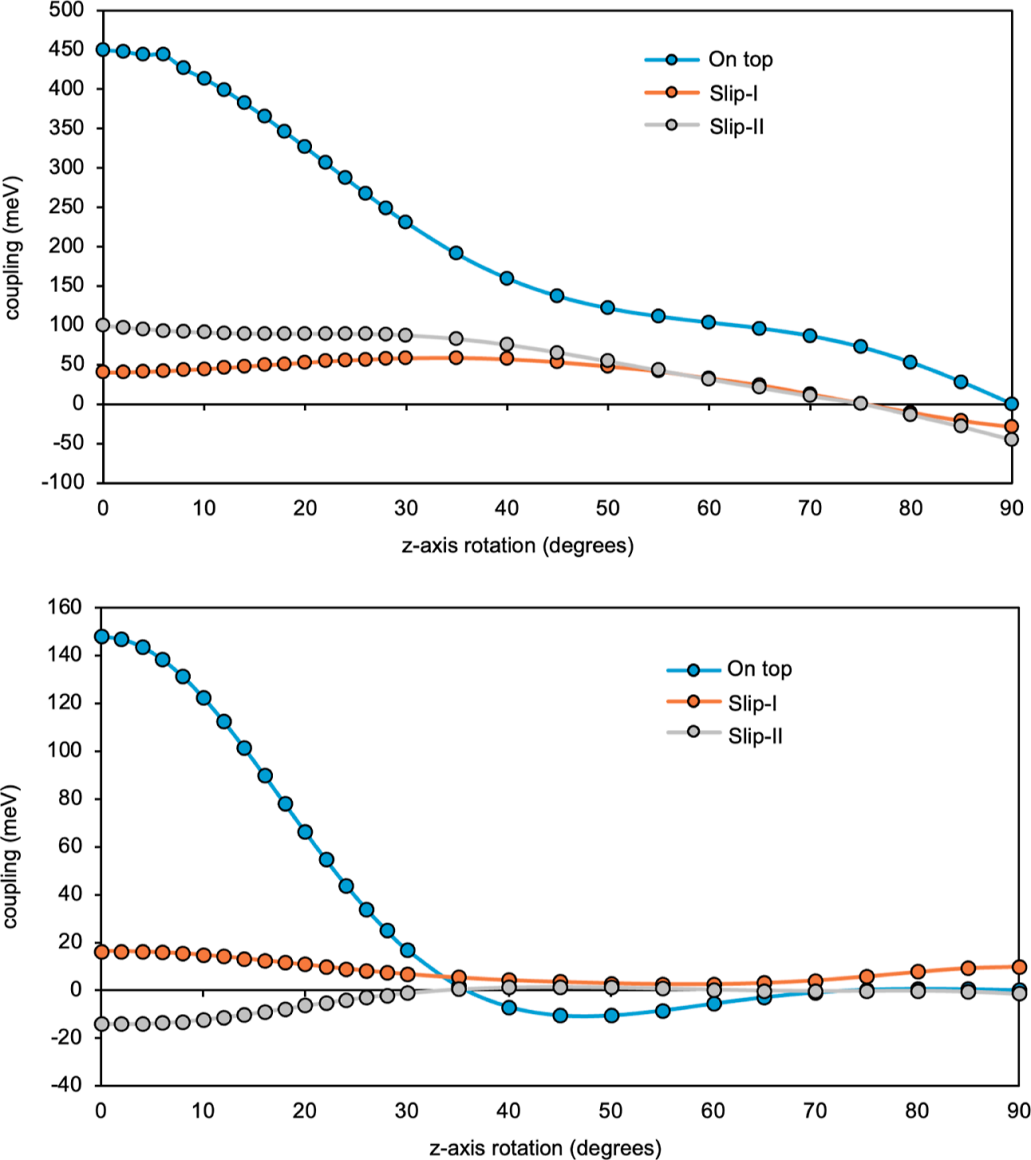
Singlet diffusion (top) and triplet diffusion (bottom) electronic couplings (in meV) vs interplanar *z*-axis rotation angles (in degrees), computed for on-top (blue dots), slip-I (orange dots) and slip-II (gray dots) models of dimers of planar INDO units. The vertical scales are considerably different.

##### 3.2.1.3 Triplet Diffusion

The behavior of the *T*_1_ diffusion couplings is qualitatively similar, although the values are overall smaller than for *S*_1_ diffusion. Again, on-top configurations present the largest couplings in the range of 0–30° interfragment rotations, and a maximum coupling of 148 meV for parallel units (see Figure 9, bottom). A dramatic decrease of the couplings occurs upon increasing *z*-axis rotations until reaching zero coupling at 35°. For larger rotations, the couplings remain negative between 0 and −10 meV. Slip models I and II present a range of *T*_1_ diffusion couplings between 0 and 16 meV in absolute value, suggesting that slippage of the units goes against this electronic energy transfer, especially for moderate to large interfragment mutual rotations. Note that the rate of these transfer processes depends on the square of the coupling, following Fermi’s golden rule. Hence, the sign of the coupling is not relevant in this aspect.

##### 3.2.1.4 Hole and Electron Diffusion

The last electronic processes studied in this section are hole (*D* ^+^ *S*_0_ → *S*_0_ *D* ^+^) and electron (*D* ^–^ *S*_0_ → *S*_0_ *D* ^–^) diffusion, also related to materials acting as charge carriers in the area of solar light harvesting. The range of coupling values computed is wide, with maxima of 360 and 450 meV for hole and electron diffusion, respectively, in on-top parallel arrangements (see Figure 10). Slip-I and –II models present smaller values in general, as earlier observed in the singlet and triplet diffusion. These reach maximum values for hole diffusion of ca. 70 meV (slip-I model) and 150 meV (slip-II model), and for electron diffusion of ca. 185 meV (slip-I model) and 170 meV (slip-II model). The variation of coupling values as the fragments are mutually rotated can lead to negative couplings, such as the hole diffusion for the slip-II model, or the electron diffusion for on-top fragments between 35 and 90° rotations. As mentioned above, the sign does not affect the rate of the transfer process.

**Figure 10:**
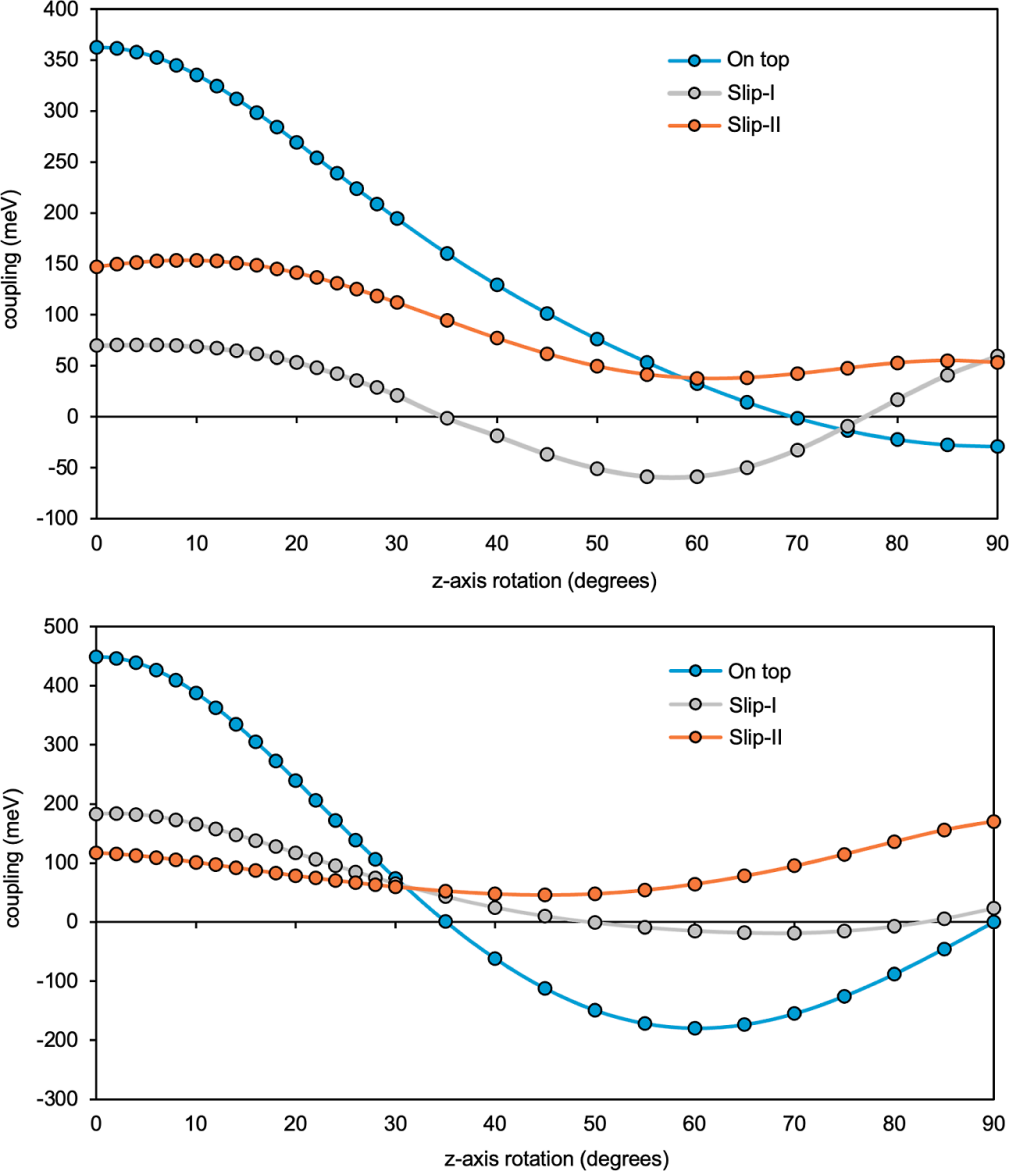
Hole diffusion (top) and electron diffusion (bottom) electronic couplings (in meV) vs interplanar *z*-axis rotation angles (in degrees), computed for on-top (blue dots), slip-I (orange dots) and slip-II (gray dots) models of dimers of planar INDO units.

This section has demonstrated that planar parallel INDO fragments present large electronic couplings in general, especially for certain interfragment rotations. This model provides relevant information about the potential of stacked organic molecules as energy/charge carriers. SF electronic couplings present modest values, and the fact that on-top INDO fragments cannot show SF reveals that disorder in prospective real material can be a differentiating factor for their applicability.

### 3.3 Distorted INDO Stack

#### 3.3.1 Structural Features of the Distorted Model

The results presented herein correspond to dimers and trimers taken from Stacks 5 and 6 model, as described in Section 2.3.2. The selected arrangements constitute realistic scenarios for a material since they capture the thermal effects on electronic couplings and their magnitudes, as other computational studies have shown.^76^ Figure 4 shows Stack 5, obtained with DMF as solvent. Fragment labeling is used for later discussion. The stack presents internal disorder of diverse nature, namely, noncoplanar neighboring units, irregular plane-to-plane distances and multidirectional rotations. In a detailed computational MD study, Cantina et al. have recently reported similar features for PDI molecules in aqueous medium.^77^ In addition, each fragment is internally distorted. It must be said that this is a representative snapshot of the structural features of the stack, since inspection revealed that this type of arrangement is maintained for the full length of the simulation.

Table 2 lists some structural parameters for dimers taken from the stack shown in Figure 4. It can be seen that the distances between centers of mass vary from 3.64 to 4.43 Å. These values show that all interplanar distances are close to 3.5 Å, since for the regular models discussed in Section 3.2 the distances between centers of mass are 3.50 and 4.47 Å for on-top and slip models, respectively, and 3.5 Å is the fixed interplanar distance. The diversity of these values suggests slippage disorder within the range that was studied in the regular stacks. The angle between the fragments’ normal vectors (α) and between the long in-plane vectors (β) also varies considerably, indicating no coplanarity and mutual interfragment rotation. Finally, the rightmost column of Table 2 is a metric accounting for the loss of planarity of each fragment (where full planarity corresponds to PI = 0, see Section S6 in the Supporting Information for details). Fragments A and F are the most and the least planar units in the series, respectively. Although not shown here, attempts at correlating these parameters, alone or combined, with the values obtained for the electronic couplings discussed below gave no clear trends. This does not mean that structural and electronic features have no connection but, instead, that it is complex and multifaceted.

**Table 2:** Structural Parameters of the Dimers Extracted From Stack 5

*XY*	Δ*R* ^a^	α^b^	β^c^	PI^d^ *X*/*Y*
AB	3.98	8.9	11.1	0.642/0.800
BC	3.70	2.3	20.2	0.800/1.038
CD	3.64	4.1	63.2	1.038/0.758
DE	4.43	8.6	–21.1	0.758/0.908
EF	3.74	3.1	45.6	0.908/1.811
FG	3.92	6.9	–27.7	1.811/0.711
GH	4.03	3.5	4.2	0.711/0.901

a Distance (in Å) between the centers of mass of fragments *X* and *Y*.

b Angle (in degrees) between the vectors normal to the molecular planes of fragments *X* and *Y*.

c Angle (in degrees) between the long molecular axes of fragments *X* and *Y*.

d Planarity index: deviation from full planarity for each fragment.

#### 3.3.2 Electronic Couplings in Fully Distorted and in Disordered Planar Dimers

This subsection shows the changes experienced in electronic couplings when the model incorporates the disorder and distortion associated with thermal energy. By taking a MD-generated geometry we represent a realistic instant of an INDO stack to which we apply the same procedures described in Subsection [Sec sec3.2.1] to calculate electronic couplings. We combine the discussion of the fully distorted Stack 5 with the one retaining the original MD interfragment disorder but with planar fragments (Stack 6). A similar approach was applied in the study of SF in pure and doped pentacene crystals.^37^

##### 3.3.2.1 Singlet Fission

Allowing the fragments to gain disorder significantly increases the SF couplings. In Table 3, top, the values obtained with this model range from 1.9 to 35.0 meV, with an average value of 12 meV. For comparison, for the regular on-top and slip models, electronic couplings ranging from 0 to 13 meV were obtained. For the slip-I and slip-II models, the average SF couplings were computed to be 5.7 and 8.3 meV, respectively. The new couplings evidence that SF is favored by disorder.

**Table 3:** Nearest-Neighbor Electronic Couplings^a^ Obtained with the INDO Dimer Models Based on MD Simulations

model	process	AB	BC	CD	DE	EF	FG	GH
Stack 5^b^	SF	1.9	35.0	13.0	2.3	20.6	8.9	5.3
	*S*_1_	1.6	95.9	4.6	34.4	74.9	133.6	94.2
	*T*_1_	0.7	0.4	5.4	0.4	2.2	1.2	1.1
	h^+^	117.6	66.2	106.1	16.6	97.5	99.6	42.7
	e^–^	30.1	73.5	69.5	11.9	2.0	31.4	33.4
Stack 6^b^	SF	3.4	69.9	26.9	2.5	21.5	9.6	9.2
	*S*_1_	172.3	367.3	63.3	154.4	123.1	186.4	198.5
	*T*_1_	0.3	2.3	1.0	2.0	1.1	0.2	0.9
	h^+^	84.6	317.2	87.2	30.8	91.8	114.5	6.0
	e^–^	8.7	247.6	37.0	64.0	1.2	40.4	84.5

a Values in meV.

b See text for a detailed description of the model.

When the values obtained with the thermally distorted and disordered stack are recalculated with planar fragments (listed in Table 3, bottom), the new SF couplings present modest changes in most cases. Dimer BC presents the largest absolute variation, with a SF coupling that is 35 meV larger. It stands out that all the couplings increase if planar fragments are considered, that is, intrafragment distortion tends to decrease the effectiveness of the coupling. In consequence, we infer that the combined effect of loss of coplanarity by slippage and mutual rotation of neighboring fragments must be the main facts in favor of large SF couplings.

##### 3.3.2.2 Excited Singlet Diffusion

The case of *S*_1_ diffusion also presents important differences with respect to the regular models discussed in the previous section. First, the values calculated with the fully disordered model are considerably smaller than the values reported above for on-top ordered arrangements with small *z*-axis rotations (200–450 meV) as illustrated in Figure 9, top. The average coupling for the disordered stack is 63 meV. However, these values are of the same order as the ones obtained for slip-I and slip-II models, which reached a maximum of ca. 100 meV. In particular, the fully disordered model presents one value of 134 meV and three other above 70 meV. The smallest couplings, of ca. 1–5 meV, correspond to dimers AB and CD. Seen as a whole, the range of values is large.

Considering the model with planar fragments in the positions and orientations of the fully distorted case, a dramatic and generalized increase in the *S*_1_ diffusion couplings is obtained. See Table 3 for the comparison, and Figure S3 for the graphical view of the variations, some of them larger than 100 meV, indicating that planarity strongly favors exciton diffusion.

##### 3.3.2.3 Triplet Diffusion

In contrast to singlet diffusion, *T*_1_ exciton diffusion presents small couplings. For the fully distorted dimer model, the values are limited to a maximum of 5.4 meV (dimer CD), with an average of 1.6 meV in the series. By replacing the distorted units by planar INDO fragments, small changes are observed since 1.7 meV is the new central value, and the largest one is 6.8 meV (see Table 3).

If we compare these values with the ones obtained for regular ordered dimers, we confirm that triplet diffusion presents modest values for every model considered. The exception is the case of planar on-top dimers, which can reach values larger than 100 meV for small relative fragment rotations (see Figure 9, bottom).

##### 3.3.2.4 Hole and Electron Diffusion

Along with singlet exciton diffusion, these phenomena display the largest couplings. Taking the reference values shown in Figure 10 for ordered planar dimers, with maxima reaching hundreds of meV in on-top conformations, the corresponding couplings for the fully distorted model are systematically smaller, limited to a maximum of 117.6 meV (hole diffusion) and 73.5 meV (electron diffusion). The corresponding average values for the calculated dimers are 78 and 36 meV. It is interesting to point out that dimer EF presents, by far, the smallest electron diffusion coupling paramater (2.0 meV). This dimer presents an interfragment rotation angle of 45°, which approximately matches the angles corresponding to minima in the curves in Figure 10, bottom. To confirm this observation, if we remove the intrafragment disorder factor by taking the same dimer with planar fragments, the coupling remains very small (1.2 meV).

Reduction of disorder by introducing planar fragments has important effects on the electronic couplings, as can be seen in Figure S3. Large variations occur, which increase or decrease the coupling in an apparently unpredictable manner.

#### 3.3.3 Comparison of Electronic Couplings in Distorted Dimers and Trimers

The NOCI-F results obtained for the trimers taken from the same MD stack structure are compared with those presented above for dimers in Table 3, restricting the present analysis to fully distorted fragments only. Tables S1–S8 compare the electronic couplings for all the INDO dimers and trimers taken from Stacks 5 and 6. NOCI-F calculations on trimers require significantly more node-hours, but they give access to phenomena that cannot be captured within the dimer model,^78,79^ for example second-neighbor couplings such as *S*_1_ *S*_0_ *S*_0_ → *S*_0_ *S*_0_ *S*_1_ and triplet separation, *S*_0_ *T*_1_ *T*_1_/*T*_1_ *T*_1_ *S*_0_ → *T*_1_ *S*_0_ *T*_1_.

It stands out that the large majority of values computed with either model are very similar. Only a few cases present significant differences, and are mostly attributed to a more complete description of the CT effects. Whereas only two CT states can be defined in the dimer (*D* ^+^ *D* ^–^ and *D* ^–^ *D* ^+^), the trimer model contains six CT states (*D* ^+^ *D* ^–^ *S*_0_, *D* ^–^ *D* ^+^ *S*_0_, *S*_0_ *D* ^+^ *D* ^–^, *S*_0_ *D* ^–^ *D* ^+^, *D* ^+^ *S*_0_ *D* ^–^, *D* ^–^ *S*_0_ *D* ^+^). In most cases, the interaction of the extra CT states with the MEBFs of interest is small and the electronic couplings extracted from dimers and trimers are virtually the same. However, when the extra CT contribute significantly to the “CT-dressed” MEBFs, the trimer couplings can deviate from those extracted in the dimer.

The general bottom line for this part is that the trimer model can give a refinement to the values obtained with dimers, but at a considerably larger cost depending on the fragment size. This said, the qualitative and quantitative description of the phenomena obtained with the more affordable dimer model are in agreement with the trimer model in about 95% of the cases analyzed in this work.

### 3.4 Full NOCI-F Wave Function: From Dimers/Trimers to Stack

In this section, we present the results aimed at describing the physics of full stacks of INDO molecules, instead of focusing on the interactions among two or three molecules in a stack. Following the MFH procedure outlined in Section 2.5, a stack made of *N* INDO molecules and involving five electronic states per molecule, requires (*N* – 2) NOCI-F calculations on trimers and leads to a (7*N* – 8) × (7*N* – 8) Hamiltonian (see Table S9 for the 55 elements of a 9-unit stack). Increasing the number of molecules in the stack does not dramatically increase the computational cost, as including an extra molecule only requires one extra set of CASSCF calculations to calculate the fragment wave functions and one additional NOCI-F trimer calculation. The dimension of the final Hamiltonian and overlap matrices remains small as it only increases with 7 extra MEBFs. This indicates that treating larger stacks does not increase the computational cost substantially, but rather keeps it linear.

The construction of the full Hamiltonian based on ensembles with three molecules limits the interactions to first- and second-neighbor interactions. The interactions beyond that are not considered, although molecules that are further apart indirectly interact through the diagonalization of the Hamiltonian of the whole system. If interactions beyond second-neighbors are expected to play a role, the full Hamiltonian should be constructed from NOCI-F calculations on tetramers.

#### 3.4.1 Stacks 1 and 2: Ordered Planar Fragments

The staircase ordered stack contains 10 molecules (Stack 1, see Figure S4), resulting in 62 × 62 NOCI-F Hamiltonian and overlap matrices. The values of the matrix elements are taken from the NOCI-F calculations reported in Section 3.2.1. Table 4 lists the relative energies and MEBF character of the NOCI wave functions (WF or Ψ_*i*_ hereafter) after applying the MFH approach. As required for the SF process, most *S*_1_-dominated states are slightly higher in energy than the *T*_1_ *T*_1_ and *T*_1_···*T*_1_ manifolds. Notably, there are four NOCI states (2–3 and 11–12) that show equal contributions from the singlet exciton and the double triplet MEBFs, which could enhance the probabilities for SF. The significantly larger electronic coupling for singlet diffusion compared to the corresponding one for *T*_1_ *T*_1_ diffusion makes that the *S*_1_ manifold extends over an energy range of approximately 0.3 eV, while the double triplet states are virtually degenerate. The higher-energy WFs are predominantly composed of the charge-transfer NN and NNN MEBFs. The full information of the NOCI WFs can be found in the Supporting Information in Table S10.

**Table 4:** Relative Energies (in eV) and Character (in Terms of the MEBFs) of the NOCI WFs of Stack 1

NOCI WF	Δ*E*	character
1	0.00	GS
2–3	1.68	*S*_1_ (50%), *T*_1_ *T*_1_ (50%)
4–10	1.70	*T*_1_ *T*_1_ (>90%)
11–12	1.72	*S*_1_ (50%), *T*_1_ *T*_1_ (50%)
13	1.74	*S*_1_ (66%), *T*_1_ *T*_1_ (13%), *T*_1_...*T*_1_ (20%)
14–21	1.74	*T*_1_...*T*_1_ (>90%)
22–28	1.77–2.06	*S*_1_ (>85%)
29–46	2.81–2.98	CT NN
47–62	3.83–3.89	CT NNN

For the similar Stack 2, Figure 11a displays the Gallup–Norbeck^80,81^ (GN) weights of the *S*_1_ MEBFs in three of the NOCI-F WFs with strong singlet exciton character (total weights of 0.94, 0.92 and 0.87, respectively), while the remaining contributions primarily originate from CT MEBFs exhibiting a similar distribution pattern. The fact that all three WFs (and all other *S*_1_-dominated WFs) have contributions from all molecules in the stack demonstrates the fully delocalized character of the singlet exciton. A detailed inspection of the weights of each individual molecule reveals that the two units on the extremes of the stack carry on average a slightly larger weight than the molecules in the rest of the stack. This is not unexpected for a finite stack of the type that was reported in the experimental studies,^39,41^ but if the system under study is an (quasi-)­infinite regular stack, such asymmetry can be mitigated easily within the MFH approach by adding the elements to the Hamiltonian and overlap matrices that connect the two units at the extremes of the stack. In this way a true “periodic” character is obtained. Additional data on Stack 2 are presented in Tables S13–S15.

**Figure 11:**
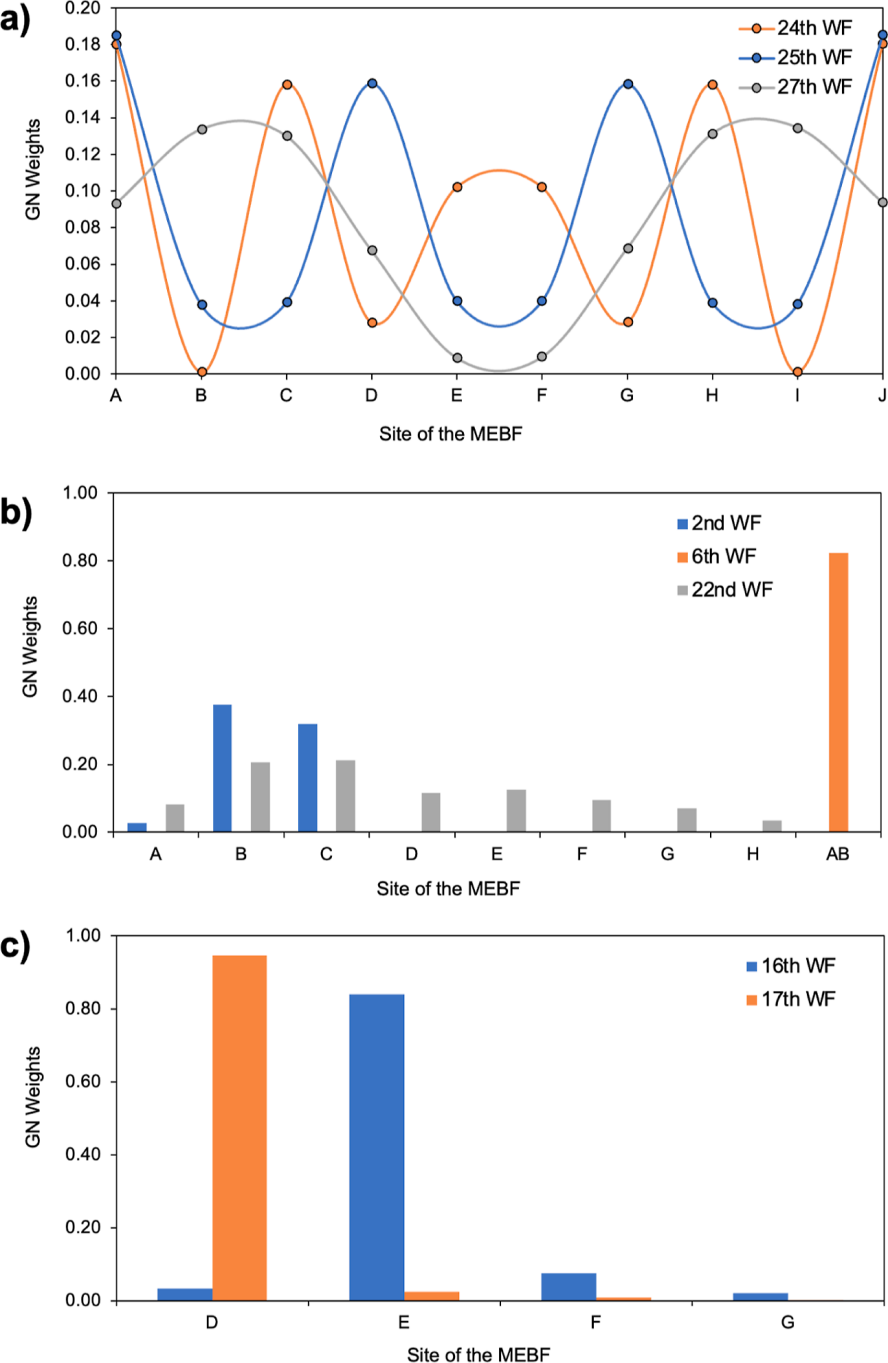
(a) Gallup–Norbeck weights of all the delocalized *S*_1_ MEBFs in three of the lowest energy MFH NOCI-F wave functions for the ordered planar Stack 2. (b) Example of all the possibilities in terms of wave function localization/delocalization for the disordered planar Stack 6. (c) Two completely localized wave functions on two different *S*_1_ MEBFs in the disordered distorted Stack 5.

Tables S11 and S12 compare electronic couplings (in rows) computed with standard NOCI-F on three trimers with those obtained with the MFH approach on the whole stack. It stands out that for the ordered Stack 2, both methods are completely coincident, but the distorted Stack 3 presents some differences. These are attributed to the very definition of the MFH approach, which takes into account the extra effects originating in the presence of other fragments for building the MEBFs.

#### 3.4.2 Stack 6: Disordered Planar Fragments

In the stack composed of eight spatially disordered planar INDO molecules (discussed in Section 3.3.2), the full Hamiltonian model captures a wide range of localization/delocalization regimes within the NOCI-F WFs. As illustrated in Figure 11b, the GN weights for three representative WF (second, sixth and 22nd) demonstrate distinct behaviors corresponding to different excitonic characters. The sixth WF (orange) of dominantly *T_1_T*_1_ character exhibits complete localization on molecules A and B, as evidenced by the total GN weight being entirely concentrated on the AB MEBF. In contrast, the 22nd WF (gray) of mostly *S*_1_ character displays a delocalized character, with GN weights spread relatively evenly over multiple sites from A to H, similar to the case of the ordered and planar stack. Finally, the second WF (blue) also displays *S*_1_ character since it is localized primarily on the B and C MEBFs (see Table S9). These three cases highlight how disorder disrupts the delocalization observed in Stack 1 and introduces heterogeneity in the WF character. Disordered Stack 6 system therefore exemplifies the full spectrum of localization possibilities, from complete to partial localization, and to complete delocalization across the stack.

#### 3.4.3 Stack 5: Fully Distorted Fragments

In the disordered and distorted stack composed of eight INDO molecules (Stack 5), the MFH approach reveals strongly localized WFs for almost all states. This is illustrated for Ψ_10_ and Ψ_17_ in Figure 11c. The GN weights indicate full localization on molecules E and D for Ψ_16_ and Ψ_17_, respectively. A slightly different behavior was observed here for Ψ_5_ and Ψ_6_, which correspond to a superposition of two spatially separated *T*_1_ *T*_1_ MEBFs (CD and HI) as shown by the GN weights: the fifth WF consists of 20% CD and 80% HI and the sixth WF consists of 80% CD and 20% HI. This superposition is caused by the accidental (near-)­degeneracy in energy between these two MEBFs and the non-negligible interaction of both with GS, leading to mixed-state character of the WF despite being far apart. Finally, these results demonstrate that the disordered and distorted model supports complete localization of the WFs, highlighting the significant role of both disorder and structural distortion in modulating MEBF behavior across stacked molecular systems. Additional data on Stack 5 are presented in Tables S16–S18.

#### 3.4.4 Couplings beyond the Trimer Model

Tables S19–S30 collect all the electronic couplings computed with the MFH approach for all the stacks presented in this work. Another interesting advantage of the MFH is its ability to include CT states in the process of determining the CT-enhanced couplings that go beyond the trimer. For example, the SF coupling for B with BC can be dressed with B+C– and B–C+ in a dimer model. The BCD trimer model allows to add four more CT states (C+D–, C–D+, B+D– and B–D+). Applying the MFH approach one could extend this first with CT states involving unit A to obtain a more balanced CT dressing than in the trimer. Eventually, one can add CT states involving units that are further away, although these are not expected to contribute in a significant manner.

Another coupling of interest is the so-called direct SF coupling, referring to the interaction between an *S*_1_ MEBF and a spatially separated *T*_1_···*T*_1_ MEBF (see Table S30 in the Supporting Information). Although this coupling is zero when CT states are not taken into account, it can become significant when both are dressed with those,^74^ with values reported of a few meV.^79^ This effect is illustrated in Table 5 for a local excited singlet on G directly evolving into a *T*_1_···*T*_1_ state on F and H. The table shows that the direct coupling between G and FH is negligible (0.001 meV). When G and FH are dressed with either NN or NNN CT MEBFs, the resulting coupling remains small (∼0.01 meV). However, the inclusion of both the NN and NNN CT MEBFs of the FGH trimer (i.e., F+G–, F–G+, G+H–, G–H+, F+H–, and F–H+) yields a coupling of −0.4 meV. Figure 12 illustrates the mechanism behind this enhancement. It shows that G interacts strongly with the NN CT states, while FH has a sizable interaction with NNN CT. The key factor is that NN interacts strongly with NNN, completing the pathway from G to FH through strong interactions. Any further inclusion of CT states in the dressing procedure does not enhance the coupling. The final estimate of the coupling is not as large as the one reported in ref 79, but the here-described procedure enables a straightforward evaluation for future studies to establish whether this direct formation of the *T*_1_···*T*_1_ state also occurs in other materials.

**Table 5:** Electronic Coupling for Direct SF (*S*_0_ *S*_1_ *S*_0_ → *T*_1_ *S*_0_ *T*_1_) in Trimer FGH with Different CT Dressing Schemes (Values in meV)

type of coupling	coupling
direct	0.001
enhanced with NN CT	0.081
enhanced with NNN CT	0.006
enhanced with NN and NNN CT	–0.432

**Figure 12:**
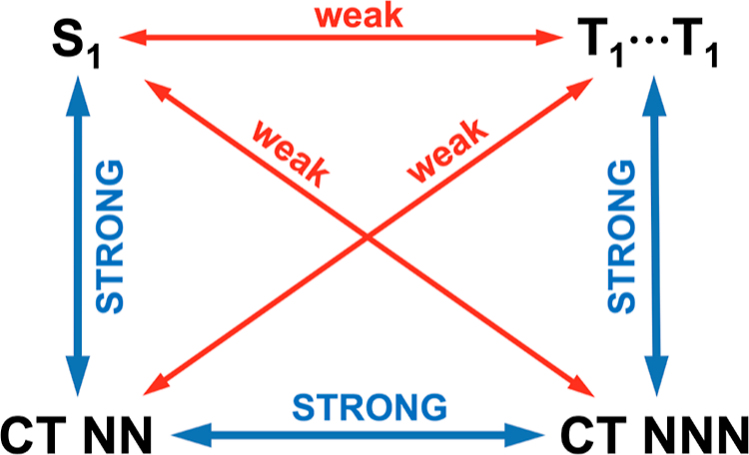
Interaction strengths between the different states and illustration of the *S*_1_ evolution into a spatially separated *T*_1_···*T*_1_ state mediated by strong couplings with CT NN and NNN states. A complete list of couplings via this enhancement mechanism can be found in Table S30 in the Supporting Information.

Additionally, the MFH approach allows the computation of couplings that would otherwise require at least a tetramer model, which are considerably more expensive depending on the molecular units. For example, consider the coupling between two NNN charge transfer MEBFs, F+H– (*D* ^+^ *S*_0_ *D* ^–^ *S*_0_) and G+I– (*S*_0_ *D* ^+^ *S*_0_ *D* ^–^), in Stack 5. Since these MEBFs reside on nonadjacent molecules, the direct coupling is strictly zero; a tetramer model would be needed to capture it directly. However, both F+H– and G+I– can be dressed with NN charge transfer MEBFs with which they interact, resulting in a sizable effective coupling (e.g., −18.7 meV for the F+H– → G+I– case). A similar approach can be applied to couplings between spatially separated triplet states, such as *T*_1_ *S*_0_ *T*_1_ *S*_0_ and *S*_0_ *T*_1_ *S*_0_ *T*_1_. Instead of dressing with CT MEBFs, these triplet configurations are dressed with nearest-neighbor *T*_1_ *T*_1_ MEBFs (namely, *T*_1_ *T*_1_ *S*_0_ *S*_0_, *S*_0_ *T*_1_ *T*_1_ *S*_0_, and *S*_0_ *S*_0_ *T*_1_ *T*_1_). From the studied stacks, the largest such coupling was found to be 0.6 meV in Stack 1, with the mentioned MEBF dressing playing a key role, while for all other stacks the values were close to zero.

## 4 Conclusions

Electronic features of stacks of the chromophore molecule indolonaphthyridine (INDO) have been analyzed. Using the non-orthogonal configuration interaction with fragments method we have calculated the electronic couplings between excited states in dimers and trimers of INDO, using models with different degrees of structural (dis)­order, that is, *ordered* dimers of optimized INDO units or *distorted/disordered* INDO stacks extracted from molecular dynamics.

The ordered models are based on parallel optimized INDO fragments, separated by 3.5 Å. Several conformations were generated with varying rotation angles about the axis perpendicular to the molecular planes and displacements along the short and long molecular axes. In general, these models present large electronic couplings for singlet, triplet, electron and hole diffusion, with values modulated by the structural arrangement adopted. Remarkably, the parallel arrangements with zero displacements along the short and long molecular axes present zero SF couplings. In contrast, we obtained coupling maxima of 450 meV for singlet and electron diffusion in these geometries. Sizeable SF couplings are observed for relatively small displacements along the molecular axes combined with a rotation around the intermolecular axis. Notably, the coupling goes through a maximum for rotation angles of about 20°.

Molecular dynamics suggests rapid formation of INDO stacks presenting varied interplanar distances, slippages, and rotation angles. Each unit is also internally distorted by thermal effects. The electronic couplings calculated for dimers and trimers taken from the stacks show larger SF couplings compared to the ordered model. However, diffusion processes present smaller couplings in general, indicating that the loss of molecular planarity and interfragment coplanarity affects differently the processes studied. Triplet diffusion coupling parameters are small, between ca. 0 and 6 meV. Despite the fact that neighboring units in the stacks present a rather large variation of intermolecular rotations (notably, several being around 20°), we did not recognize the same tendency in the SF coupling as found for the model of parallel undistorted molecules. Likewise, other couplings do not directly correlate with the individual distortions that we determined for the stacks. This indicates that the strength of the coupling is influenced by a complex interplay of different distortions.

The third model is a modification of the previous one. It represents a partial degree of disorder by keeping the interfragment disorder, and removing molecular distortions by using planar fragments. Electronic couplings are affected in diverse ways, that is, molecular planarity greatly enhances singlet diffusion by more than 100 meV in some dimer conformations, whereas SF and triplet diffusion are less affected. Imposing molecular planarity has a large effect on hole and electron diffusion couplings, although the direction of change is hardly predictable.

Finally, we introduce a multifragment full Hamiltonian strategy that allows the extraction of collective data for computationally “unaffordable” systems, such as large (or virtually infinite) molecular stacks. This scalability opens the door to accurately studying large systems with limited additional effort if dimer/trimer NOCI-F data are available. The modularity of the MFH strategy allows reusing fragment wave functions in the various ensembles, avoiding redundant calculations. This multifragment model provides information not available from a smaller model. For instance, we observed that a delocalized wave function for some electronic excitation in an ordered stack becomes strongly localized in specific fragments of the stack when distortions are introduced, demonstrating that structural disorder completely breaks delocalization and trap excitations on monomers. Also, this model gives access to data that otherwise would require at least a highly expensive tetramer model, for example the coupling associated with the *T*_1_ *S*_0_ *T*_1_ *S*_0_ → *S*_0_ *T*_1_ *S*_0_ *T*_1_ process.

## Supplementary Material



## Notes

Energy–time and temperature–time evolution of the INDO/DMF GROMACS simulation. Detailed explanation on particular aspects of the NOCI-F approach (comparison between direct and CT-enhanced couplings; a numerical example illustrating the independence of CT-enhanced couplings on the phase of the fragment wave functions). Tables of electronic couplings for dimers and trimers taken from the INDO stack. Comparison of the electronic couplings obtained with different models of the INDO dimer. Views of the staircase and zigzag regular stack models. Numerical data and graphics illustrating the outcomes of the multifragment full Hamiltonian approach. Atomic *xyz* coordinates of the three stacks generated by molecular dynamics (PDF)
