# Supplementary material for: NOCI‑F Electronic Couplings in Assemblies of Indolonaphthyridine Molecules: From Dimers to the Full Stack
Source: J Chem Theory Comput. 2026 Jan 20;22(3):1296–311. doi: 10.1021/acs.jctc.5c01695 (PMC12895418; doi:10.1021/acs.jctc.5c01695)
Supplement: Supplementary file 1 [file ct5c01695_si_001.pdf]

Supporting Information: NOCI-F electronic couplings in assemblies of indolonaphthyridine molecules: from dimers to the full stack

I.-O. Stan,† T. P. Straatsma,‡,¶ R. Broer,§ C. de Graaf,†,‖ and X. López*,†

†*Universitat Rovira i Virgili, Departament de Química Física i Inorgànica, 43007 Tarragona, Spain*

‡*National Center for Computational Sciences, Oak Ridge National Laboratory, Oak Ridge, TN 37831-6373, U. S. A.*

¶*Department of Chemistry and Biochemistry, University of Alabama, Tuscaloosa, AL 35487-0336, U. S. A.*

§*Zernike Institute for Advanced Materials, University of Groningen, 9747 AG Groningen, The Netherlands*

‖*ICREA, Pg. Lluís Companys 23, 08010 Barcelona, Spain*

E-mail: javier.lopez@urv.cat

S1 Molecular Dynamics

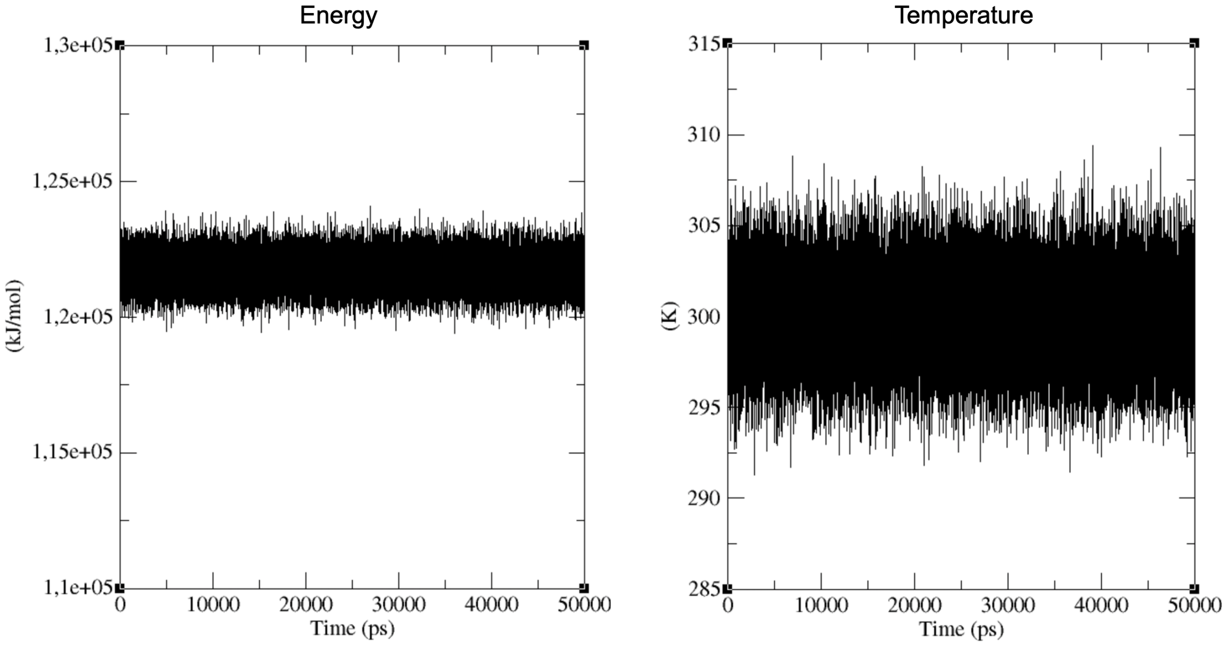

Figure S1: System stability indicators obtained from the molecular dynamics simulation on INDO in DMF. Left: energy (in kJ/mol) vs. time (in ps) plot; right: temperature (in K) vs. time (in ps) plot. The observed fluctuations lie within the typical E and T ranges.

S2 Electronic couplings in the NOCI-F approach

Direct versus charge transfer (CT) enhanced singlet fission coupling. Protocol for extracting the CT-enhanced electronic couplings

In the case of dimers, two sets of new MEBFs are defined by performing partial diagonalizations of the complete NOCI-F matrix. On the one hand, the eigenvectors of the matrix spanned by the S_0S_1, S_1S_0, D^+D^- and D^-D^+ are determined, and on the other hand, the same is done for the T_1T_1, D^+D^-, D^-D^+ sub-block of the Hamiltonian. From the four eigenvectors of the first subdiagonalization, the ones with the largest overlap with the original S_1S_0 and S_0S_1 MEBFs are selected, and from the second diagonalization, the eigenvector with the largest overlap with the T_1T_1 MEBF is taken. Subsequently, a new 3×3 Hamiltonian is constructed with these new 'CT-dressed' MEBFs. The electronic coupling between these new MEBFs, calculated with Eq. 2 of the main text, correspond to the charge-transfer enhanced coupling. The procedure is analogous for trimers.

Phase of the fragment wave functions: A numerical example

Dimer of two randomly distorted benzene molecules (fragment A and B). The second benzene molecule is displaced by (1.25,2.00,3.50) Angstrom with respect to the first one. Case 1 describes the calculation with separately optimized orbitals for the two fragments and is the reference for the other two cases. In case 2, all orbitals of the second molecule are multiplied by -1. In case 3, only the orbitals of the D^- state on fragment B are multiplied by -1. Multiplying the determinants of the CASSCF wave functions by -1 gives identical results as case 1. The phase changes lead to sign changes in the direct couplings between some of the MEBFs, but the final (CT-enhanced) electronic coupling is the same in all cases. Hamiltonian matrix elements and eigenvalues are given in Hartrees, all electronic couplings in meV.

Case 1:

- Hamiltonian

	S_0S_0	S_0S_1	S_1S_0	T_1T_1	D^+D^-	D^-D^+
S_0S_0	-461.1939535	3.5727047	5.5341543	1.1801498	-29.5158268	28.4172177
S_0S_1	3.5727047	-460.9418757	-0.0892329	0.3769184	-13.9802652	0.0882246
S_1S_0	5.5341543	-0.0892329	-460.9378589	0.4259627	-12.4935703	-9.5978644
T_1T_1	1.1801498	0.3769184	0.4259627	-460.9264786	19.4849084	-19.7128004
D^+D^-	-29.5158268	-13.9802652	-12.4935703	19.4849084	-460.9140897	1.2762384
D^-D^+	28.4172177	0.0882246	-9.5978644	-19.7128004	1.2762384	-460.9164700

- Overlap matrix

	S_0S_0	S_0S_1	S_1S_0	T_1T_1	D^+D^-	D^-D^+
S_0S_0	1.0000000	-0.0077524	-0.0119945	-0.0025579	0.0639897	-0.0616069
S_0S_1	-0.0077524	1.0000000	0.0001987	-0.0008172	0.0303191	-0.0001908
S_1S_0	-0.0119945	0.0001987	1.0000000	-0.0009236	0.0270952	0.0208169
T_1T_1	-0.0025579	-0.0008172	-0.0009236	1.0000000	-0.0422557	0.0427493
D^+D^-	0.0639897	0.0303191	0.0270952	-0.0422557	1.0000000	-0.0027662
D^-D^+	-0.0616069	-0.0001908	0.0208169	0.0427493	-0.0027662	1.0000000

- Direct electronic couplings

	S_0S_0	S_0S_1	S_1S_0	T_1T_1	D^+D^-
S_0S_1	-45.5772				
S_1S_0	106.2679	64.3382			
T_1T_1	22.3056	7.1714	6.4073		
D^+D^-	-358.4151	-145.7071	-127.9167	228.4637	
D^-D^+	356.2279	7.0203	-76.4130	-238.5407	34.6065

- Eigenvalues and eigenfunctions of the S_0S_1, S_1S_0, D^+D^-, D^-D^+ sub-block

	Ψ_1	Ψ_2	Ψ_3	Ψ_4
E	-460.94331926	-460.93838993	-460.91677268	-460.91110318
S_0S_1	-0.93071861	-0.30426057	0.08369795	-0.18735932
S_1S_0	0.33964343	-0.91172488	-0.04374973	-0.22947423
D^+D^-	-0.10825096	-0.22211904	-0.34466929	0.90654004
D^-D^+	0.04491756	-0.09123338	0.93443426	0.34195927

- Eigenvalues and eigenfunctions of the T_1T_1, D^+D^-, D^-D^+ sub-block

	Ψ_5	Ψ_6	Ψ_7
E	-460.93391008	-460.91407513	-460.90759304
T_1T_1	-0.81347483	-0.11143669	0.57398317
D^+D^-	0.34542934	0.64014583	0.68751840
D^+D^-	-0.40751436	0.76271525	-0.50401317

- Hamiltonian spanned by the CT-dressed MEBFs involved in the SF process (Ψ_1, Ψ_2, Ψ_5)

	Ψ_1	Ψ_2	Ψ_5
Ψ_1	-460.9433193	0.0000000	32.7424999
Ψ_2	0.0000000	-460.9383899	22.6190767
Ψ_5	32.7424999	22.6190767	-460.9339101

- Overlaps among the CT-dressed MEBFs involved in the SF process (Ψ_1, Ψ_2, Ψ_5)

	Ψ_1	Ψ_2	Ψ_5
Ψ_1	1.0000000	0.0000000	-0.0710314
Ψ_2	0.0000000	1.0000000	-0.0490696
Ψ_3	-0.0710314	-0.0490696	1.0000000

- CT-enhanced electronic couplings

	Ψ_1	Ψ_2
Ψ_2	0.0	
Ψ_3	38.3916	30.6871

Case 2: Multiplying the orbitals of all fragment states on B by -1

- Hamiltonian

	S_0S_0	S_0S_1	S_1S_0	T_1T_1	D^+D^-	D^-D^+
S_0S_0	-461.1939535	3.5727047	5.5341543	1.1801498	29.5158268	-28.4172177
S_0S_1	3.5727047	-460.9418757	-0.0892329	0.3769184	13.9802652	-0.0882246
S_1S_0	5.5341543	-0.0892329	-460.9378589	0.4259627	12.4935703	9.5978644
T_1T_1	1.1801498	0.3769184	0.4259627	-460.9264786	-19.4849084	19.7128004
D^+D^-	29.5158268	13.9802652	12.4935703	-19.4849084	-460.9140897	1.2762384
D^-D^+	-28.4172177	-0.0882246	9.5978644	19.7128004	1.2762384	-460.9164700

- Overlap matrix

	S_0S_0	S_0S_1	S_1S_0	T_1T_1	D^+D^-	D^-D^+
S_0S_0	1.0000000	-0.0077524	-0.0119945	-0.0025579	-0.0639897	0.0616069
S_0S_1	-0.0077524	1.0000000	0.0001987	-0.0008172	-0.0303191	0.0001908
S_1S_0	-0.0119945	0.0001987	1.0000000	-0.0009236	-0.0270952	-0.0208169
T_1T_1	-0.0025579	-0.0008172	-0.0009236	1.0000000	0.0422557	-0.0427493
D^+D^-	-0.0639897	-0.0303191	-0.0270952	0.0422557	1.0000000	-0.0027662
D^-D^+	0.0616069	0.0001908	-0.0208169	-0.0427493	-0.0027662	1.0000000

- Direct electronic couplings

	S_0S_0	S_0S_1	S_1S_0	T_1T_1	D^+D^-
S_0S_1	-45.5772				
S_1S_0	106.2679	64.3382			
T_1T_1	22.3056	7.1714	6.4073		
D^+D^-	358.4151	145.7071	127.9167	-228.4637	
D^-D^+	-356.2279	-7.0203	76.4130	238.5407	34.6065

- Eigenvalues and eigenfunctions of the S_0S_1, S_1S_0, D^+D^-, D^-D^+ sub-block

	Ψ_1	Ψ_2	Ψ_3	Ψ_4
E	-460.94331926	-460.93838993	-460.91677268	-460.91110318
S_0S_1	-0.93071861	-0.30426057	-0.08369795	0.18735932
S_1S_0	0.33964343	-0.91172488	0.04374973	0.22947423
D^+D^-	0.10825096	0.22211904	-0.34466929	0.90654004
D^-D^+	-0.04491756	0.09123338	0.93443426	0.34195927

- Eigenvalues and eigenfunctions of the T_1T_1, D^+D^-, D^-D^+ sub-block

	Ψ_5	Ψ_6	Ψ_7
E	-460.93391008	-460.91407513	-460.90759304
T_1T_1	-0.81347483	0.11143669	-0.57398317
D^+D^-	-0.34542934	0.64014583	0.68751840
D^-D^+	0.40751436	0.76271525	-0.50401317

- Hamiltonian spanned by the CT-dressed MEBFs involved in the SF process (Ψ_1, Ψ_2, Ψ_5)

	Ψ_1	Ψ_2	Ψ_5
Ψ_1	-460.9433193	0.0000000	32.7424999
Ψ_2	0.0000000	-460.9383899	22.6190767
Ψ_5	32.7424999	22.6190767	-460.9339101

- Overlaps among the CT-dressed MEBFs involved in the SF process (Ψ_1, Ψ_2, Ψ_5)

	Ψ_1	Ψ_2	Ψ_5
Ψ_1	1.0000000	0.0000000	-0.0710314
Ψ_2	0.0000000	1.0000000	-0.0490696
Ψ_5	-0.0710314	-0.0490696	1.0000000

- CT-enhanced electronic couplings

	Ψ_1	Ψ_2
Ψ_2	0.0000	
Ψ_3	38.3916	30.6871

Case 3: Multiplying the orbitals of D^- on B by -1

- Hamiltonian

	S_0S_0	S_0S_1	S_1S_0	T_1T_1	D^+D^-	D^-D^+
S_0S_0	-461.1939535	3.5727047	5.5341543	1.1801498	29.5158268	28.4172177
S_0S_1	3.5727047	-460.9418757	-0.0892329	0.3769184	13.9802652	0.0882246
S_1S_0	5.5341543	-0.0892329	-460.9378589	0.4259627	12.4935703	-9.5978644
T_1T_1	1.1801498	0.3769184	0.4259627	-460.9264786	-19.4849084	-19.7128004
D^+D^-	29.5158268	13.9802652	12.4935703	-19.4849084	-460.9140897	-1.2762384
D^-D^+	28.4172177	0.0882246	-9.5978644	-19.7128004	-1.2762384	-460.9164700

- Overlap matrix

	S_0S_0	S_0S_1	S_1S_0	T_1T_1	D^+D^-	D^-D^+
S_0S_0	1.0000000	-0.0077524	-0.0119945	-0.0025579	-0.0639897	-0.0616069
S_0S_1	-0.0077524	1.0000000	0.0001987	-0.0008172	-0.0303191	-0.0001908
S_1S_0	-0.0119945	0.0001987	1.0000000	-0.0009236	-0.0270952	0.0208169
T_1T_1	-0.0025579	-0.0008172	-0.0009236	1.0000000	0.0422557	0.0427493
D^+D^-	-0.0639897	-0.0303191	-0.0270952	0.0422557	1.0000000	0.0027662
D^-D^+	-0.0616069	-0.0001908	0.0208169	0.0427493	0.0027662	1.0000000

- Direct electronic couplings

	S_0S_0	S_0S_1	S_1S_0	T_1T_1	D^+D^-
S_0S_1	-45.5772				
S_1S_0	106.2679	64.3382			
T_1T_1	22.3056	7.1714	6.4073		
D^+D^-	358.4151	145.7071	127.9167	-228.4637	
D^-D^+	356.2279	7.0203	-76.4130	-238.5407	-34.6065

- Eigenvalues and eigenfunctions of the S_0S_1, S_1S_0, D^+D^-, D^-D^+ sub-block

	Ψ_1	Ψ_2	Ψ_3	Ψ_4
E	-460.94331926	-460.93838993	-460.91677268	-460.91110318
S_0S_1	-0.93071861	-0.30426057	0.08369795	0.18735932
S_1S_0	0.33964343	-0.91172488	-0.04374973	0.22947423
D^+D^-	0.10825096	0.22211904	0.34466929	0.90654004
D^-D^+	0.04491756	-0.09123338	0.93443426	-0.34195927

- Eigenvalues and eigenfunctions of the T_1T_1, D^+D^-, D^-D^+ sub-block

	Ψ_5	Ψ_6	Ψ_7
E	-460.93391008	-460.91407513	-460.90759304
T_1T_1	-0.81347483	-0.11143669	-0.57398317
D^+D^-	-0.34542934	-0.64014583	0.68751840
D^-D^+	-0.40751436	0.76271525	0.50401317

- Hamiltonian spanned by the CT-dressed MEBFs involved in the SF process (Ψ_1, Ψ_2, Ψ_5)

	Ψ_1	Ψ_2	Ψ_5
Ψ_1	-460.9433193	0.0000000	32.7424999
Ψ_2	0.0000000	-460.9383899	22.6190767
Ψ_5	32.7424999	22.6190767	-460.9339101

- Overlaps among the CT-dressed MEBFs involved in the SF process (Ψ_1, Ψ_2, Ψ_5)

	Ψ_1	Ψ_2	Ψ_5
Ψ_1	1.0000000	0.0000000	-0.0710314
Ψ_2	0.0000000	1.0000000	-0.0490696
Ψ_5	-0.0710314	-0.0490696	1.0000000

- CT-enhanced electronic couplings

	Ψ_1	Ψ_2
Ψ_2	0.0000	
Ψ_3	38.3916	30.6871

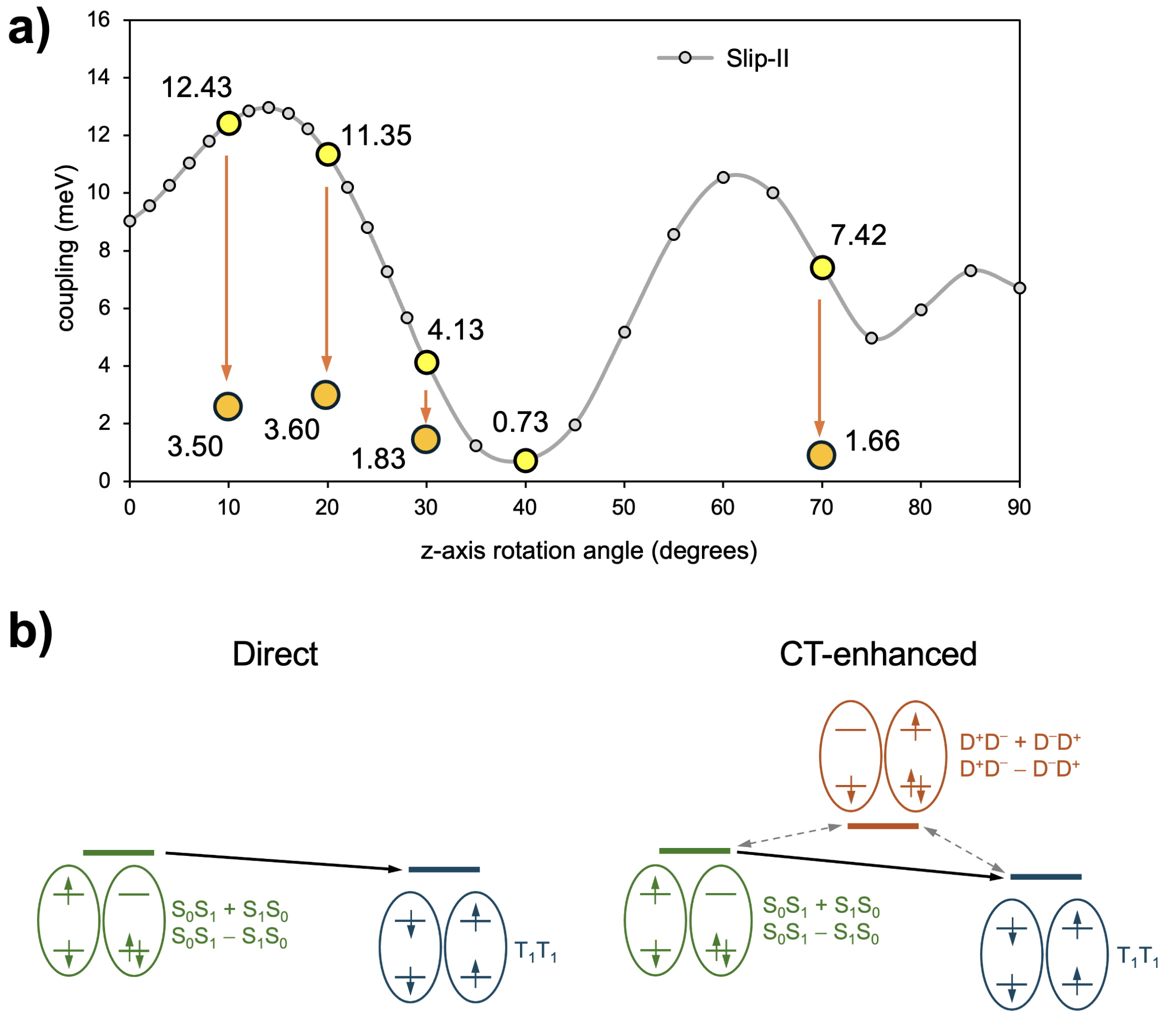

Figure S2: Slip-II regular model. a) Comparison between selected direct (orange dots) and CT-enhanced (gray and yellow dots) singlet fission couplings. b) Schematic representations of the direct and CT-enhanced coupling mechanisms.

S3 Effect of intramolecular distortions on the electronic couplings

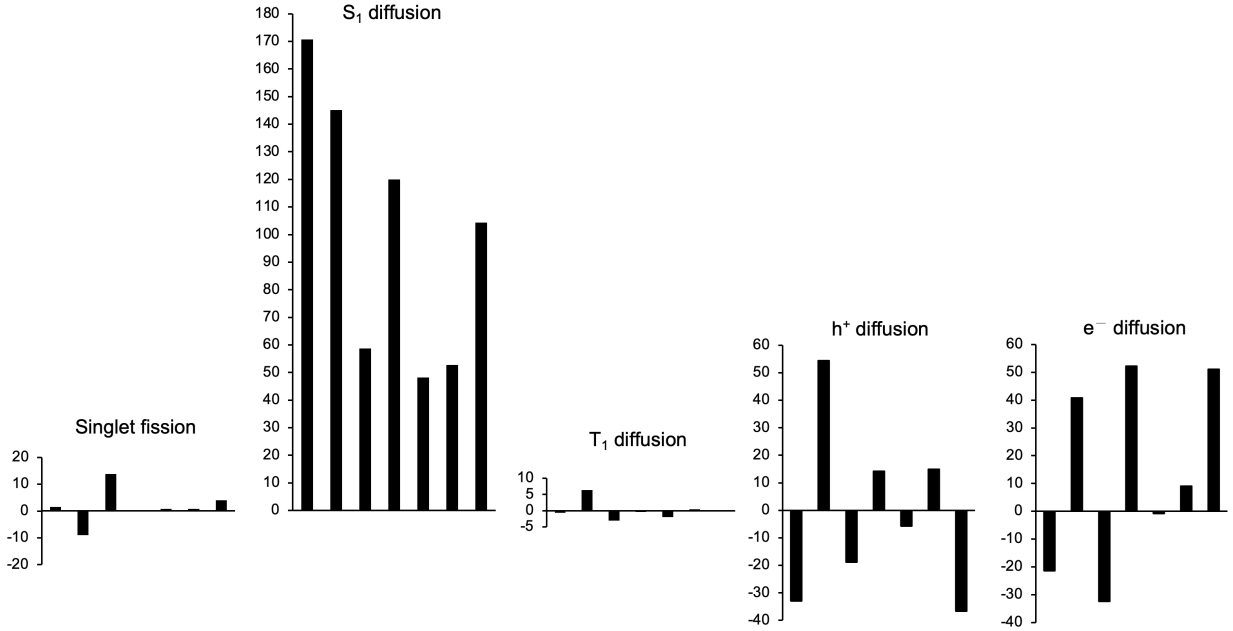

Figure S3: Graphical comparison of the absolute values of the electronic couplings reported in Table 3 for fully distorted vs. disordered planar INDO dimers. A positive bar indicates a larger coupling in the latter case. The vertical scales are in meV.

S4 Regular stacks

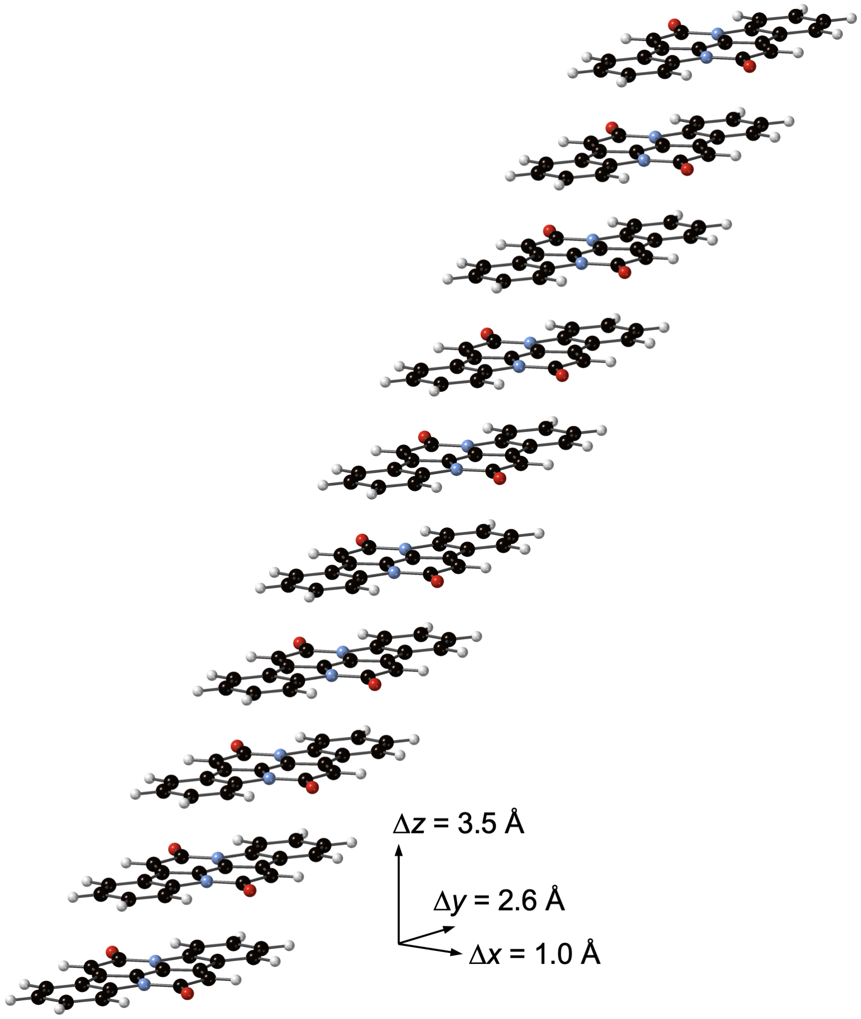

Figure S4: Stack 1. Staircase ordered stack of 10 INDO units arranged following the slip-II motif (see main text for details). The repeating displacements between neighboring units are indicated as $\Delta x, \Delta y, \Delta z$.

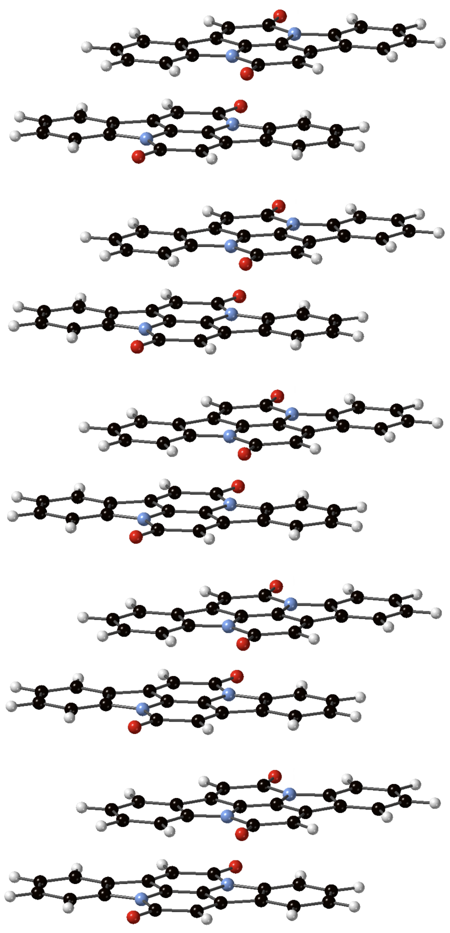

Figure S5: Stack 2. Zig-zag ordered stack of 10 INDO units arranged following the slip-II motif with inter-molecular rotations of 20° (see main text for details). The repeating displacements between neighboring units are $\Delta x = 1.0$ Å, $\Delta y = 2.6$ Å, $\Delta z = 3.5$ Å.

S5 Dimer versus trimer

Electronic couplings obtained from dimer and trimer models. 'Distorted' entries are obtained from Stack 5 (displayed in Figure 4) and 'Planar' entries refer to Stack 6, where planar fragments replace the distorted ones retaining the original relative orientations.

The duplicity of entries, slightly different, in 'trimer' columns arises from the fact that one same coupling can be obtained from two different trimers. For example, the singlet fission B / C → BC processes can be obtained with trimers A$\underline{\text{BC}}$ and $\underline{\text{BC}}$D.

Table S1: Singlet fission coupling (in meV).

	Planar		Distorted	
X/Y → XY	dimer	trimer	dimer	trimer
A / B → AB	3.4	3.2	1.9	1.4
B / C → BC	69.9	72.1 / 70.4	35.0	35.2 / 33.6
C / D → CD	26.9	27.1 / 23.0	13.0	16.8 / 8.8
D / E → DE	2.5	2.4 / 2.6	2.3	2.1 / 2.4
E / F → EF	21.5	21.3 / 22.1	20.7	20.5 / 20.7
F / G → FG	9.6	9.8 / 9.6	8.9	8.1 / 8.5
G / H → GH	9.3	9.4	5.3	4.2

Table S2: Nearest-neighbor coupling (in meV) of the S_1 diffusion.

	Planar		Distorted	
X → Y	dimer	trimer	dimer	trimer
A → B	172.3	154.4	1.6	2.8
B → C	367.3	365.4 / 370.1	95.9	95.9 / 92.9
C → D	63.3	64.6 / 61.9	4.6	4.9 / 6.0
D → E	154.4	155.1 / 155.1	34.4	35.2 / 33.9
E → F	123.1	123.9 / 124.5	74.9	73.9 / 74.7
F → G	186.4	186.2 / 186.5	133.6	137.6 / 135.8
G → H	198.5	198.8	94.2	91.1

Table S3: Next Nearest-neighbor coupling (in meV) of the S_1 diffusion.

	Planar		Distorted	
X → Z	dimer	trimer	dimer	trimer
A → C	–	46.0	–	6.0
B → D	–	41.9	–	4.9
C → E	–	47.0	–	28.4
D → F	–	41.8	–	1.9
E → G	–	42.4	–	46.6
F → H	–	39.1	–	16.2

Table S4: Nearest-neighbor coupling (in meV) of the T_1 diffusion.

	Planar		Distorted	
X → Y	dimer	trimer	dimer	trimer
A → B	0.3	0.3	0.7	0.7
B → C	2.3	2.2 / 2.3	0.4	0.4 / 0.4
C → D	1.0	1.0 / 1.0	5.4	1.2 / 1.2
D → E	2.0	1.9 / 1.9	0.4	1.1 / 1.1
E → F	1.1	1.1 / 1.1	2.2	2.2 / 2.2
F → G	0.2	0.2 / 0.2	1.2	1.2 / 1.2
G → H	0.9	0.9	1.1	1.1

Table S5: Nearest-neighbor coupling (in meV) of the hole diffusion.

	Planar		Distorted	
X+ → Y+	dimer	trimer	dimer	trimer
A+ → B+	84.6	84.5	117.6	117.8
B+ → C+	317.2	314.3 / 317.4	66.2	65.2 / 66.7
C+ → D+	87.2	87.8 / 83.9	106.1	107.3 / 105.0
D+ → E+	30.8	30.3 / 30.4	16.6	16.2 / 16.4
E+ → F+	91.8	92.6 / 91.5	97.5	98.6 / 97.1
F+ → G+	114.5	114.3 / 115.1	99.6	98.9 / 100.3
G+ → H+	6.0	4.9	42.7	41.5

Table S6: Next Nearest-neighbor coupling (in meV) of the hole diffusion.

	Planar		Distorted	
X+ → Z+	dimer	trimer	dimer	trimer
A+ → C+	–	3.8	–	0.2
B+ → D+	–	7.9	–	1.1
C+ → E+	–	17.1	–	14.7
D+ → F+	–	3.9	–	3.9
E+ → G+	–	1.6	–	1.5
F+ → H+	–	1.0	–	0.5

Table S7: Nearest-neighbor coupling (in meV) of the electron diffusion.

	Planar		Distorted	
X- → Y-	dimer	trimer	dimer	trimer
A- → B-	8.7	9.6	30.1	30.7
B- → C-	247.6	248.1 / 249.1	73.5	73.6 / 74.8
C- → D-	37.0	38.7 / 34.7	69.5	70.4 / 68.6
D- → E-	64.0	64.5 / 63.9	11.8	12.5 / 12.5
E- → F-	1.2	2.4 / 2.5	2.0	3.8 / 2.7
F- → G-	40.4	40.5 / 41.5	31.4	30.6 / 32.4
G- → H-	84.5	83.9	33.4	33.4

Table S8: Next Nearest-neighbor coupling (in meV) of the electron diffusion.

	Planar		Distorted	
X- → Z-	dimer	trimer	dimer	trimer
A- → C-	–	2.3	–	0.2
B- → D-	–	10.7	–	1.7
C- → E-	–	1.6	–	9.6
D- → F-	–	13.9	–	12.0
E- → G-	–	9.0	–	8.7
F- → H-	–	0.9	–	5.2

S6 Planarity index

The molecular planarity index (*PI)* has been calculated as follows: i) first, given the *xyz* coordinates of a (non-planar) molecule, the best fitting plane is found by performing a singular value decomposition. Then, the root-mean-square deviation of the distances between all atoms (d_i) and the fitting plane is calculated, providing the value of the planarity index. For a perfectly planar molecule, the index is equal to zero.

$$PI = \sqrt{\frac{1}{N_{atom}} \sum_i d_i^2} \tag{1}$$

S7 Multi-fragment Full Hamiltonian Model

1. Calculation of the main diagonal matrix elements

$$GS_{\text{avg}} = \frac{1}{N}\sum_{i=1}^{N} GS_{\text{di/trimer}} \tag{2}$$

$$H_X = GS_{\text{avg}} + \frac{1}{n}\sum_{i=1}^{n}\left(H_{X,i}^{\text{di/trimer}} - GS_{X,i}^{\text{di/trimer}}\right) \tag{3}$$

Example of the calculation of a matrix element (in hartree units) that appears three times in different trimers (ABC,BCD and CDE):

$$H_C = GS_{\text{avg}} + \frac{1}{3}\left((H_C^{\text{ABC}} - H_{GS}^{\text{ABC}}) + (H_C^{\text{BCD}} - H_{GS}^{\text{BCD}}) + (H_C^{\text{CDE}} - H_{GS}^{\text{CDE}})\right)$$

$$\begin{aligned} H_C = -3066.3476700935 + \frac{1}{3}\Big(&(-3066.200025929 + 3066.3308203221) \\ &+ (-3066.2003725025 + 3066.3301536176) \\ &+ (-3066.2176343707 + 3066.3487906022)\Big) = -3066.2170928469 \end{aligned}$$

2. Calculation of the off-diagonal matrix elements

$$H_{\text{ij}} = \frac{1}{N}\sum_{k=1}^{N} H_{ij,k}^{\text{di/trimer}} \tag{4}$$

Example of the calculation of a matrix element (in hartree units) that appears two times in different trimers (ABC and BCD):

$$\begin{aligned} \langle BC|\hat{H}|C\rangle &= \frac{1}{2}\left(\langle BC|\hat{H}|C\rangle_{ABC} + \langle BC|\hat{H}|C\rangle_{BCD}\right) \\ &= \frac{1}{2}(0.4663861388 + 0.4646641356) = 0.465525137 \end{aligned}$$

Table S9: Overview of all singlet MEBFs in a 9-molecule stack.

Full label	Short label	Character
$S_0S_0S_0S_0S_0S_0S_0S_0S_0$	GS	Ground state
$S_1S_0S_0S_0S_0S_0S_0S_0S_0$	A	Local excited singlet
$S_0S_1S_0S_0S_0S_0S_0S_0S_0$	B	Local excited singlet
$S_0S_0S_1S_0S_0S_0S_0S_0S_0$	C	Local excited singlet
$S_0S_0S_0S_1S_0S_0S_0S_0S_0$	D	Local excited singlet
$S_0S_0S_0S_0S_1S_0S_0S_0S_0$	E	Local excited singlet
$S_0S_0S_0S_0S_0S_1S_0S_0S_0$	F	Local excited singlet
$S_0S_0S_0S_0S_0S_0S_1S_0S_0$	G	Local excited singlet
$S_0S_0S_0S_0S_0S_0S_0S_1S_0$	H	Local excited singlet
$S_0S_0S_0S_0S_0S_0S_0S_0S_1$	I	Local excited singlet
$T_1T_1S_0S_0S_0S_0S_0S_0S_0$	AB	Coupled double triplet
$S_0T_1T_1S_0S_0S_0S_0S_0S_0$	BC	Coupled double triplet
$S_0S_0T_1T_1S_0S_0S_0S_0S_0$	CD	Coupled double triplet
$S_0S_0S_0T_1T_1S_0S_0S_0S_0$	DE	Coupled double triplet
$S_0S_0S_0S_0T_1T_1S_0S_0S_0$	EF	Coupled double triplet
$S_0S_0S_0S_0S_0T_1T_1S_0S_0$	FG	Coupled double triplet
$S_0S_0S_0S_0S_0S_0T_1T_1S_0$	GH	Coupled double triplet
$S_0S_0S_0S_0S_0S_0S_0T_1T_1$	HI	Coupled double triplet
$T_1S_0T_1S_0S_0S_0S_0S_0S_0$	AC	Separated double triplet
$S_0T_1S_0T_1S_0S_0S_0S_0S_0$	BD	Separated double triplet
$S_0S_0T_1S_0T_1S_0S_0S_0S_0$	CE	Separated double triplet
$S_0S_0S_0T_1S_0T_1S_0S_0S_0$	DF	Separated double triplet
$S_0S_0S_0S_0T_1S_0T_1S_0S_0$	EG	Separated double triplet

Continued on next page

Table S9 – continued from previous page

Full label	Short label	Character
$S_0S_0S_0S_0S_0T_1S_0T_1S_0$	FH	Separated double triplet
$S_0S_0S_0S_0S_0S_0T_1S_0T_1$	HI	Separated double triplet
$D^+D^-S_0S_0S_0S_0S_0S_0S_0$	A+B-	Nearest-neighbor CT
$D^-D^+S_0S_0S_0S_0S_0S_0S_0$	A-B+	Nearest-neighbor CT
$S_0D^+D^-S_0S_0S_0S_0S_0S_0$	B+C-	Nearest-neighbor CT
$S_0D^-D^+S_0S_0S_0S_0S_0S_0$	B-C+	Nearest-neighbor CT
$S_0S_0D^+D^-S_0S_0S_0S_0S_0$	C+D-	Nearest-neighbor CT
$S_0S_0D^-D^+S_0S_0S_0S_0S_0$	C-D+	Nearest-neighbor CT
$S_0S_0S_0D^+D^-S_0S_0S_0S_0$	D+E-	Nearest-neighbor CT
$S_0S_0S_0D^-D^+S_0S_0S_0S_0$	D-E+	Nearest-neighbor CT
$S_0S_0S_0S_0D^+D^-S_0S_0S_0$	E+F-	Nearest-neighbor CT
$S_0S_0S_0S_0D^-D^+S_0S_0S_0$	E-F+	Nearest-neighbor CT
$S_0S_0S_0S_0S_0D^+D^-S_0S_0$	F+G-	Nearest-neighbor CT
$S_0S_0S_0S_0S_0D^-D^+S_0S_0$	F-G+	Nearest-neighbor CT
$S_0S_0S_0S_0S_0S_0D^+D^-S_0$	G+H-	Nearest-neighbor CT
$S_0S_0S_0S_0S_0S_0D^-D^+S_0$	G-H+	Nearest-neighbor CT
$S_0S_0S_0S_0S_0S_0S_0D^+D^-$	H+I-	Nearest-neighbor CT
$S_0S_0S_0S_0S_0S_0S_0D^-D^+$	H-I+	Nearest-neighbor CT
$D^+S_0D^-S_0S_0S_0S_0S_0S_0$	A+C-	Next Nearest-neighbor CT
$D^-S_0D^+S_0S_0S_0S_0S_0S_0$	A-C+	Next Nearest-neighbor CT
$S_0D^+S_0D^-S_0S_0S_0S_0S_0$	B+D-	Next Nearest-neighbor CT
$S_0D^-S_0D^+S_0S_0S_0S_0S_0$	B-D+	Next Nearest-neighbor CT
$S_0S_0D^+S_0D^-S_0S_0S_0S_0$	C+E-	Next Nearest-neighbor CT

Continued on next page

Table S9 – continued from previous page

Full label	Short label	Character
$S_0S_0D^-S_0D^+S_0S_0S_0S_0$	C-E+	Next Nearest-neighbor CT
$S_0S_0S_0D^+S_0D^-S_0S_0S_0$	D+F-	Next Nearest-neighbor CT
$S_0S_0S_0D^-S_0D^+S_0S_0S_0$	D-F+	Next Nearest-neighbor CT
$S_0S_0S_0S_0D^+S_0D^-S_0S_0$	E+G-	Next Nearest-neighbor CT
$S_0S_0S_0S_0D^-S_0D^+S_0S_0$	E-G+	Next Nearest-neighbor CT
$S_0S_0S_0S_0S_0D^+S_0D^-S_0$	F+H-	Next Nearest-neighbor CT
$S_0S_0S_0S_0S_0D^-S_0D^+S_0$	F-H+	Next Nearest-neighbor CT
$S_0S_0S_0S_0S_0S_0D^+S_0D^-$	G+I-	Next Nearest-neighbor CT
$S_0S_0S_0S_0S_0S_0D^-S_0D^+$	G-I+	Next Nearest-neighbor CT

Table S10: MEBFs composition (in %) and relative energies (in eV) of the NOCI wave functions for Stack 1.

NOCI WF	GS	S_1	T_1T_1	$T_1...T_1$	CT NN	CT NNN	ΔE
1	98.1	0.1	0.0	0.0	1.8	0.0	0.00
2	0.1	57.3	40.3	0.0	2.4	0.0	1.68
3	0.0	42.3	55.3	0.1	2.3	0.0	1.69
4	0.0	17.4	80.3	0.5	1.8	0.0	1.70
5	0.0	4.3	93.1	1.4	1.2	0.0	1.70
6	0.0	0.9	95.4	2.9	0.9	0.0	1.70
7	0.0	0.1	94.5	4.7	0.8	0.0	1.70
8	0.0	0.3	95.1	3.8	0.8	0.0	1.70
9	0.0	0.4	95.7	3.0	0.8	0.0	1.70
10	0.0	0.2	94.1	5.1	0.6	0.0	1.70
11	0.0	40.7	59.0	0.2	0.1	0.0	1.72
12	0.0	55.0	43.7	0.8	0.5	0.0	1.72
13	0.0	66.0	13.4	19.4	1.3	0.0	1.74
14	0.0	0.1	0.3	99.6	0.0	0.0	1.74
15	0.0	0.4	1.3	98.3	0.0	0.0	1.74
16	0.0	4.1	2.7	93.1	0.1	0.0	1.74
17	0.0	8.5	5.2	86.2	0.1	0.0	1.74
18	0.0	0.1	3.2	96.7	0.0	0.0	1.74
19	0.0	0.9	4.4	94.7	0.0	0.0	1.74
20	0.0	0.0	4.7	95.2	0.0	0.0	1.74
21	0.0	0.1	5.6	94.3	0.0	0.0	1.74
22	0.0	91.7	5.4	0.1	2.8	0.0	1.77

Continued on next page

Table S10 – continued from previous page

NOCI WF	GS	S_1	T_1T_1	$T_1...T_1$	CT NN	CT NNN	ΔE
23	0.0	94.3	1.6	0.0	4.1	0.0	1.81
24	0.0	93.8	0.5	0.0	5.6	0.1	1.87
25	0.0	92.0	0.2	0.0	7.7	0.1	1.93
26	0.0	89.8	0.1	0.0	10.1	0.1	1.99
27	0.0	86.9	0.0	0.0	12.8	0.2	2.03
28	0.0	84.6	0.0	0.0	15.1	0.2	2.06
29	0.1	0.1	1.1	0.0	94.3	4.5	2.82
30	0.1	0.2	1.0	0.0	94.5	4.2	2.83
31	0.1	0.5	0.9	0.0	95.0	3.5	2.84
32	0.1	0.7	0.9	0.0	95.1	3.3	2.85
33	0.1	1.4	0.6	0.0	95.6	2.4	2.87
34	0.1	0.6	0.4	0.0	96.9	2.1	2.87
35	0.1	0.5	0.1	0.0	98.0	1.4	2.87
36	0.1	2.0	0.2	0.0	96.2	1.6	2.88
37	0.1	2.0	0.2	0.0	96.1	1.6	2.88
38	0.1	4.3	0.6	0.0	92.9	2.2	2.91
39	0.1	5.0	0.5	0.0	91.8	2.6	2.92
40	0.1	3.4	0.8	0.0	94.0	1.8	2.92
41	0.1	8.0	0.2	0.0	88.6	3.1	2.93
42	0.1	5.4	0.5	0.0	86.6	5.1	2.93
43	0.1	7.9	0.4	0.0	86.7	3.9	2.95
44	0.1	12.3	0.1	0.0	84.1	3.5	2.96
45	0.1	12.3	0.2	0.0	84.4	3.0	2.98

Continued on next page

Table S10 – continued from previous page

NOCI WF	GS	S_1	T_1T_1	$T_1...T_1$	CT NN	CT NNN	ΔE
46	0.1	16.4	0.1	0.0	79.0	4.5	2.98
47	0.0	0.0	0.0	0.0	1.7	98.3	3.83
48	0.0	0.0	0.0	0.0	1.8	98.2	3.83
49	0.0	0.0	0.0	0.0	2.3	97.7	3.84
50	0.0	0.0	0.0	0.0	2.3	97.7	3.84
51	0.0	0.0	0.0	0.0	3.0	97.0	3.85
52	0.0	0.0	0.0	0.0	3.1	96.9	3.85
53	0.0	0.0	0.0	0.0	4.0	96.0	3.86
54	0.0	0.0	0.0	0.0	4.0	95.9	3.86
55	0.0	0.0	0.0	0.0	4.8	95.1	3.87
56	0.0	0.1	0.0	0.0	5.1	94.9	3.87
57	0.0	0.0	0.0	0.0	5.6	94.3	3.87
58	0.0	0.2	0.0	0.0	6.1	93.8	3.88
59	0.0	0.0	0.0	0.0	6.1	93.8	3.88
60	0.0	0.3	0.0	0.0	7.0	92.7	3.89
61	0.0	0.4	0.0	0.0	7.6	92.0	3.89
62	0.0	0.0	0.0	0.0	7.7	92.1	3.89

Table S11: Electronic coupling γ (in meV) calculated from the trimers ABC, BCD, CDE, and from the MFH approach in Stack 2.

Coupling	ABC	BCD	CDE	Avg	MFH
$\gamma_{C,GS}$	-21.47	59.30	-21.43	5.46	5.46
$\gamma_{C,BC}$	0.89	5.09	–	2.98	2.98
$\gamma_{B+C-,BC}$	165.16	-47.93	–	58.61	58.09

Table S12: Direct Couplings (in meV) calculated from individual trimers and from the MFH approach in Stack 3.

Coupling	ABC	BCD	CDE	Avg	MFH
$\gamma_{C,GS}$	24.76	8.14	25.96	19.62	27.46
$\gamma_{C,BC}$	7.26	7.22	–	7.24	6.91
$\gamma_{B+C-,BC}$	188.15	189.55	–	188.85	176.80

Table S13: Hamiltonian Matrix of the CDE trimer from the trimer calculation of Stack 2 (in hartree).

	GS	C	D	E	CD	DE	CE	C+D-	C-D+	D+E-	D-E+	C+E-	C-E+
GS	-3066.10	-6.06	12.89	-6.06	0.68	0.68	-0.01	106.72	-26.88	-26.88	106.72	-2.96	-2.96
C	-6.06	-3065.95	-0.27	0.02	0.37	0.00	0.00	-48.40	-32.18	0.04	0.14	11.82	-4.75
D	12.89	-0.27	-3065.95	-0.27	1.33	1.33	0.00	-35.66	-47.36	-47.36	-35.66	-0.37	-0.37
E	-6.06	0.02	-0.27	-3065.95	0.00	0.37	0.00	0.14	0.04	-32.18	-48.40	-4.75	11.82
CD	0.68	0.37	1.33	0.00	-3065.94	0.02	-0.55	-20.83	81.19	-0.26	-0.04	-0.41	0.83
DE	0.68	0.00	1.33	0.37	0.02	-3065.94	-0.55	-0.04	-0.26	81.19	-20.83	0.83	-0.41
CE	-0.01	0.00	0.00	0.00	-0.55	-0.55	-3065.94	-0.06	-0.03	-0.03	-0.06	-2.15	-2.15
C+D-	106.72	-48.40	-35.66	0.14	-20.83	-0.04	-0.06	-3065.94	0.32	0.35	-8.64	-51.91	0.03
C-D+	-26.88	-32.18	-47.36	0.04	81.19	-0.26	-0.03	0.32	-3065.94	10.58	0.35	0.09	-25.63
D+E-	-26.88	0.04	-47.36	-32.18	-0.26	81.19	-0.03	0.35	10.58	-3065.94	0.32	-25.63	0.09
D-E+	106.72	0.14	-35.66	-48.40	-0.04	-20.83	-0.06	-8.64	0.35	0.32	-3065.94	0.03	-51.91
C+E-	-2.96	11.82	-0.37	-4.75	-0.41	0.83	-2.15	-51.91	0.09	-25.63	0.03	-3065.92	0.02
C-E+	-2.96	-4.75	-0.37	11.82	0.83	-0.41	-2.15	0.03	-25.63	0.09	-51.91	0.02	-3065.92

Table S14: Hamiltonian Matrix of the CDE trimer from Stack 2 after applying the MFH approach (in hartree).

	GS	C	D	E	CD	DE	CE	C+D-	C-D+	D+E-	D-E+	C+E-	C-E+
GS	-3066.10	0.26	0.26	0.26	0.68	0.68	-0.01	39.92	39.92	39.92	39.92	-2.96	-2.96
C	0.26	-3065.95	-0.27	0.02	0.85	0.00	0.00	-47.88	-33.92	0.04	0.14	11.82	-4.75
D	0.26	-0.27	-3065.95	-0.27	0.85	0.85	0.00	-33.92	-47.88	-47.88	-33.92	-0.37	-0.37
E	0.26	0.02	-0.27	-3065.95	0.00	0.85	0.00	0.14	0.04	-33.92	-47.88	-4.75	11.82
CD	0.68	0.85	0.85	0.00	-3065.94	0.02	-0.55	30.18	30.18	-0.26	-0.04	-0.41	0.83
DE	0.68	0.00	0.85	0.85	0.02	-3065.94	-0.55	-0.04	-0.26	30.18	30.18	0.83	-0.41
CE	-0.01	0.00	0.00	0.00	-0.55	-0.55	-3065.94	-0.06	-0.03	-0.03	-0.06	-2.15	-2.15
C+D-	39.92	-47.88	-33.92	0.14	30.18	-0.04	-0.06	-3065.94	0.32	0.35	-8.64	-51.91	0.03
C-D+	39.92	-33.92	-47.88	0.04	30.18	-0.26	-0.03	0.32	-3065.94	10.58	0.35	0.09	-25.63
D+E-	39.92	0.04	-47.88	-33.92	-0.26	30.18	-0.03	0.35	10.58	-3065.94	0.32	-25.63	0.09
D-E+	39.92	0.14	-33.92	-47.88	-0.04	30.18	-0.06	-8.64	0.35	0.32	-3065.94	0.03	-51.91
C+E-	-2.96	11.82	-0.37	-4.75	-0.41	0.83	-2.15	-51.91	0.09	-25.63	0.03	-3065.92	0.02
C-E+	-2.96	-4.75	-0.37	11.82	0.83	-0.41	-2.15	0.03	-25.63	0.09	-51.91	0.02	-3065.92

Table S15: NOCI-F wave function energies (in eV) in the original CDE trimer calculation and in Stack 2, and their difference.

NOCI WF	ΔE trimer	ΔE stack	Difference stack-trimer
Ψ_1	0.000	0.000	0.000
Ψ_2	4.075	4.065	0.010
Ψ_3	4.100	4.092	0.008
Ψ_4	4.185	4.181	0.004
Ψ_5	4.279	4.335	-0.056
Ψ_6	4.323	4.364	-0.041
Ψ_7	4.401	4.395	0.006
Ψ_8	4.549	4.517	0.032
Ψ_9	4.552	4.540	0.012
Ψ_{10}	4.670	4.583	0.087
Ψ_{11}	4.709	4.626	0.083
Ψ_{12}	5.126	5.117	0.009
Ψ_{13}	5.127	5.118	0.009

Table S16: Hamiltonian Matrix of the CDE trimer from the trimer calculation of Stack 5 (in hartree).

	GS	C	D	E	CD	DE	CE	C+D-	C-D+	D+E-	D-E+	C+E-	C-E+
GS	-3066.35	-86.28	-112.20	0.18	-1.22	-0.15	0.01	36.27	-134.69	22.71	11.57	-7.08	3.92
C	-86.28	-3066.22	-2.95	0.49	0.63	-0.01	-0.01	-11.11	8.28	0.57	0.33	2.10	1.38
D	-112.20	-2.95	-3066.22	-0.03	0.42	-0.20	0.00	-4.07	5.27	-17.81	-2.79	-0.04	0.14
E	0.18	0.49	-0.03	-3066.21	0.00	0.40	0.00	-0.02	0.03	1.05	5.09	-3.64	-4.51
CD	-1.22	0.63	0.42	0.00	-3066.24	-0.01	0.16	107.65	-41.97	0.09	-0.18	-0.07	0.02
DE	-0.15	-0.01	-0.20	0.40	-0.01	-3066.23	-0.31	0.02	-0.02	6.26	-14.32	0.12	-0.16
CE	0.01	-0.01	0.00	0.00	0.16	-0.31	-3066.23	0.00	0.03	-0.02	-0.07	2.52	-5.10
C+D-	36.27	-11.11	-4.07	-0.02	107.65	0.02	0.00	-3066.22	2.72	-0.70	3.76	3.30	0.00
C-D+	-134.69	8.28	5.27	0.03	-41.97	-0.02	0.03	2.72	-3066.21	5.22	0.21	-0.42	6.30
D+E-	22.71	0.57	-17.81	1.05	0.09	6.26	-0.02	-0.70	5.22	-3066.20	0.04	48.42	-0.03
D-E+	11.57	0.33	-2.79	5.09	-0.18	-14.32	-0.07	3.76	0.21	0.04	-3066.22	0.02	-32.30
C+E-	-7.08	2.10	-0.04	-3.64	-0.07	0.12	2.52	3.30	-0.42	48.42	0.02	-3066.18	0.00
C-E+	3.92	1.38	0.14	-4.51	0.02	-0.16	-5.10	0.00	6.30	-0.03	-32.30	0.00	-3066.18

Table S17: Hamiltonian Matrix of the CDE trimer from Stack 5 after applying the MFH approach (in hartree).

	GS	C	D	E	CD	DE	CE	C+D-	C-D+	D+E-	D-E+	C+E-	C-E+
GS	-3066.35	-81.98	-110.75	7.08	-1.22	-0.15	0.01	36.33	-134.61	22.57	11.59	-7.08	3.92
C	-81.98	-3066.21	-2.62	0.49	0.63	-0.01	-0.01	-11.09	8.35	0.57	0.33	2.10	1.38
D	-110.75	-2.62	-3066.22	0.31	0.42	-0.19	0.00	-4.11	5.25	-17.67	-2.86	-0.04	0.14
E	7.08	0.49	0.31	-3066.23	0.00	0.39	0.00	-0.02	0.03	1.02	5.02	-3.64	-4.51
CD	-1.22	0.63	0.42	0.00	-3066.24	-0.01	0.16	107.57	-42.02	0.09	-0.18	-0.07	0.02
DE	-0.15	-0.01	-0.19	0.39	-0.01	-3066.25	-0.31	0.02	-0.02	6.22	-14.20	0.12	-0.16
CE	0.01	-0.01	0.00	0.00	0.16	-0.31	-3066.24	0.00	0.03	-0.02	-0.07	2.52	-5.10
C+D-	36.33	-11.09	-4.11	-0.02	107.57	0.02	0.00	-3066.22	2.72	-0.70	3.76	3.30	0.00
C-D+	-134.61	8.35	5.25	0.03	-42.02	-0.02	0.03	2.72	-3066.21	5.22	0.21	-0.42	6.30
D+E-	22.57	0.57	-17.67	1.02	0.09	6.22	-0.02	-0.70	5.22	-3066.21	0.04	48.42	-0.03
D-E+	11.59	0.33	-2.86	5.02	-0.18	-14.20	-0.07	3.76	0.21	0.04	-3066.23	0.02	-32.30
C+E-	-7.08	2.10	-0.04	-3.64	-0.07	0.12	2.52	3.30	-0.42	48.42	0.02	-3066.19	0.00
C-E+	3.92	1.38	0.14	-4.51	0.02	-0.16	-5.10	0.00	6.30	-0.03	-32.30	0.00	-3066.19

Table S18: NOCI-F wave function energies (in eV) in the original CDE trimer and in Stack 5, and their difference.

NOCI WF	ΔE trimer	ΔE stack	Difference stack-trimer
Ψ_1	0.000	0.000	0.000
Ψ_2	2.823	2.818	0.005
Ψ_3	3.170	3.169	0.001
Ψ_4	3.186	3.207	-0.021
Ψ_5	3.539	3.542	-0.002
Ψ_6	3.587	3.575	0.012
Ψ_7	3.604	3.605	-0.001
Ψ_8	3.628	3.659	-0.030
Ψ_9	3.852	3.817	0.035
Ψ_{10}	3.870	3.884	-0.014
Ψ_{11}	4.091	4.071	0.020
Ψ_{12}	4.492	4.491	0.001
Ψ_{13}	4.652	4.650	0.002

Table S19: Singlet fission couplings (in meV).

X/Y → XY	Stack 1	Stack 2	Stack 3	Stack 4	Stack 5	Stack 6
A / B → AB	9.1	10.2	10.9	3.4	1.6	2.0
B / C → BC	8.9	11.0	21.6	9.8	31.3	49.8
C / D → CD	8.9	11.0	15.7	7.9	11.8	20.0
D / E → DE	8.9	11.0	13.0	16.7	2.3	2.8
E / F → EF	8.9	11.0	26.3	4.0	22.6	26.4
F / G → FG	8.9	11.0	8.5	17.6	9.2	12.5
G / H → GH	8.9	11.0	16.4	32.1	5.3	11.7
H / I → HI	8.9	11.0	25.2	3.5	–	–
I / J → IJ	9.1	10.2	–	37.3	–	–

Table S20: Nearest-neighbor electronic couplings (in meV) for S_1 diffusion.

X → Y	Stack 1	Stack 2	Stack 3	Stack 4	Stack 5	Stack 6
A → B	98.2	89.8	24.6	25.4	2.5	157.9
B → C	96.4	89.6	26.5	70.4	92.7	333.3
C → D	96.2	89.6	57.6	22.2	5.2	63.7
D → E	96.2	89.6	24.1	10.3	33.3	154.5
E → F	96.2	89.6	34.3	18.1	74.2	122.9
F → G	96.2	89.6	16.2	27.1	136.7	186.8
G → H	96.2	89.6	9.0	120.5	90.9	197.3
H → I	96.4	89.6	2.1	9.5	–	–
I → J	98.2	89.8	–	14.5	–	–

Table S21: Next nearest-neighbor electronic couplings (in meV) for S_1 diffusion.

X → Z	Stack 1	Stack 2	Stack 3	Stack 4	Stack 5	Stack 6
A → C	19.3	57.6	15.1	1.0	5.9	46.6
B → D	19.0	57.2	0.1	11.6	4.9	43.5
C → E	18.9	57.2	5.6	5.5	26.6	42.9
D → F	18.9	57.2	5.6	1.3	1.8	41.1
E → G	18.9	57.2	1.4	15.6	46.1	41.3
F → H	18.9	57.2	8.9	1.4	15.9	38.8
G → I	19.0	57.2	3.5	14.7	–	–
H → J	19.3	57.6	–	7.6	–	–

Table S22: Nearest-neighbor electronic couplings (in meV) for T_1 diffusion.

X → Y	Stack 1	Stack 2	Stack 3	Stack 4	Stack 5	Stack 6
A → B	14.0	6.3	1.9	0.1	2.2	0.4
B → C	14.0	6.2	5.8	1.5	4.7	70.8
C → D	14.0	6.2	4.0	4.0	9.5	3.3
D → E	14.0	6.2	3.2	12.6	0.4	1.1
E → F	14.0	6.2	5.0	10.2	0.4	0.6
F → G	14.0	6.2	7.7	3.4	2.8	5.5
G → H	14.0	6.2	2.5	5.8	2.3	1.4
H → I	14.0	6.2	22.0	6.8	–	–
I → J	14.0	6.3	–	33.1	–	–

Table S23: Next nearest-neighbor electronic couplings (in meV) for T_1 diffusion.

X → Z	Stack 1	Stack 2	Stack 3	Stack 4	Stack 5	Stack 6
A → C	0.0	0.1	0.0	0.0	0.0	0.0
B → D	0.0	0.1	0.0	0.0	0.0	0.2
C → E	0.0	0.1	0.0	0.1	0.1	0.1
D → F	0.0	0.1	0.0	0.1	0.0	0.1
E → G	0.0	0.1	0.1	0.0	0.1	0.1
F → H	0.0	0.1	0.1	0.3	0.0	0.0
G → I	0.0	0.1	0.0	0.1	–	–
H → J	0.0	0.1	–	0.0	–	–

The values represented as 0.0 in the table are < 0.05 meV.

Table S24: Electronic couplings (in meV) for T_1T_1 diffusion.

XY → YZ	Stack 1	Stack 2	Stack 3	Stack 4	Stack 5	Stack 6
AB → BC	0.2	0.4	7.4	0.0	0.1	0.1
BC → CD	0.2	0.2	0.3	0.1	0.1	0.9
CD → DE	0.2	0.2	0.4	0.5	0.1	0.1
DE → EF	0.2	0.2	0.6	1.4	0.1	0.1
EF → FG	0.2	0.2	0.4	0.6	0.1	0.3
FG → GH	0.2	0.2	0.1	0.2	0.0	0.2
GH → HI	0.2	0.2	2.6	0.2	–	–
HI → IJ	0.2	0.4	–	0.2	–	–

The values represented as 0.0 in the table are < 0.05 meV.

Table S25: Electronic couplings (in meV) for T_1T_1 separation.

XY / YZ → XZ	Stack 1	Stack 2	Stack 3	Stack 4	Stack 5	Stack 6
AB → AC	4.4	1.8	0.8	1.1	0.4	14.7
BC → AC	4.4	1.8	0.2	0.2	0.3	0.1
BC → BD	4.4	1.8	1.9	0.7	1.0	0.1
CD → BD	4.4	1.8	0.2	1.1	0.2	13.2
CD → CE	4.4	1.8	0.3	1.1	1.0	1.4
DE → CE	4.4	1.8	1.8	0.7	1.1	0.0
DE → DF	4.4	1.8	0.2	0.5	1.9	1.1
EF → DF	4.4	1.8	0.4	0.4	1.0	1.5
EF → EG	4.4	1.8	1.7	0.5	0.3	1.3
FG → EG	4.4	1.8	0.2	0.3	2.0	1.2
FG → FH	4.4	1.8	0.7	4.2	1.3	1.1
GH → FH	4.4	1.8	1.9	0.4	0.4	1.2
GH → GI	4.4	1.8	6.0	1.5	–	–
HI → GI	4.4	1.8	1.0	4.3	–	–
HI → HJ	4.4	1.8	–	10.1	–	–
IJ → HJ	4.4	1.8	–	1.0	–	–

Table S26: Nearest-neighbor electronic couplings (in meV) for hole diffusion.

X+ → Y+	Stack 1	Stack 2	Stack 3	Stack 4	Stack 5	Stack 6
A+ → B+	70.2	52.4	6.5	22.0	117.9	85.7
B+ → C+	70.2	52.5	77.8	32.5	65.4	303.1
C+ → D+	70.2	52.5	22.3	67.9	104.8	84.8
D+ → E+	70.2	52.5	55.6	73.5	16.4	31.9
E+ → F+	70.2	52.5	106.6	0.6	100.6	92.6
F+ → G+	70.2	52.5	162.3	114.2	99.0	115.0
G+ → H+	70.2	52.5	7.8	126.0	40.0	4.9
H+ → I+	70.2	52.5	211.6	98.0	–	–
I+ → J+	70.2	52.4	–	108.3	–	–

Table S27: Nearest-neighbor electronic couplings (in meV) for hole diffusion.

X+ → Z+	Stack 1	Stack 2	Stack 3	Stack 4	Stack 5	Stack 6
A+ → C+	1.1	8.0	1.6	2.1	0.2	3.7
B+ → D+	1.1	8.1	0.3	2.6	1.0	7.6
C+ → E+	1.1	8.0	3.1	0.5	14.7	18.1
D+ → F+	1.1	8.0	1.7	8.0	4.0	4.0
E+ → G+	1.1	8.0	3.7	0.0	1.5	1.6
F+ → H+	1.1	8.0	8.0	12.8	0.5	1.0
G+ → I+	1.1	8.1	1.8	4.2	–	–
H+ → J+	1.1	8.0	–	0.2	–	–

Table S28: Nearest-neighbor electronic couplings (in meV) for electron diffusion.

X- → Y-	Stack 1	Stack 2	Stack 3	Stack 4	Stack 5	Stack 6
A- → B-	181.3	115.9	165.3	20.0	30.7	9.9
B- → C-	181.1	115.8	64.6	73.1	73.6	237.1
C- → D-	181.2	115.9	39.4	64.0	68.8	36.3
D- → E-	181.2	115.9	49.1	174.4	12.4	66.8
E- → F-	181.2	115.9	86.0	125.4	3.3	2.4
F- → G-	181.2	115.9	0.2	43.6	31.3	41.2
G- → H-	181.2	115.9	158.9	102.2	32.9	83.7
H- → I-	181.1	115.8	92.2	97.2	–	–
I- → J-	181.3	115.9	–	167.9	–	–

Table S29: Next nearest-neighbor electronic couplings (in meV) for electron diffusion.

X- $\rightarrow$ Z-	Stack 1	Stack 2	Stack 3	Stack 4	Stack 5	Stack 6
A- $\rightarrow$ C-	5.1	24.1	5.9	5.4	0.1	2.5
B- $\rightarrow$ D-	5.1	24.1	1.7	2.4	1.4	10.4
C- $\rightarrow$ E-	5.1	24.1	8.1	7.3	9.5	2.1
D- $\rightarrow$ F-	5.1	24.1	1.7	20.7	12.2	14.3
E- $\rightarrow$ G-	5.1	24.1	2.6	2.8	8.9	9.1
F- $\rightarrow$ H-	5.1	24.1	9.2	20.7	5.1	1.0
G- $\rightarrow$ I-	5.1	24.1	2.4	10.9	–	–
H- $\rightarrow$ J-	5.1	24.1	–	2.0	–	–

Table S30: Electronic couplings (in meV) for direct singlet fission.

X/Y/Z → XZ	Stack 1	Stack 2	Stack 3	Stack 4	Stack 5	Stack 6
A → AC	0.0	0.0	0.2	0.0	0.0	0.0
B → AC	0.0	0.2	0.1	0.0	0.0	0.1
C → AC	0.0	0.0	0.1	0.0	0.0	0.1
B → BD	0.0	0.0	0.3	0.0	0.1	0.2
C → BD	0.0	0.2	0.1	0.1	0.1	0.4
D → BD	0.0	0.0	0.1	0.1	0.0	0.0
C → CE	0.0	0.0	0.1	0.1	0.0	0.1
D → CE	0.0	0.2	0.1	0.2	0.0	0.0
E → CE	0.0	0.0	0.0	0.0	0.0	0.2
D → DF	0.0	0.0	0.0	0.1	0.0	0.1
E → DF	0.0	0.2	0.2	0.1	0.0	0.0
F → DF	0.0	0.0	0.0	0.0	0.0	0.1
E → EG	0.0	0.0	0.0	0.0	0.0	0.0
F → EG	0.0	0.2	0.2	0.0	0.1	0.1
G → EG	0.0	0.0	0.2	0.0	0.1	0.1
F → FH	0.0	0.0	0.1	0.2	0.0	0.0
G → FH	0.0	0.2	0.5	0.6	0.0	0.0
H → FH	0.0	0.0	0.3	0.2	0.1	0.0
G → GI	0.0	0.0	0.2	0.1	–	–
H → GI	0.0	0.2	0.1	0.3	–	–
I → GI	0.0	0.0	0.0	0.3	–	–
H → HJ	0.0	0.0	–	0.3	–	–
I → HJ	0.0	0.2	–	0.3	–	–
J → HJ	0.0	0.0	–	0.0	–	–

The values represented as 0.0 in the table are < 0.05 meV.

S8 Coordinates of the MD stacks

Table S31: Coordinates (in Å) of the 9-fragment INDO Stack 3. Snapshot taken at t = 49.5 ns of the MD simulation in DMF with a starting stacked conformation.

C	-5.57000	-1.05000	3.90000
C	-6.13000	-1.90000	3.03000
C	-6.78000	-1.08000	2.11000
C	-7.59000	-1.44000	1.08000
C	-8.17000	-0.49000	0.18000
C	-8.02000	0.92000	0.54000
C	-7.19000	1.30000	1.67000
C	-6.56000	0.26000	2.36000
C	-5.15000	1.31000	4.24000
C	-6.00000	-3.27000	3.41000
C	-4.91000	-1.34000	5.02000
C	-4.35000	-0.44000	5.78000
C	-3.64000	-1.20000	6.69000
C	-2.77000	-0.80000	7.74000
C	-2.20000	-1.78000	8.55000
C	-2.41000	-3.17000	8.30000
C	-3.30000	-3.55000	7.32000
C	-3.93000	-2.57000	6.52000
C	-5.23000	-3.64000	4.63000
C	-4.46000	0.92000	5.39000
N	-5.74000	0.26000	3.48000
N	-4.76000	-2.60000	5.44000

Continued on next page

Table S31 – continued from previous page

O	-5.13000	2.52000	3.89000
O	-5.07000	-4.86000	4.87000
H	-7.71000	-2.51000	0.91000
H	-8.69000	-0.84000	-0.70000
H	-8.41000	1.71000	-0.10000
H	-7.11000	2.30000	2.08000
H	-6.42000	-4.06000	2.80000
H	-2.52000	0.23000	7.95000
H	-1.48000	-1.48000	9.30000
H	-1.81000	-3.89000	8.84000
H	-3.63000	-4.57000	7.17000
H	-3.82000	1.65000	5.87000
C	-2.37000	-2.30000	2.70000
C	-2.00000	-3.55000	3.08000
C	-1.23000	-3.37000	4.19000
C	-0.66000	-4.33000	4.99000
C	0.11000	-3.93000	6.09000
C	0.31000	-2.59000	6.30000
C	-0.29000	-1.58000	5.51000
C	-1.04000	-1.99000	4.43000
C	-2.12000	0.06000	3.16000
C	-2.44000	-4.66000	2.32000
C	-3.14000	-1.97000	1.73000
C	-3.46000	-0.73000	1.25000
C	-4.28000	-1.00000	0.18000

Continued on next page

Table S31 – continued from previous page

C	-4.95000	-0.08000	-0.66000
C	-5.67000	-0.61000	-1.73000
C	-5.69000	-2.01000	-2.03000
C	-4.98000	-2.88000	-1.14000
C	-4.29000	-2.38000	-0.07000
C	-3.24000	-4.34000	1.18000
C	-2.98000	0.36000	2.06000
N	-1.90000	-1.30000	3.50000
N	-3.61000	-3.01000	0.98000
O	-1.54000	0.91000	3.85000
O	-3.53000	-5.21000	0.42000
H	-0.77000	-5.40000	4.83000
H	0.40000	-4.73000	6.74000
H	0.90000	-2.25000	7.14000
H	-0.17000	-0.54000	5.77000
H	-2.10000	-5.62000	2.67000
H	-4.83000	0.99000	-0.50000
H	-6.11000	0.05000	-2.46000
H	-6.12000	-2.39000	-2.94000
H	-4.88000	-3.95000	-1.26000
H	-3.20000	1.39000	1.82000
C	-10.36000	1.31000	9.17000
C	-10.24000	2.63000	9.09000
C	-9.31000	2.90000	10.06000
C	-8.78000	4.14000	10.37000

Continued on next page

Table S31 – continued from previous page

C	-7.76000	4.26000	11.42000
C	-7.38000	3.11000	12.07000
C	-7.93000	1.87000	11.79000
C	-8.88000	1.77000	10.76000
C	-9.51000	-0.67000	10.17000
C	-10.89000	3.28000	8.02000
C	-11.05000	0.53000	8.33000
C	-11.01000	-0.85000	8.28000
C	-11.72000	-1.17000	7.20000
C	-11.95000	-2.47000	6.71000
C	-12.65999	-2.64000	5.51000
C	-13.13999	-1.52000	4.86000
C	-12.85999	-0.20000	5.33000
C	-12.13000	-0.03000	6.52000
C	-11.62000	2.47000	7.04000
C	-10.14000	-1.49000	9.23000
N	-9.61000	0.75000	10.19000
N	-11.69000	1.12000	7.28000
O	-8.88000	-1.26000	11.03000
O	-12.15000	2.89000	6.04000
H	-9.05000	5.05000	9.86000
H	-7.56000	5.24000	11.82000
H	-6.82000	3.32000	12.96999
H	-7.80000	0.96000	12.35000
H	-10.84000	4.35000	7.87000

Continued on next page

Table S31 – continued from previous page

H	-11.53000	-3.32000	7.23000
H	-12.86999	-3.63000	5.12000
H	-13.82999	-1.62000	4.03000
H	-13.29999	0.58000	4.74000
H	-10.04000	-2.56000	9.31000
C	5.12000	1.33000	-4.39000
C	4.66000	2.46000	-4.81000
C	5.23000	3.33000	-3.89000
C	5.05000	4.74000	-3.80000
C	5.76000	5.53000	-2.86000
C	6.63000	4.86000	-1.94000
C	6.75000	3.43000	-1.94000
C	6.09000	2.74000	-2.97000
C	6.66000	0.26000	-2.77000
C	3.86000	2.44000	-5.93000
C	4.82000	0.08000	-4.84000
C	5.20000	-1.09000	-4.37000
C	4.68000	-1.98000	-5.26000
C	4.75000	-3.39000	-5.11000
C	4.15000	-4.01000	-6.21000
C	3.44000	-3.35000	-7.28000
C	3.27000	-1.96000	-7.20000
C	3.94000	-1.29000	-6.21000
C	3.57000	1.21000	-6.55000
C	6.07000	-1.05000	-3.22000

Continued on next page

Table S31 – continued from previous page

N	5.98000	1.40000	-3.29000
N	4.05000	0.05000	-5.94000
O	7.55000	0.39000	-1.96000
O	2.90000	1.27000	-7.59000
H	4.31000	5.18000	-4.46000
H	5.67000	6.60000	-2.84000
H	7.12000	5.38000	-1.13000
H	7.37000	2.95000	-1.21000
H	3.44000	3.39000	-6.24000
H	5.30000	-3.91000	-4.34000
H	4.11000	-5.08000	-6.31000
H	3.10000	-4.01000	-8.06000
H	2.66000	-1.46000	-7.94000
H	6.46000	-2.03000	-3.00000
C	3.88000	0.09000	-0.69000
C	3.99000	1.41000	-0.57000
C	4.88000	1.61000	0.49000
C	5.20000	2.83000	1.10000
C	5.88000	2.78000	2.31000
C	6.29000	1.55000	2.90000
C	6.07000	0.37000	2.18000
C	5.25000	0.41000	1.02000
C	4.59000	-2.06000	0.20000
C	3.26000	2.17000	-1.48000
C	3.18000	-0.60000	-1.54000

Continued on next page

Table S31 – continued from previous page

C	3.09000	-1.96000	-1.65000
C	2.22000	-2.08000	-2.63000
C	1.79000	-3.28000	-3.23000
C	1.01000	-3.21000	-4.42000
C	0.57000	-1.98000	-4.92000
C	0.89000	-0.80000	-4.16000
C	1.80000	-0.88000	-3.13000
C	2.36000	1.50000	-2.38000
C	3.74000	-2.74000	-0.75000
N	4.61000	-0.60000	0.28000
N	2.39000	0.11000	-2.39000
O	5.20000	-2.70000	1.01000
O	1.62000	2.11000	-3.13000
H	4.84000	3.81000	0.81000
H	5.96000	3.62000	2.98000
H	6.79000	1.58000	3.85000
H	6.55000	-0.55000	2.48000
H	3.27000	3.25000	-1.47000
H	1.98000	-4.28000	-2.87000
H	0.90000	-4.15000	-4.95000
H	-0.07000	-1.98000	-5.79000
H	0.54000	0.19000	-4.40000
H	3.60000	-3.80000	-0.63000
C	7.71000	1.30000	-6.56000
C	8.57000	1.46000	-5.56000

Continued on next page

Table S31 – continued from previous page

C	8.87000	0.17000	-5.19000
C	9.62000	-0.30000	-4.06000
C	9.58000	-1.73000	-3.82000
C	8.90000	-2.58000	-4.75000
C	8.27000	-2.05000	-5.86000
C	8.21000	-0.70000	-6.11000
C	6.80000	-0.25000	-8.13000
C	8.85000	2.74000	-5.15000
C	7.28000	2.24000	-7.38000
C	6.59000	2.16000	-8.51000
C	6.38000	3.40000	-8.94000
C	5.70000	3.77000	-10.10000
C	5.57000	5.18000	-10.30000
C	6.05000	6.07000	-9.37000
C	6.80000	5.65000	-8.21000
C	6.95000	4.29000	-8.04000
C	8.37000	3.91000	-5.91000
C	6.32000	0.79000	-8.96000
N	7.45000	0.02000	-6.94000
N	7.57000	3.55000	-7.05000
O	6.62000	-1.42000	-8.43000
O	8.57000	5.08000	-5.65000
H	10.05000	0.40000	-3.36000
H	10.12000	-2.22000	-3.03000
H	8.88000	-3.65000	-4.60000

Continued on next page

Table S31 – continued from previous page

H	7.81000	-2.68000	-6.62000
H	9.59000	2.88000	-4.37000
H	5.29000	3.07000	-10.82000
H	5.13000	5.54000	-11.22000
H	5.92000	7.13000	-9.47000
H	7.08000	6.36000	-7.45000
H	5.89000	0.54000	-9.92000
C	-1.07000	-0.57000	-1.31000
C	-1.27000	-1.82000	-1.58000
C	-2.09000	-1.87000	-2.70000
C	-2.62000	-3.01000	-3.45000
C	-3.48000	-2.81000	-4.55000
C	-3.65000	-1.46000	-4.96000
C	-3.10000	-0.39000	-4.24000
C	-2.31000	-0.58000	-3.10000
C	-1.43000	1.65000	-2.20000
C	-0.58000	-2.75000	-0.73000
C	-0.25000	-0.03000	-0.41000
C	0.01000	1.23000	-0.19000
C	0.83000	1.25000	0.89000
C	1.46000	2.31000	1.54000
C	2.20000	1.99000	2.67000
C	2.61000	0.69000	2.88000
C	2.00000	-0.38000	2.23000
C	1.05000	-0.07000	1.27000

Continued on next page

Table S31 – continued from previous page

C	0.32000	-2.28000	0.31000
C	-0.59000	2.13000	-1.12000
N	-1.67000	0.28000	-2.21000
N	0.38000	-0.86000	0.44000
O	-1.96000	2.33000	-2.99000
O	0.96000	-3.06000	0.99000
H	-2.51000	-4.03000	-3.11000
H	-3.97000	-3.66000	-5.00000
H	-4.30000	-1.32000	-5.81000
H	-3.25000	0.64000	-4.52000
H	-0.69000	-3.82000	-0.73000
H	1.33000	3.32000	1.18000
H	2.60000	2.81000	3.25000
H	3.30000	0.54000	3.69000
H	2.26000	-1.40000	2.47000
H	-0.35000	3.18000	-1.09000
C	-7.42000	-1.12000	7.46000
C	-7.40000	-2.45000	7.60000
C	-6.69000	-2.62000	8.78000
C	-6.33000	-3.82000	9.39000
C	-5.50000	-3.82000	10.48000
C	-5.12000	-2.56000	11.01000
C	-5.55000	-1.34000	10.47000
C	-6.35000	-1.41000	9.31000
C	-6.82000	0.97000	8.43000

Continued on next page

Table S31 – continued from previous page

C	-8.06000	-3.13000	6.51000
C	-7.96000	-0.42000	6.49000
C	-8.11000	0.92000	6.43000
C	-8.80000	1.19000	5.29000
C	-9.31000	2.44000	4.85000
C	-10.17000	2.45000	3.70000
C	-10.51000	1.22000	3.13000
C	-10.04000	-0.01000	3.65000
C	-9.11000	0.01000	4.69000
C	-8.63000	-2.40000	5.41000
C	-7.50000	1.69000	7.44000
N	-6.84000	-0.45000	8.45000
N	-8.61000	-1.01000	5.48000
O	-6.13000	1.68000	9.18000
O	-9.04000	-3.00000	4.48000
H	-6.64000	-4.72000	8.89000
H	-5.13000	-4.74000	10.92000
H	-4.48000	-2.53000	11.88000
H	-5.24000	-0.39000	10.87000
H	-8.03000	-4.21000	6.49000
H	-9.04000	3.36000	5.36000
H	-10.47000	3.35000	3.18000
H	-11.24000	1.19000	2.33000
H	-10.28000	-0.96000	3.20000
H	-7.50000	2.77000	7.43000

Continued on next page

Table S31 – continued from previous page

C	10.78000	2.00000	-8.55000
C	11.48000	2.75000	-7.68000
C	11.14000	4.05000	-7.98000
C	11.32000	5.24000	-7.26000
C	10.72000	6.45000	-7.71000
C	9.88000	6.39000	-8.87000
C	9.71000	5.23000	-9.58000
C	10.30000	4.05000	-9.10000
C	9.33000	2.08000	-10.49000
C	12.12000	1.98000	-6.67000
C	10.71000	0.65000	-8.66000
C	10.00000	-0.10000	-9.50000
C	10.28000	-1.39000	-9.18000
C	9.75000	-2.45000	-9.85000
C	10.14000	-3.72000	-9.31000
C	11.13000	-3.79000	-8.23000
C	11.64000	-2.62000	-7.63000
C	11.18000	-1.43000	-8.14000
C	12.20000	0.51000	-6.78000
C	9.23000	0.64000	-10.48000
N	10.12000	2.70000	-9.56000
N	11.44000	-0.12000	-7.80000
O	8.59000	2.73000	-11.21000
O	12.74999	-0.10000	-5.84000
H	11.86000	5.29000	-6.33000

Continued on next page

Table S31 – continued from previous page

H	10.82000	7.37000	-7.17000
H	9.47000	7.32000	-9.23000
H	9.22000	5.20000	-10.55000
H	12.65999	2.46000	-5.86000
H	9.07000	-2.35000	-10.69000
H	9.80000	-4.63000	-9.77000
H	11.46000	-4.72000	-7.82000
H	12.42000	-2.68000	-6.88000
H	8.49000	0.16000	-11.08000

Table S32: Coordinates (in Å) of the 10-fragment INDO Stack 4. Snapshot taken at t = 45.0 ns of the MD simulation in DMF.

C	28.97000	20.83000	14.82000
C	28.03000	20.01000	15.27000
C	28.66000	18.86000	15.59000
C	28.16000	17.59000	16.04000
C	29.11000	16.55000	16.21000
C	30.48000	16.74000	16.04999
C	30.96000	17.97000	15.55000
C	30.05000	19.03000	15.32000
C	31.33000	21.04999	14.42000
C	26.69000	20.50000	15.27000
C	28.84000	22.03000	14.37000
C	29.80000	22.93000	14.06000
C	29.11000	24.04000	13.72000
C	29.58000	25.28000	13.34000
C	28.63000	26.32000	13.14000
C	27.26000	26.13000	13.33000
C	26.77000	24.90000	13.86000
C	27.74000	23.86000	13.97000
C	26.45000	21.86000	14.87000
C	31.14000	22.43000	14.10000
N	30.24000	20.34000	14.90000
N	27.57000	22.58000	14.37000
O	32.44000	20.51000	14.38000
O	25.36000	22.39000	14.97000

Continued on next page

Table S32 – continued from previous page

H	27.10000	17.38000	16.06000
H	28.76000	15.56000	16.47000
H	31.10000	15.85000	16.04999
H	32.02000	18.05000	15.36000
H	25.93000	19.91000	15.75000
H	30.59000	25.45000	12.99000
H	28.92000	27.23000	12.63000
H	26.58000	26.96000	13.21000
H	25.73000	24.74000	14.07000
H	31.87000	23.15000	13.78000
C	28.34000	24.47000	17.24000
C	27.85000	23.44000	17.85000
C	28.81000	22.55000	17.95000
C	28.70000	21.23000	18.44000
C	29.89000	20.52000	18.52000
C	31.11000	21.10000	18.12000
C	31.19000	22.39000	17.65000
C	30.02000	23.12000	17.49000
C	30.44000	25.45999	16.50000
C	26.48000	23.51000	18.12000
C	27.82000	25.63000	17.04000
C	28.39000	26.73000	16.46000
C	27.34000	27.56000	16.22000
C	27.34000	28.83000	15.59000
C	26.09000	29.49000	15.45000

Continued on next page

Table S32 – continued from previous page

C	24.86000	28.85000	15.78000
C	24.93000	27.52000	16.29000
C	26.20000	26.95000	16.63000
C	25.73000	24.65000	17.77000
C	29.73000	26.65000	16.15000
N	29.71000	24.44000	17.13000
N	26.45000	25.71000	17.23000
O	31.60000	25.33000	16.27000
O	24.53000	24.87000	17.97000
H	27.77000	20.74000	18.68000
H	29.91000	19.52000	18.92000
H	32.04000	20.57000	18.26000
H	32.17000	22.79000	17.43000
H	26.00000	22.74000	18.71000
H	28.26000	29.34000	15.38000
H	26.01000	30.43000	14.94000
H	23.91000	29.27000	15.50000
H	23.99000	27.10000	16.60000
H	30.25000	27.50000	15.74000
C	27.71000	27.84000	19.71000
C	26.95000	26.88000	20.18000
C	27.76000	25.90000	20.52000
C	27.44000	24.66000	20.96000
C	28.48000	23.73000	21.24000
C	29.79000	24.06000	21.11999

Continued on next page

Table S32 – continued from previous page

C	30.11000	25.30000	20.53000
C	29.05000	26.26000	20.32000
C	30.00000	28.46000	19.47000
C	25.54000	27.14000	20.27000
C	27.39000	28.99000	19.31000
C	28.19000	30.02000	18.94000
C	27.42000	31.07000	18.63000
C	27.81000	32.36000	18.14000
C	26.79000	33.29000	17.98000
C	25.42000	32.91000	18.31000
C	25.05000	31.58000	18.64000
C	26.13000	30.64000	18.82000
C	25.05000	28.41000	19.82000
C	29.59000	29.75000	18.95000
N	29.04000	27.57000	19.90000
N	26.06000	29.28000	19.26000
O	31.20000	28.19000	19.55000
O	23.89000	28.69000	19.78000
H	26.43000	24.39000	21.22000
H	28.14000	22.80000	21.67000
H	30.58000	23.40000	21.43000
H	31.10000	25.60000	20.20000
H	24.96000	26.31000	20.63000
H	28.83000	32.59000	17.88999
H	27.05000	34.28000	17.63000

Continued on next page

Table S32 – continued from previous page

H	24.62000	33.60000	18.11000
H	24.03000	31.28000	18.86000
H	30.28000	30.51000	18.61000
C	25.81000	27.63000	23.58000
C	25.40000	26.41000	23.84000
C	26.49000	25.65000	24.07000
C	26.60000	24.30000	24.39000
C	27.93000	23.83000	24.74000
C	29.05000	24.68000	24.58000
C	28.91000	26.02000	24.13999
C	27.59000	26.42000	23.84000
C	27.76000	28.94000	23.19000
C	24.01000	26.24000	23.76000
C	25.12000	28.69000	23.18000
C	25.60000	29.87000	22.78000
C	24.53000	30.61000	22.32000
C	24.38000	31.93000	21.80999
C	23.21000	32.27000	21.28000
C	22.11000	31.44000	21.36000
C	22.19000	30.18000	21.99000
C	23.46000	29.79000	22.36999
C	23.15000	27.29999	23.32000
C	26.97000	30.06000	22.84000
N	27.16000	27.72000	23.52000
N	23.75000	28.56000	23.07000

Continued on next page

Table S32 – continued from previous page

O	28.94000	29.16000	23.21000
O	21.96000	27.10000	23.10000
H	25.71000	23.70000	24.41000
H	27.96000	22.82000	25.10000
H	30.01000	24.23000	24.79999
H	29.77000	26.66000	24.00000
H	23.63000	25.25000	23.96000
H	25.23000	32.58000	21.68000
H	23.07000	33.32000	21.07000
H	21.18000	31.73000	20.88999
H	21.31000	29.57000	22.13000
H	27.39000	30.94000	22.38000
C	27.21000	30.24000	26.15000
C	27.10000	31.43000	25.60000
C	25.77000	31.72000	25.60000
C	25.16000	32.95000	25.15000
C	23.77000	33.04000	25.23000
C	23.03000	31.96000	25.76000
C	23.67000	30.75000	26.13000
C	25.07000	30.66000	25.98000
C	25.86000	28.30000	26.79000
C	28.30000	32.09000	25.30000
C	28.28000	29.58000	26.43000
C	28.31000	28.28000	26.78000
C	29.67000	27.97000	26.68000

Continued on next page

Table S32 – continued from previous page

C	30.35000	26.75000	26.85000
C	31.73000	26.71000	26.69000
C	32.47000	27.96000	26.56000
C	31.79000	29.13000	26.27000
C	30.37000	29.12000	26.31000
C	29.51000	31.45000	25.58000
C	27.10000	27.63000	27.03000
N	25.97000	29.66000	26.40000
N	29.45000	30.10000	26.03000
O	24.77000	27.76000	26.83000
O	30.60000	32.01000	25.38999
H	25.77000	33.81000	24.93000
H	23.30000	33.99000	25.04000
H	21.95000	31.96000	25.83000
H	23.17000	29.91000	26.59000
H	28.41000	33.07000	24.84000
H	29.68000	25.92000	27.00000
H	32.29000	25.80000	26.66000
H	33.54000	27.98000	26.40000
H	32.29000	30.07000	26.10000
H	27.14000	26.59000	27.28000
C	28.92000	31.59000	29.07000
C	28.73000	30.35000	29.59000
C	29.98000	29.82000	29.75000
C	30.38999	28.66000	30.38000

Continued on next page

Table S32 – continued from previous page

C	31.78000	28.41000	30.57000
C	32.69000	29.40000	30.20000
C	32.27000	30.52000	29.37000
C	30.89000	30.76000	29.27000
C	30.59000	33.23000	28.60000
C	27.38000	30.06000	29.93000
C	27.98000	32.48000	28.72000
C	28.19000	33.74000	28.31000
C	26.92000	34.28000	28.29000
C	26.54000	35.59000	28.00000
C	25.19000	35.94000	28.12000
C	24.18000	35.00000	28.52000
C	24.63000	33.68000	28.81000
C	25.98000	33.37000	28.72000
C	26.34000	31.00000	29.61000
C	29.53000	34.16000	28.25000
N	30.24000	31.96000	28.91000
N	26.69000	32.22000	29.10000
O	31.73000	33.60000	28.59000
O	25.10000	30.79000	29.68000
H	29.62000	28.03000	30.81000
H	32.08000	27.63000	31.25000
H	33.73000	29.37000	30.51000
H	32.97000	31.27000	29.03000
H	27.16000	29.12000	30.40000

Continued on next page

Table S32 – continued from previous page

H	27.22000	36.36000	27.68000
H	24.79999	36.92000	27.88999
H	23.15000	35.31000	28.56000
H	23.90000	32.93000	29.06000
H	29.72000	35.22000	28.18000
C	27.17000	35.13000	31.65000
C	27.38000	36.38000	31.27000
C	26.14000	36.91000	31.08000
C	25.79000	38.22000	30.69000
C	24.39000	38.55000	30.56000
C	23.40000	37.59000	30.98000
C	23.84000	36.30000	31.39000
C	25.18000	36.02000	31.46000
C	25.45000	33.55000	32.26000
C	28.79000	36.61000	31.19000
C	28.06000	34.23000	32.09999
C	27.81000	33.03000	32.67000
C	29.03000	32.41000	32.70000
C	29.33000	31.09000	33.13999
C	30.68000	30.68000	33.20000
C	31.70000	31.58000	32.70000
C	31.33000	32.85000	32.19000
C	30.01000	33.26000	32.24000
C	29.86000	35.70000	31.38000
C	26.45000	32.66000	32.81000

Continued on next page

Table S32 – continued from previous page

N	25.86000	34.83000	31.78000
N	29.40000	34.47000	31.92000
O	24.24000	33.29000	32.21000
O	31.03000	36.00000	31.22000
H	26.61000	38.89000	30.48000
H	24.11000	39.51000	30.15000
H	22.35000	37.81000	30.96000
H	23.12000	35.51000	31.54000
H	29.19000	37.53000	30.80000
H	28.51000	30.40000	33.30000
H	31.01000	29.68000	33.43000
H	32.72000	31.22000	32.60000
H	32.12000	33.41000	31.72000
H	26.22000	31.63999	33.07000
C	30.58000	34.66000	35.14000
C	31.49000	33.78000	35.48000
C	32.64000	34.28000	35.06000
C	33.96000	33.69000	35.00000
C	34.98000	34.41000	34.45999
C	34.79000	35.72000	33.96000
C	33.48000	36.19000	33.87000
C	32.45999	35.49000	34.50000
C	30.41000	36.85000	34.25000
C	30.94000	32.60000	36.03000
C	29.25000	34.61000	35.29000

Continued on next page

Table S32 – continued from previous page

C	28.42000	35.54999	34.97000
C	27.24000	35.04000	35.42000
C	25.96000	35.59000	35.32000
C	24.87000	34.81000	35.61000
C	25.05000	33.49000	36.06000
C	26.36000	32.93000	36.28000
C	27.43000	33.77000	35.93000
C	29.53000	32.43000	36.25000
C	28.97000	36.75000	34.38999
N	31.14000	35.74000	34.60000
N	28.74000	33.51000	35.85000
O	30.92000	37.93000	33.97000
O	29.08000	31.42000	36.77000
H	34.09000	32.69000	35.39000
H	36.00000	34.06000	34.47000
H	35.62000	36.30000	33.59000
H	33.39000	37.01000	33.16000
H	31.58000	31.78000	36.31000
H	25.93000	36.59000	34.91000
H	23.88000	35.23000	35.56000
H	24.13999	32.94000	36.26000
H	26.44000	31.92000	36.65000
H	28.36000	37.58000	34.06000
C	29.84000	35.73000	38.59000
C	28.51000	35.72000	38.75000

Continued on next page

Table S32 – continued from previous page

C	28.25000	34.50000	39.38999
C	27.01000	33.88000	39.70000
C	27.00000	32.63000	40.33000
C	28.25000	32.08000	40.75000
C	29.48000	32.62000	40.31000
C	29.45000	33.91000	39.63000
C	31.91000	34.55000	39.19000
C	27.81000	36.83000	38.26000
C	30.54000	36.60000	37.99000
C	31.86000	36.62000	37.91000
C	32.19000	37.86000	37.43000
C	33.43000	38.49000	37.15000
C	33.46000	39.79000	36.60000
C	32.25000	40.52000	36.29000
C	31.02000	39.87000	36.55000
C	31.04000	38.52000	37.02000
C	28.59000	37.88000	37.58000
C	32.59000	35.58000	38.48000
N	30.51000	34.72000	39.20000
N	29.96000	37.67000	37.46000
O	32.47000	33.51000	39.63000
O	28.03000	38.84000	37.14000
H	26.06000	34.32000	39.44000
H	26.04999	32.16000	40.51000
H	28.15000	31.15000	41.29000

Continued on next page

Table S32 – continued from previous page

H	30.43000	32.18000	40.57000
H	26.74000	36.99000	38.34000
H	34.37000	37.96000	37.20000
H	34.32000	40.38999	36.36000
H	32.33000	41.57000	36.09000
H	30.07000	40.36000	36.38000
H	33.66000	35.58000	38.38000
C	26.22000	36.65000	42.13000
C	26.12000	37.80000	41.53000
C	24.79999	38.18000	41.66000
C	24.18000	39.38999	41.28000
C	22.82000	39.45000	41.51000
C	22.20000	38.47000	42.27000
C	22.84000	37.29000	42.71000
C	24.18000	37.21000	42.40000
C	24.97000	34.98000	43.39000
C	27.29000	38.38000	41.01000
C	27.25000	35.88000	42.31000
C	27.34000	34.86000	43.15000
C	28.67000	34.55000	43.13000
C	29.33000	33.51000	43.82000
C	30.71000	33.48000	43.70000
C	31.39000	34.44000	42.88999
C	30.78000	35.58000	42.32000
C	29.38000	35.54000	42.42000

Continued on next page

Table S32 – continued from previous page

C	28.46000	37.63000	41.23000
C	26.19000	34.35000	43.76000
N	25.05000	36.18000	42.63000
N	28.44000	36.46000	41.88000
O	23.88000	34.52999	43.64000
O	29.53000	38.13999	40.83000
H	24.70000	40.16000	40.73000
H	22.26000	40.30000	41.13000
H	21.14000	38.63999	42.43000
H	22.39000	36.46000	43.22000
H	27.33000	39.30000	40.45000
H	28.76000	32.78000	44.36000
H	31.27000	32.65000	44.12000
H	32.45999	34.35000	42.83000
H	31.37000	36.31000	41.79000
H	26.18000	33.58000	44.51000

Table S33: Coordinates (in Å) of the 8-fragment INDO Stack 5. Snapshot taken at $t = 49.5$ ns of the MD simulation in DMF.

C	27.51000	25.22000	16.39000
C	28.04000	24.01000	16.43000
C	29.40000	24.18000	16.44000
C	30.43000	23.25000	16.34000
C	31.74000	23.70000	16.29000
C	32.01000	25.10000	16.38000
C	30.96000	25.96000	16.32000
C	29.64000	25.51000	16.37000
C	28.12000	27.57000	16.31000
C	27.06000	22.97000	16.56000
C	26.27000	25.56000	16.49000
C	25.79000	26.78000	16.62000
C	24.45000	26.69000	16.71000
C	23.49000	27.74000	16.73000
C	22.19000	27.39000	17.04000
C	21.79000	26.06000	17.28000
C	22.72000	25.02000	17.25000
C	24.11000	25.38000	16.87000
C	25.63000	23.25000	16.65000
C	26.68000	27.85000	16.41000
N	28.46000	26.22000	16.42000
N	25.29000	24.60000	16.54000
O	29.00000	28.45000	16.21000
O	24.74000	22.36000	16.71000

Continued on next page

Table S33 – continued from previous page

H	30.18000	22.20000	16.22000
H	32.55000	23.03000	16.04000
H	33.03000	25.47000	16.39000
H	31.07000	27.03000	16.44000
H	27.38000	21.95000	16.63000
H	23.91000	28.72000	16.58000
H	21.53000	28.22000	17.25000
H	20.76000	25.88000	17.58000
H	22.49000	24.00000	17.50000
H	26.27000	28.79000	16.04999
C	28.06000	25.45999	19.83000
C	27.81000	26.76000	19.77000
C	26.46000	26.87000	19.98000
C	25.63000	28.06000	20.03000
C	24.31000	27.85000	20.29000
C	23.81000	26.54000	20.55999
C	24.66000	25.42000	20.57000
C	25.99000	25.59000	20.22000
C	27.15000	23.35000	20.52000
C	28.93000	27.60000	19.57000
C	29.23000	24.84000	19.92000
C	29.58000	23.60000	20.19000
C	30.91000	23.51000	20.21000
C	31.75000	22.43000	20.51000
C	33.15000	22.61000	20.51000

Continued on next page

Table S33 – continued from previous page

C	33.66000	23.82000	20.10000
C	32.83000	24.89000	19.77000
C	31.45000	24.69000	19.81000
C	30.29000	27.02000	19.57000
C	28.48000	22.75000	20.43000
N	27.01000	24.69000	20.18000
N	30.37000	25.61000	19.71000
O	26.16000	22.70000	20.80000
O	31.25000	27.68000	19.44000
H	26.06000	28.99000	19.70000
H	23.61000	28.66000	20.31000
H	22.75000	26.43000	20.71000
H	24.27000	24.46000	20.87000
H	28.81000	28.67000	19.54000
H	31.41000	21.47000	20.87000
H	33.87000	21.85000	20.77000
H	34.74000	23.87000	20.01000
H	33.34000	25.74000	19.34000
H	28.73000	21.75000	20.75000
C	29.63000	26.87000	23.08000
C	28.97000	27.99000	22.84000
C	27.67000	27.69000	23.13000
C	26.57000	28.49000	23.25000
C	25.33000	27.83000	23.63000
C	25.26000	26.45000	23.94000

Continued on next page

Table S33 – continued from previous page

C	26.42000	25.69000	23.91000
C	27.59000	26.34000	23.50000
C	29.44000	24.51000	23.47000
C	29.68000	29.17000	22.58000
C	30.94000	26.70000	22.95000
C	31.60000	25.53000	23.01000
C	32.91000	25.87000	22.79000
C	34.04000	25.02000	22.84000
C	35.28000	25.71000	22.68000
C	35.37000	27.13000	22.50000
C	34.17000	27.96000	22.35000
C	32.94000	27.31000	22.53000
C	31.13000	29.13000	22.53000
C	30.85000	24.35000	23.28000
N	28.86000	25.83000	23.30000
N	31.70000	27.82000	22.67000
O	28.66000	23.63000	23.72000
O	31.82000	30.11000	22.32000
H	26.61000	29.55000	23.02000
H	24.46000	28.41000	23.91000
H	24.34000	26.02000	24.31000
H	26.31000	24.64000	24.13000
H	29.17000	30.09000	22.36999
H	33.99000	24.07000	23.35000
H	36.16000	25.09000	22.75000

Continued on next page

Table S33 – continued from previous page

H	36.37000	27.54000	22.49000
H	34.20000	29.03000	22.27000
H	31.28000	23.38000	23.48000
C	30.05000	26.42000	26.63000
C	30.74000	27.51000	26.34000
C	29.84000	28.56000	26.37000
C	30.02000	29.89000	25.99000
C	28.86000	30.77000	26.06000
C	27.64000	30.29000	26.37000
C	27.47000	28.90000	26.63000
C	28.57000	28.03000	26.63999
C	27.81000	25.57000	26.99000
C	32.13000	27.33000	26.02000
C	30.53000	25.21000	26.68000
C	29.84000	24.10000	26.98000
C	30.71000	23.10000	26.91000
C	30.47000	21.70000	26.97000
C	31.62000	20.82000	26.80000
C	32.87000	21.40000	26.44000
C	33.08000	22.75000	26.18000
C	31.96000	23.63000	26.49000
C	32.70000	26.03000	26.10000
C	28.41000	24.28000	27.06000
N	28.69000	26.63999	26.77000
N	31.83000	24.98000	26.39000

Continued on next page

Table S33 – continued from previous page

O	26.65000	25.71000	27.31000
O	33.93000	25.74000	26.01000
H	30.93000	30.32000	25.63000
H	28.92000	31.84000	25.91000
H	26.82000	30.99000	26.46000
H	26.45000	28.56000	26.75000
H	32.65000	28.24000	25.75000
H	29.52000	21.26000	27.27000
H	31.60000	19.74000	26.77000
H	33.74000	20.82000	26.16000
H	33.97000	23.18000	25.74000
H	27.84000	23.42000	27.38000
C	28.84000	29.15000	29.81000
C	29.06000	30.44000	29.43000
C	27.87000	31.07000	29.60000
C	27.49000	32.40000	29.22000
C	26.23000	32.89000	29.55000
C	25.26000	31.96000	30.08000
C	25.59000	30.55000	30.28000
C	26.89000	30.16000	30.02000
C	27.18000	27.63000	30.66000
C	30.33000	30.76000	28.97000
C	29.71000	28.14000	29.82000
C	29.47000	26.86000	30.15000
C	30.70000	26.28000	29.91000

Continued on next page

Table S33 – continued from previous page

C	31.10000	24.88000	30.03000
C	32.44000	24.57000	29.67000
C	33.38000	25.58000	29.31000
C	32.96000	26.91000	29.20000
C	31.61000	27.26000	29.48000
C	31.32000	29.75000	28.92000
C	28.18000	26.55000	30.54000
N	27.53000	28.93000	30.22000
N	30.95000	28.48000	29.35000
O	26.11000	27.49000	31.17000
O	32.42000	29.99000	28.50000
H	28.24000	33.02000	28.75000
H	25.96000	33.93000	29.56000
H	24.26000	32.29000	30.31000
H	24.81000	29.88000	30.60000
H	30.62000	31.75000	28.66000
H	30.37000	24.26000	30.52000
H	32.70000	23.52000	29.73000
H	34.38000	25.35000	28.98000
H	33.67000	27.67000	28.91000
H	27.88000	25.54000	30.79000
C	28.86000	28.17000	33.54000
C	27.80000	27.48000	33.91000
C	28.23000	26.21000	34.07000
C	27.44000	25.15000	34.54000

Continued on next page

Table S33 – continued from previous page

C	28.09000	23.91000	34.67000
C	29.45000	23.78000	34.28000
C	30.23000	24.90000	33.86000
C	29.53000	26.09000	33.79000
C	31.15000	27.97000	32.90000
C	26.62000	28.25000	34.25000
C	28.94000	29.48000	33.36000
C	30.00000	30.16000	32.88000
C	29.55000	31.45000	32.72000
C	30.21000	32.63000	32.28000
C	29.44000	33.79000	32.22000
C	28.11000	33.84000	32.65000
C	27.51000	32.69000	33.16000
C	28.24000	31.50000	33.17000
C	26.63000	29.69000	34.07000
C	31.18000	29.41000	32.59000
N	29.97000	27.37000	33.41000
N	27.84000	30.23000	33.61000
O	32.17000	27.27000	32.80000
O	25.70000	30.44000	34.32000
H	26.37000	25.29000	34.62000
H	27.54000	23.03000	34.97000
H	29.93000	22.82000	34.42000
H	31.28000	24.86000	33.57000
H	25.70000	27.79000	34.57000

Continued on next page

Table S33 – continued from previous page

H	31.16000	32.61000	31.78000
H	29.92000	34.67000	31.82000
H	27.56000	34.77000	32.66000
H	26.48000	32.69000	33.47000
H	32.05000	29.94000	32.24000
C	27.57000	30.62000	36.95999
C	28.19000	31.75000	36.72000
C	27.29000	32.69000	36.86000
C	27.37000	34.09000	36.66000
C	26.19000	34.86000	36.71000
C	24.92000	34.23000	36.98000
C	24.88000	32.84000	37.21000
C	26.04000	32.08000	37.13000
C	25.43000	29.59000	37.51000
C	29.56000	31.66000	36.37000
C	28.08000	29.41000	37.02000
C	27.46000	28.24000	37.30000
C	28.43000	27.29999	37.32000
C	28.42000	25.94000	37.68000
C	29.61000	25.17000	37.72000
C	30.80000	25.73000	37.30000
C	30.81000	27.12000	36.98000
C	29.67000	27.87000	37.12000
C	30.15000	30.33000	36.50000
C	26.08000	28.28000	37.51000

Continued on next page

Table S33 – continued from previous page

N	26.25000	30.68000	37.26000
N	29.39000	29.26000	36.94000
O	24.20999	29.74000	37.69000
O	31.32000	30.02000	36.12000
H	28.28000	34.61000	36.39000
H	26.27000	35.90000	36.42000
H	23.99000	34.77000	37.01000
H	23.96000	32.36000	37.53000
H	30.18000	32.45999	35.99000
H	27.48000	25.45000	37.88000
H	29.57000	24.12000	37.96000
H	31.73000	25.20000	37.38999
H	31.71000	27.62000	36.67000
H	25.54000	27.36000	37.67000
C	26.69000	32.58000	40.27000
C	27.24000	33.79000	40.05000
C	26.20000	34.66000	40.18000
C	26.27000	36.04000	40.11000
C	25.05000	36.73000	40.29000
C	23.85000	36.02000	40.65000
C	23.84000	34.67000	40.77999
C	25.02000	33.97000	40.48000
C	24.65000	31.43000	40.69000
C	28.64000	33.84000	39.88999
C	27.34000	31.41000	40.38999

Continued on next page

Table S33 – continued from previous page

C	26.85000	30.24000	40.66000
C	27.85000	29.39000	40.74000
C	27.81000	28.02000	41.07000
C	29.02000	27.33000	40.91999
C	30.23000	27.94000	40.68000
C	30.27000	29.33000	40.43000
C	29.03000	30.05000	40.43000
C	29.42000	32.61000	39.99000
C	25.40000	30.20000	40.81000
N	25.31000	32.63000	40.34000
N	28.69000	31.44000	40.32000
O	23.41000	31.36000	40.88000
O	30.62000	32.56000	39.80000
H	27.16000	36.59000	39.85000
H	24.97000	37.80000	40.29000
H	22.95999	36.59000	40.86000
H	22.95000	34.08000	40.98000
H	29.07000	34.80000	39.65000
H	26.84000	27.59000	41.29000
H	29.01000	26.25000	40.87000
H	31.05000	27.25000	40.50000
H	31.24000	29.71000	40.12000
H	24.90000	29.29000	41.10000

Table S34: Coordinates (in Å) of the 8-fragment INDO Stack 6.

C	27.46835	25.28330	16.63265
C	27.94490	23.95636	16.71745
C	29.39300	24.11785	16.52661
C	30.45087	23.21851	16.49600
C	31.74000	23.70000	16.29000
C	31.97263	25.06394	16.11625
C	30.93330	25.99307	16.14145
C	29.65485	25.50774	16.34658
C	28.09295	27.59891	16.31015
C	27.00028	22.98558	16.93449
C	26.18104	25.60096	16.75297
C	25.70450	26.92790	16.66818
C	24.25639	26.76641	16.85902
C	23.19852	27.66575	16.88963
C	21.90940	27.18426	17.09563
C	21.67676	25.82032	17.26938
C	22.71610	24.89119	17.24418
C	23.99455	25.37652	17.03904
C	25.55644	23.28535	17.07548
C	26.64911	27.89868	16.45114
N	28.43023	26.22250	16.41692
N	25.21917	24.66177	16.96870
O	28.95037	28.44453	16.11694
O	24.69903	22.43973	17.26868

Continued on next page

Table S34 – continued from previous page

H	30.27454	22.15890	16.63045
H	32.57196	23.00856	16.26423
H	32.98461	25.41404	15.95751
H	31.10350	27.05083	16.00793
H	27.23851	21.93453	17.01834
H	23.37485	28.72536	16.75517
H	21.07744	27.87570	17.12140
H	20.66478	25.47023	17.42811
H	22.54589	23.83343	17.37770
H	26.41088	28.94973	16.36729
C	28.22928	25.50463	20.38879
C	28.01761	26.89902	20.31174
C	26.55203	27.00653	20.31833
C	25.68090	28.08685	20.26475
C	24.31000	27.85000	20.29000
C	23.81218	26.54970	20.36785
C	24.65912	25.44346	20.42311
C	26.02021	25.68606	20.39762
C	27.15672	23.33935	20.52054
C	29.14333	27.68111	20.25778
C	29.44166	24.95488	20.40941
C	29.65333	23.56049	20.48645
C	31.11891	23.45298	20.47987
C	31.99004	22.37266	20.53345
C	33.36094	22.60952	20.50820

Continued on next page

Table S34 – continued from previous page

C	33.85876	23.90981	20.43035
C	33.01182	25.01605	20.37509
C	31.65073	24.77345	20.40058
C	30.51422	27.12016	20.27766
C	28.52761	22.77840	20.54042
N	27.09266	24.75682	20.44091
N	30.57828	25.70269	20.35728
O	26.14188	22.66446	20.56749
O	31.52906	27.79505	20.23071
H	26.06329	29.09782	20.20426
H	23.62178	28.68400	20.24877
H	22.74147	26.39122	20.38607
H	24.28316	24.43318	20.48350
H	29.11354	28.75995	20.19701
H	31.60765	21.36169	20.59394
H	34.04916	21.77551	20.54943
H	34.92947	24.06830	20.41213
H	33.38778	26.02633	20.31470
H	28.55740	21.69956	20.60119
C	29.62578	26.86828	23.11345
C	28.96405	28.11027	22.99239
C	27.55921	27.75576	23.23793
C	26.38595	28.49831	23.26850
C	25.18415	27.85025	23.53649
C	25.15182	26.47610	23.77164

Continued on next page

Table S34 – continued from previous page

C	26.31110	25.70173	23.74827
C	27.50182	26.35212	23.48136
C	29.34439	24.50433	23.54787
C	29.75537	29.19349	22.70563
C	30.94126	26.72936	22.96263
C	31.60299	25.48738	23.08369
C	33.00782	25.84188	22.83815
C	34.18108	25.09934	22.80757
C	35.38288	25.74739	22.53959
C	35.41522	27.12155	22.30444
C	34.25594	27.89592	22.32781
C	33.06522	27.24552	22.59472
C	31.22265	29.09332	22.52821
C	30.81166	24.40415	23.37045
N	28.81286	25.81382	23.39823
N	31.75418	27.78383	22.67785
O	28.62097	23.55609	23.80295
O	31.94607	30.04156	22.27312
H	26.40674	29.56525	23.08649
H	24.26374	28.41862	23.56285
H	24.20436	25.99481	23.97794
H	26.29609	24.63742	23.92907
H	29.36855	30.19605	22.58840
H	34.16029	24.03239	22.98958
H	36.30330	25.17903	22.51322

Continued on next page

Table S34 – continued from previous page

H	36.36267	27.60284	22.09814
H	34.27095	28.96022	22.14701
H	31.19849	23.40160	23.48767
C	30.02549	26.42512	26.51523
C	30.82768	27.55128	26.22659
C	29.86291	28.65875	26.27388
C	29.98348	30.02927	26.08418
C	28.85063	30.82876	26.20072
C	27.61012	30.26819	26.50325
C	27.45470	28.89654	26.69966
C	28.58452	28.10800	26.58236
C	27.78720	25.66319	27.03069
C	32.15914	27.30653	26.00497
C	30.50894	25.18606	26.57486
C	29.70675	24.05990	26.86350
C	30.67152	22.95243	26.81621
C	30.55095	21.58191	27.00590
C	31.68380	20.78242	26.88936
C	32.92431	21.34300	26.58684
C	33.07973	22.71464	26.39042
C	31.94991	23.50319	26.50773
C	32.74723	25.94800	26.05939
C	28.37529	24.30465	27.08511
N	28.70971	26.70157	26.73002
N	31.82472	24.90961	26.36007

Continued on next page

Table S34 – continued from previous page

O	26.60749	25.90497	27.22401
O	33.92694	25.70622	25.86607
H	30.94467	30.46839	25.84919
H	28.93260	31.89785	26.05503
H	26.74343	30.91118	26.58859
H	26.49853	28.45338	26.93419
H	32.87471	28.08349	25.77522
H	29.58976	21.14279	27.24089
H	31.60183	19.71333	27.03505
H	33.79100	20.70000	26.50149
H	34.03590	23.15780	26.15589
H	27.65972	23.52769	27.31487
C	28.82814	29.18997	29.75552
C	29.12547	30.50739	29.34191
C	27.84928	31.20756	29.54346
C	27.44575	32.52033	29.33691
C	26.13414	32.87763	29.63378
C	25.23371	31.93626	30.13146
C	25.60649	30.61062	30.35022
C	26.91131	30.26245	30.05309
C	27.11037	27.72299	30.62350
C	30.40774	30.73231	28.90948
C	29.72290	28.20428	29.73675
C	29.42557	26.88686	30.15035
C	30.70176	26.18669	29.94881

Continued on next page

Table S34 – continued from previous page

C	31.10528	24.87392	30.15535
C	32.41689	24.51662	29.85849
C	33.31733	25.45799	29.36081
C	32.94454	26.78363	29.14204
C	31.63972	27.13181	29.43917
C	31.44067	29.67126	28.86877
C	28.14329	26.66195	30.58279
N	27.54807	28.99980	30.17870
N	31.00297	28.39445	29.31356
O	25.96493	27.54683	31.00365
O	32.58610	29.84742	28.48861
H	28.14166	33.25416	28.95072
H	25.80855	33.89756	29.47686
H	24.21857	32.23870	30.35481
H	24.91659	29.87474	30.73515
H	30.75788	31.69626	28.56789
H	30.40938	24.14009	30.54154
H	32.74249	23.49669	30.01541
H	34.33247	25.15555	29.13746
H	33.63444	27.51951	28.75711
H	27.79315	25.69799	30.92438
C	28.84779	28.17686	33.59627
C	27.69230	27.53205	34.09036
C	28.13051	26.13566	34.22304
C	27.49256	24.97900	34.65199

Continued on next page

Table S34 – continued from previous page

C	28.20071	23.78126	34.65987
C	29.53117	23.73657	34.24450
C	30.20083	24.87909	33.80820
C	29.49117	26.06586	33.80293
C	31.15193	27.87610	32.92048
C	26.59143	28.32673	34.28627
C	28.89260	29.48011	33.32780
C	30.04809	30.12492	32.83371
C	29.60989	31.52130	32.70104
C	30.24783	32.67797	32.27209
C	29.53968	33.87570	32.26420
C	28.20923	33.92039	32.67958
C	27.53957	32.77788	33.11588
C	28.24923	31.59111	33.12114
C	26.58847	29.78086	34.00360
C	31.14896	29.33023	32.63780
N	29.92233	27.36103	33.41288
N	27.81807	30.29594	33.51119
O	32.11770	27.15000	32.75425
O	25.62270	30.50696	34.16982
H	26.45984	25.00942	34.97524
H	27.71394	22.87373	34.99205
H	30.06082	22.79259	34.25978
H	31.23060	24.85442	33.48476
H	25.64868	27.95234	34.65981

Continued on next page

Table S34 – continued from previous page

H	31.28055	32.64755	31.94883
H	30.02646	34.78323	31.93203
H	27.67958	34.86438	32.66429
H	26.50980	32.80254	33.43932
H	32.09172	29.70463	32.26427
C	27.55868	30.57974	37.01952
C	28.28928	31.74359	36.69282
C	27.26058	32.79258	36.72247
C	27.29719	34.16235	36.49611
C	26.11979	34.89585	36.60484
C	24.91801	34.27069	36.93581
C	24.84675	32.89791	37.16931
C	26.02047	32.17513	37.05956
C	25.37508	29.69993	37.57987
C	29.63076	31.57268	36.46248
C	28.11586	29.37363	37.10561
C	27.38526	28.20978	37.43232
C	28.41396	27.16079	37.40266
C	28.37735	25.79102	37.62902
C	29.55475	25.05752	37.52029
C	30.75653	25.68268	37.18932
C	30.82779	27.05546	36.95583
C	29.65407	27.77825	37.06557
C	30.29946	30.25344	36.54526
C	26.04378	28.38069	37.66265

Continued on next page

Table S34 – continued from previous page

N	26.23090	30.78303	37.24204
N	29.44364	29.17034	36.88309
O	24.18497	29.87615	37.78028
O	31.48957	30.07722	36.34485
H	28.22807	34.65149	36.23896
H	26.13630	35.96370	36.43069
H	24.01533	34.86304	37.01437
H	23.92112	32.40502	37.42604
H	30.29633	32.38447	36.20464
H	27.44647	25.30188	37.88617
H	29.53824	23.98967	37.69445
H	31.65921	25.09033	37.11076
H	31.75342	27.54835	36.69909
H	25.37821	27.56890	37.92049
C	26.70684	32.59086	40.42056
C	27.36057	33.81846	40.17417
C	26.25563	34.78688	40.20213
C	26.19838	36.16433	40.03415
C	24.96579	36.80401	40.12158
C	23.80197	36.07840	40.37386
C	23.82464	34.69509	40.54734
C	25.05279	34.06586	40.45918
C	24.57463	31.52941	40.84856
C	28.71929	33.75672	39.99566
C	27.34909	31.42623	40.48112

Continued on next page

Table S34 – continued from previous page

C	26.69536	30.19863	40.72751
C	27.80030	29.23021	40.69955
C	27.85755	27.85276	40.86754
C	29.09014	27.21308	40.78010
C	30.25396	27.93869	40.52782
C	30.23129	29.32200	40.35435
C	29.00313	29.95123	40.44250
C	29.48130	32.48768	40.05312
C	25.33663	30.26038	40.90602
N	25.35964	32.68623	40.59320
N	28.69628	31.33086	40.30848
O	23.36753	31.60887	41.00401
O	30.68839	32.40822	39.89767
H	27.09952	36.73129	39.83815
H	24.90907	37.87686	39.99241
H	22.85492	36.59891	40.43734
H	22.92902	34.12479	40.74300
H	29.33189	34.62573	39.80069
H	26.95641	27.28580	41.06353
H	29.14685	26.14023	40.90928
H	31.20101	27.41818	40.46435
H	31.12691	29.89230	40.15868
H	24.72403	29.39136	41.10100
